# Rapid Electrodeposition of Defect‐Tuned MOF Nanoarchitectures: Synergistic Unlocking Electrochemical Performance via Dual Modulation of Morphology and Electronic Structure

**DOI:** 10.1002/advs.202520784

**Published:** 2025-12-26

**Authors:** Qing Wang, Zehui Yu, Sanghwa Yoon, Zitao Yang, Bongyoung Yoo

**Affiliations:** ^1^ Department of Materials Science and Chemical Engineering Hanyang University Ansan Republic of Korea; ^2^ College of Ecology and Resources Engineering Fujian Provincial Key Laboratory of Eco‐Industrial Green Technology Wuyi University Wuyishan Fujian China; ^3^ School of Chemistry and Chemical Engineering Nanjing University Nanjing Jiangsu China

**Keywords:** electrodeposition, energy storage, nanofragment‐amorphous MOF, oxygen evolution reaction, size‐dependent band gap

## Abstract

Metal‐organic frameworks (MOFs) are increasingly used in energy storage and the oxygen evolution reaction (OER), where surfaces of MOF typically undergo structural transformation into metal (oxy)hydroxides as the true active sites. Synthesizing ultrasmall or amorphous MOF nanoparticles enables precise activation over the structural reconstruction. This work rapidly and precisely tailors ultrafine and order‐disorder structure in MOF‐74 crystals using ligands 2,5‐dihydroxyterephthalic acid (H_4_dobdc) and competitive salicylic acid (SA) via electrodeposition. Electrodeposition rapidly produces nanofragment (2∼3 nm)‐amorphous MOF(Co)‐SA1. The introduction of a secondary nickel center electronically modulates the primary cobalt in MOF(Co_4_Ni_1_)‐SA1. X‐ray absorption fine structure (XAFS) spectroscopy confirms this structure, which facilitates a structural reconstruction. This reconstruction, evidenced in the redox region by in situ Raman spectra, results in superior performance and long stability for both energy storage and OER applications. Theoretical calculations reveal a reduced reaction energy barrier (from 1.59 to 0.50 eV) correlated with a smaller crystal size, and Ni promotes electron transfer between Co and ligands and lowing the potential of redox of Co. Thus, rapid electrodeposition combined with precise defect engineering within MOF crystals effectively tailors the crystal size, coordination environment of metal centers, and subsequent electrochemical reconstruction, offering a viable strategy for enhanced electrochemical applications.

## Graphical abstract

This work utilizes rapid electrodeposition and a competitive ligand to tailor crystal fragment/ amorphous MOF structures while Ni incorporation modulates the Co electronic environment. Their synergistic control over morphology and electronic structure accelerates reconstruction into active (oxy)hydroxides, lowers the Co^3+^/Co^4+^ oxidation potential, yielding improved energy‐storage and OER performance and offering a concise strategy for designing high‐efficiency MOF‐based electrocatalysts.

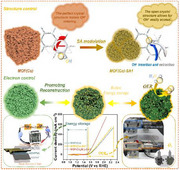

## 1 Introduction

Metal‐organic frameworks (MOFs), emerging as a distinctive category of porous coordination polymers, are constructed through the assembly of metal‐containing nodes interconnected by multifunctional organic ligands [1]. These hybrid materials exhibit exceptional structural versatility coupled with extraordinary porosity, manifested through ultrahigh specific surface areas, tunable pore geometries, and substantial void volumes [2]. Such unique architectural characteristics position MOFs as promising candidates for advanced energy storage applications, particularly in energy storage systems and catalytic systems [3]. Nevertheless, their inherent crystalline nature introduces fundamental limitations for electrochemical implementation. The periodic arrangement of redox‐active sites within rigid crystalline frameworks imposes spatial constraints on charge‐carrier accessibility, thereby impeding surface‐mediated Faradaic processes critical for energy storage mechanisms [4]. In addition, intrinsically poor electronic conductivity and the sluggish ion diffusion kinetics are caused by crystallographic confinement effects [5]. Addressing these challenges requires innovative material engineering strategies to reconcile the inherent crystallinity and crystal size with enhanced electrochemical functionality, representing a pivotal research frontier in next‐generation energy storage material development. Electrodepositing MOFs directly onto conductive substrates offers a promising strategy to address their inherent activity limitations. Furthermore, the rapid electrodeposition process significantly accelerates production. This approach ensures intimate contact between the MOF layer and the current collector, significantly improving charge transfer efficiency [6]. Electrodeposition facilitates rapid MOF synthesis through leveraging redox reactions, protonation/deprotonation processes, interfacial bonding, and electrostatic interactions, enabling precise control over MOF structure [7]. Wei et al., [8]. demonstrate the fabrication of ZIF‐8 MOF membranes via an aqueous cathodic deposition method, which enabled the formation of low‐defect‐density membranes at ambient temperature within a mere 60 min. Compared to conventional solvothermal methods, this technique dramatically reduces synthesis time while enhancing interfacial stability.

The incorporation of defect engineering during the electrochemical deposition of MOF materials can significantly improve their electrochemical properties. The introduction of structural defects, such as missing clusters or linker vacancies, can be achieved by employing similar ligands compared with the primary ligand during MOF synthesis [9]. Defective structures facilitate the regulation of crystal nucleation and growth, enabling control over MOF morphology and particle size [10]. Precise MOF morphologies are engineered through linker splicing, which modifies the coordination environment of metal centers, exposes additional active sites, and enhances charge transfer [11]. Dai et al. report a general, sustainable strategy for designing highly defective, ultrasmall tetravalent MOF (Zr, Hf) crystals (∼35% missing linkers, 4–6 nm) [12]. At this ultrasmall size, most atoms reside near the outer surface, featuring enlarged cavities compared to the bulk. This maximizes the substrate‐accessible interface while drastically shortening diffusion/desorption path lengths, thus boosting catalytic performance. In addition, incorporating two or more metal ions with different oxidation states within MOF frameworks creates mixed‐valence states due to metal coupling effects [13]. This alters electron occupancy and enhances the efficiency of their transformation into metal (oxy)hydroxides [14].

Therefore, this work presents a dual strategy of defect engineering and electronic modulation of metal centers to promote structural transformation and enhance activity in MOFs for energy storage and the oxygen evolution reaction (OER). Precise modulation using the primary ligand (H_4_dobdc) and a competitive modulator (SA) successfully opens the MOF crystal structure, generating nanocrystal fragments (2∼3 nm)‐amorphous morphologies that significantly reduce the bandgap (from 1.59 to 0.5 eV, as shown in DFT results). Introducing a secondary metal center (Ni) electronically modulates the primary metal (Co), accelerating the transformation to catalytically active metal (oxy)hydroxides. The high valence Co metal center decreases the potential of Co^4+^ oxidation and boosts OER performance (ƞ_10_ = 259 mV, ƞ_500_ = 318 mV in 1 m KOH). Simultaneously, efficient electron transfer toward Ni depletes the Co 3d orbitals, enhancing energy storage capabilities (delivering a specific capacitance of 917 F g^−1^ @1 A g^−1^ and 71% retention @50 A g^−1^ in a three‐electrode system). This study establishes a rapid, efficient electrodeposition approach combined with tailored defect engineering as a promising route for designing high‐performance energy storage and OER materials.

## 2 Results and Discussion

The experimental section details the synthesis protocols for both pristine and defect‐engineered MOFs. As illustrated in Figure S1, the monometallic MOF is synthesized by a facile and rapid electrodeposition method. In contrast to conventional solvothermal synthesis, the electrochemical deposition approach relies on the reduction of water or oxygen‐containing anions to electrochemically generate hydroxide ions (OH^−^). The cathodic production of OH^−^ establishes a localized pH gradient near the electrode surface [15]. In the presence of neutral bridging ligands and metal ions, this OH^−^ generation induces ligand deprotonation, facilitating direct MOF growth on the electrode substrate [16]. The chronoamperometric curve in Figure S1a exhibits a classical diffusion‐controlled nucleation process, which can be delineated into three distinct regimes. The initial stage (adjacent to the ordinate axis) corresponds to the formation of an electrochemical double layer at the substrate surface. The intermediate stage demonstrates a progressive current density increase until reaching a maximum, indicative of crystal nucleation and growth dynamics. The final regime displays a steady‐state current density, reflecting diffusion‐limited transport of depositing ions from the bulk solution to the current collector surface [17]. Complementarily, the linear sweep voltammetry (LSV) profile in Figure S1b reveals successive reduction peaks corresponding to metal ion reduction, OH^−^ ion generation, ligand deprotonation, and MOF crystallization, respectively [18]. Supporting information provides SEM images (Figure S2a–d) for the morphology growth of MOF(Co) at different deposition times. Combining with XRD spectra and HRTEM images results (Figure S2e,f) further demonstrates the successful electrodeposition of MOF‐74 crystals, and the more details analysis are shown in the Supporting Information. The FTIR spectroscopy (Figure S3) confirms ligand deprotonation during electrodeposition [19].

**FIGURE 1:**
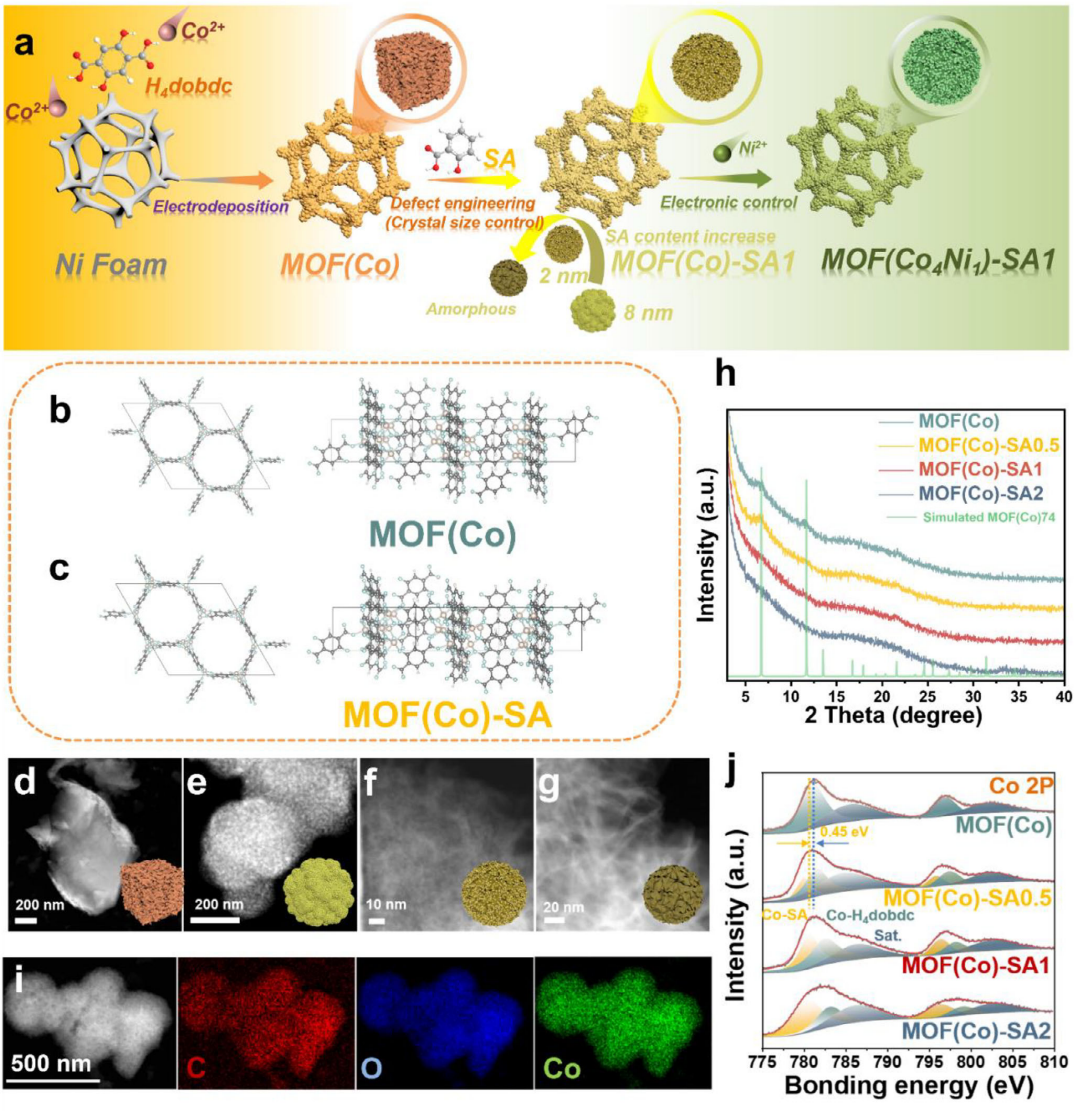
(a) Synthesis process of MOF(Co)‐*t*, MOF(Co)‐SA*x*, and MOF(Co*_y_* Ni*_z_*)‐SA1. (b, c) the crystal structure models of MOF(Co) and MOF(Co)‐SA, respectively. (d‐g) HAADF‐STEM images of MOF(Co), and MOF(Co)‐SA*x* (*x* = 0.5, 1, and 2), respectively. (h) XRD spectra of MOF(Co) and MOF(Co)‐SA*x* (*x* = 0.5, 1, and 2), respectively. (i) HRTEM‐mapping images of MOF(Co)‐SA1. (j) XPS spectra of Co 2p.

Figure 1a illustrates a facile, rapid electrodeposition strategy developed for MOF materials, effectively introducing defects by varying the amount of modulator SA. The crystal structures of pristine MOF(Co) and SA‐incorporated MOF are presented in Figure 1b,c, respectively. Substitution of the original linker H_4_dobdc by SA progressively opens the MOF(Co) framework, exposing Co metal sites. Supporting Information (Figures S4 and S5, and structure analysis of MOF(Co)‐SA*x*) details the impact of SA concentration on the electrodeposition process, resulting morphologies, and microstructure. Precise synthesis of nanocrystals with controlled dimensions is achieved by varying the amount of modulator SA in a mixture solvent (DMF: ethanol: deionized water). HAADF‐STEM analysis (Figure 1d–g) reveals the morphological evolution, where MOF(Co) without SA forms micron‐sized blocky crystals. Increasing SA content reduces nanocrystals to ∼8.83 nm in MOF(Co)‐SA0.5 and further down to 2–3 nm in MOF(Co)‐SA1, yielding a fragmented nanocrystalline/amorphous morphology. MOF(Co)‐SA2, with the highest SA loading, exhibits a fully amorphous structure (Figure S5i–l). Consistent with these results, Figure 1h shows the progressive attenuation of characteristic XRD diffraction peaks (220) and (300) of MOF(Co) with increasing SA, confirming the transition from crystalline to amorphous phases. Figure 1i and Figure S6 show elemental mapping images of MOF(Co) and MOF(Co)‐SA*x*, which demonstrate uniform distributions of C, O, and Co throughout the samples. It is worth noting that Co content decreases with increasing SA content. This occurs because the introduction of SA creates defects that terminate the connection between ligands and Co metal centers. Specific elemental compositions are listed in Table S1.

High‐resolution X‐ray photoelectron spectroscopy (XPS) reveals the electronic structures of MOF(Co) and its SA‐modified derivatives MOF(Co)‐SA*x* (Figure 1j). The Co 2p spectrum of MOF(Co) shows two characteristic peaks at 781.18/796.88 eV and 785.93/802.63 eV, corresponding to Co^2+^ (Co‐dobdc) and its satellite peaks, respectively. Utilizing mixed ligands to intentionally create defects (specifically, unsaturated metal sites) within the MOF structure. Based on these unsaturated Co sites, the high‐resolution XPS of the Co 2p region is used for probing the relative content of different cobalt sites. Therefore, the introduction of SA creates structural defects and decreases the electronegativity of adjacent metal cations (crystal structure opening, electron transfer to ligands), as indicated by the shift of Co 2p peaks toward lower binding energy [20]. The Co 2p spectrum of MOF(Co)‐SA*x* displays additional peaks at 779.98/795.83 eV, 781.58/797.53 eV, and 785.03/802.48 eV, representing two distinct Co^2+^ species (Co‐SA and Co‐dobdc) along with their satellites [21, 22]. As the SA content increases, the proportions of Co‐SA and Co‐dobdc in the Co 2p spectra of MOF(Co)‐SA*x* samples vary accordingly, as shown in Table S2 [23]. The Co‐SA content closely matches the initially added SA dosage in the MOF(Co)‐SA*x* samples, demonstrating precise electrochemical deposition control.

FTIR analysis reveals distinct vibrational features of the H_4_dobdc framework in the MOF(Co) and MOF(Co)‐Sa*x* (Figure S7d). The spectrum exhibits two prominent bands at 1552.4 and 1409.7 cm^−1^, corresponding to C = O vibrational modes, including asymmetric stretching. Characteristic absorptions at 871.7 and 811.8 cm^−1^ arise from C‐H bending vibrations within the aromatic ring, while the band at 1238.1 cm^−1^ represents C─O stretching in phenolate groups. Notably, the presence of Co─O coordination is confirmed by vibrational modes at 576.6 and 632.5 cm^−1^, collectively verifying the successful electrodeposition synthesis of the MOF material [24]. Progressive incorporation of SA induces significant spectral changes. The C═O stretching region undergoes peak splitting, reflecting modifications in metal coordination geometry. Furthermore, the systematic red‐shift of both C═O (1409.7 cm^−1^) and C─O (1238.1 cm^−1^) vibrational frequencies provides clear evidence for enhanced defect formation within the framework structure [25].

**FIGURE 2:**
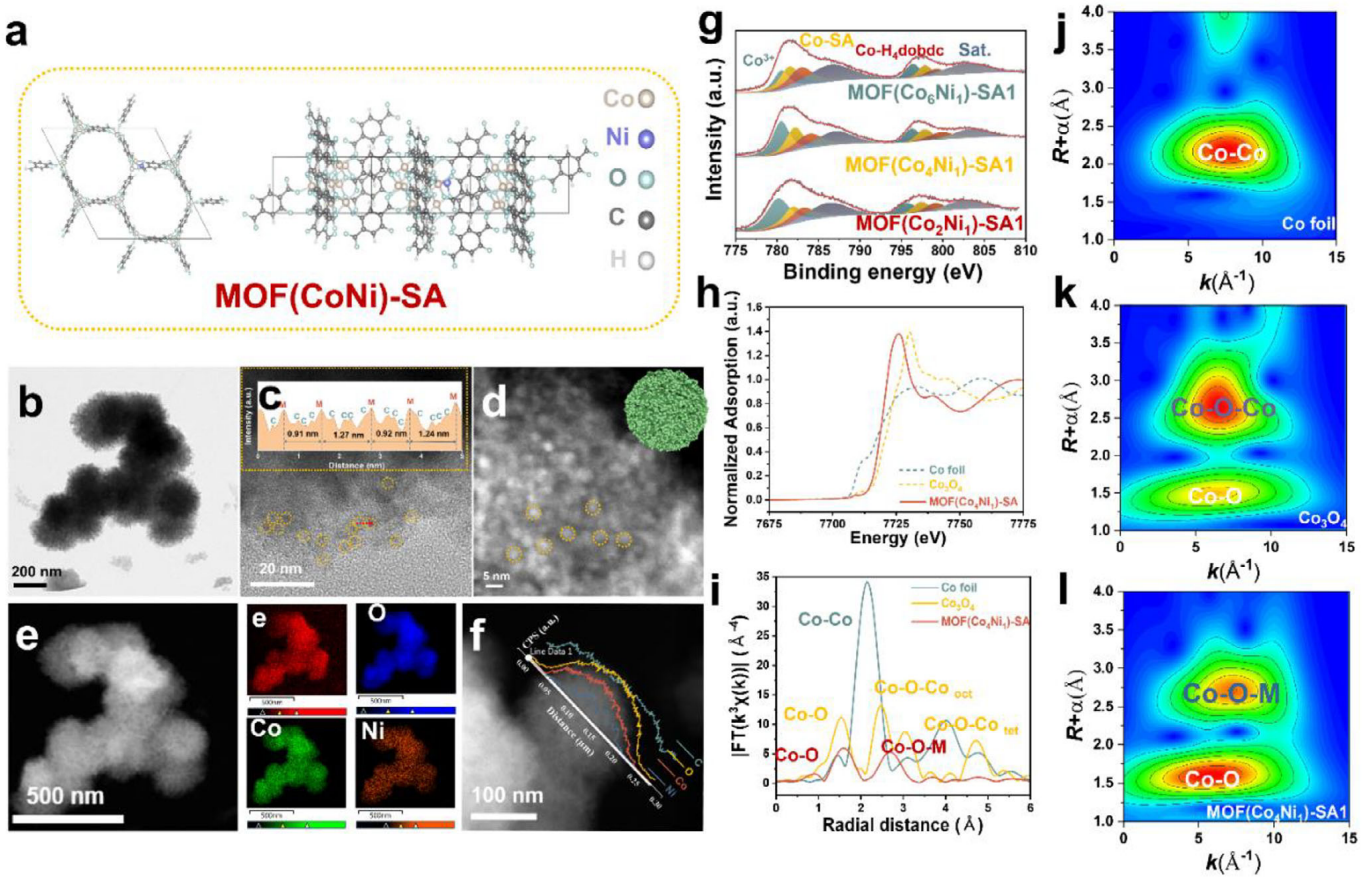
(a) The crystal structure models of MOF(CoNi)‐SA. (b, c) HRTEM images of MOF(Co_4_Ni_1_)‐SA1. (d)HAADF‐STEM image. (e, f) Mapping and line scan EDS images. (g) XPS spectra of Co 2p.(h) XAS characterization of Co foil, Co_3_O_4_, and MOF(Co4Ni1)‐SA: Normalized Co K‐edge XANES spectra with a Co foil, and Co_3_O_4_ as the references. (i) Fourier‐transformed EXAFS spectra of Co K‐edge. (j‐l) wavelet transforms for Co K‐edge EXAFS.

Based on MOF(Co)‐SA1, the strategic introduction of nickel (Ni) in the bimetallic MOF(Co*_y_* Ni*_z_*)‐SA1 optimizes the electronic structure of the host framework, thereby enabling a more efficient transformation into active metal (oxy)hydroxides and lowering the potential of further oxidation reaction. Figure 2a illustrates the crystal model of MOF(CoNi)‐SA. Incorporating Ni modulates the metal‐ligand bonding within the framework. Figure 2b,c shows the morphology and microstructure of MOF(Co_4_Ni_1_)‐SA1. Despite the inherent imaging blurriness of HRTEM, the image shows that the sample retains a nanocrystalline fragment‐amorphous structure. Line profiles across fragments (about 1.3 nm spacing for Metal‐dobdc, 0.91 nm for Metal‐SA) indicate ligand environments, with SA ligands at fragment edges. HAADF‐STEM confirms an actual fragment size of ∼3.8 nm (Figure 2d). Elemental mapping (Figure 2e) reveals uniform distributions of C, O, Co, and Ni, confirming successful Ni incorporation. Line scanning (Figure 2f) shows a Co: Ni ratio of 4:1, consistent with the metal salt feed ratio and confirming controllability of electrodeposition.

MOF(Co*_y_* Ni*_z_*)‐SA1 samples maintain spherical morphology (Figure S8a–c), with structural evolution observed in supporting data as Ni content varies. HRTEM (Figure S8d,g) shows distinct nanocrystals (approximately 5.5 nm) in MOF(Co_6_Ni_1_)‐SA1 (Co: Ni = 6:1 in bath). Crystal size decreases to about 3.8 nm with higher Ni^2+^ concentration (Co: Ni = 4:1 in bath, Figure S8e,h). Further increasing Ni^2+^ (Co: Ni = 2:1 in bath, Figure S8f,i) results in a predominantly amorphous microstructure. XRD patterns (Figure S9) confirm that crystallinity increases initially, then decreases with higher Ni^2+^ content. This trend arises due to the higher electronegativity of Ni, which stabilizes the structure by redistributing electrons from ligands to metal centers. NiCl_2_ and Co(NO_3_)_2_ are used to maintain a constant total metal salt concentration in plating solution. Consequently, increasing NiCl_2_ concentration proportionally decreases Co(NO_3_)_2_ concentration. Reduced NO_3_^−^ concentration affects deprotonation during electrodeposition, thereby influencing crystallization. HRTEM mapping‐EDS (Figure S10) demonstrates a decreasing metal content (At. %) in the MOF centers as NO_3_^−^ concentration decreases (i.e., Ni^2+^ concentration increases), with specific contents listed in Table S3. The Ni concentration governs electron modulation and nucleation/growth kinetics during electrodeposition, as evidenced by deposition profiles and the first co‐deposition step of Co/Ni and deprotonation in LSV curves (Figure S11).

XPS analysis corroborates this Ni‐induced electronic tuning. In the Co 2p spectra (Figure 2g), peaks at 780.53/796.43, 781.58/797.88, 783.03/799.28, and 786.73/803.33 eV correspond to Co^3+^, Co‐SA, Co‐dobdc, and their satellite peaks, respectively. Notably, the emergence of Co^3+^ and its gradual enrichment with increasing Ni content highlight Ni‐driven oxidation state modulation [26]. Simultaneously, the Ni 2p spectra exhibit peaks at 855.48/872.78, 857.03/874.98, and 861.33/879.73 eV, assigned to Ni‐SA, Ni‐dobdc, and satellite peaks [27], which confirms Ni incorporation (Figure S12a). A subtle shift of Co 2p peaks toward lower binding energy and Ni 2p peaks toward higher binding energy is observed in MOF(Co_6_Ni_1_)‐SA1 compared to MOF(Co_2_Ni_1_)‐SA1, further evidencing Ni‐mediated electronic redistribution between the two metal centers. The coordination ratios of Co and Ni determined from Co 2p and Ni 2p XPS spectra are provided in Tables S4 and S5.

Additionally, X‐ray absorption spectroscopy (XAS) analyzes the coordination environment of Co atoms in MOF(Co_4_Ni_1_)‐SA1. The Co K‐edge X‐ray absorption near‐edge structure (XANES) spectrum resembles that of Co_3_O_4_, indicating an average Co oxidation state between +2 and +3 (Figure 2h; Figure S13). This confirms effective modulation of the Co centers by the secondary Ni metal. The Fourier transform k^3^‐weighted extended X‐ray absorption fine structure (FT‐EXAFS) at the Co K‐edge (Figure 2i) reveals coordination dominated by the first coordination shell Co─O and Co─O─M (M = Co or Ni) coordination in the second coordination shell of MOF(Co_4_Ni_1_)‐SA1, with no detectable Co─Co aggregation signal. Notably, the amplitude of the Co─O peak at ≈1.6 Å attenuates, compared to that of Co_3_O_4_, indicating reduced coordination number and increased disorder. In the spectra of Co_3_O_4_, peaks at ≈2.5 and 3.1 Å correspond to octahedral (Co_oct_) and tetrahedral (Co_tet_) sites, respectively. The Co─O─M peak in MOF(Co_4_Ni_1_)−SA1 occurs at ≈2.69 Å, intermediate between the values of Co_oct_ and Co_tet_, which further demonstrates effective electronic modulation by Ni, involving electron transfer toward Ni, resulting in a higher Co oxidation state (+2< Co valence < +3), consistent with XPS results. The EXAFS spectrum of the Co K edge is analyzed, and suitable fitting results are obtained. (Figure S13, S14 and Table S5). Wavelet transform (WT) EXAFS contour plots (Figure 2j‐l) for Co foil, Co_3_O_4_, and MOF(Co_4_Ni_1_)−SA1 show dominant coordination environments consistent with the FT‐EXAFS data. The local atomic environment revealed by XAS is corroborated by the FTIR spectrum (Co─O and Ni─O bonds exist simultaneously), which supplements the overall molecular structure and bonding in the MOF(Co*_y_* Ni*_z_*)‐SA1 sample (Figure S15).

**FIGURE 3:**
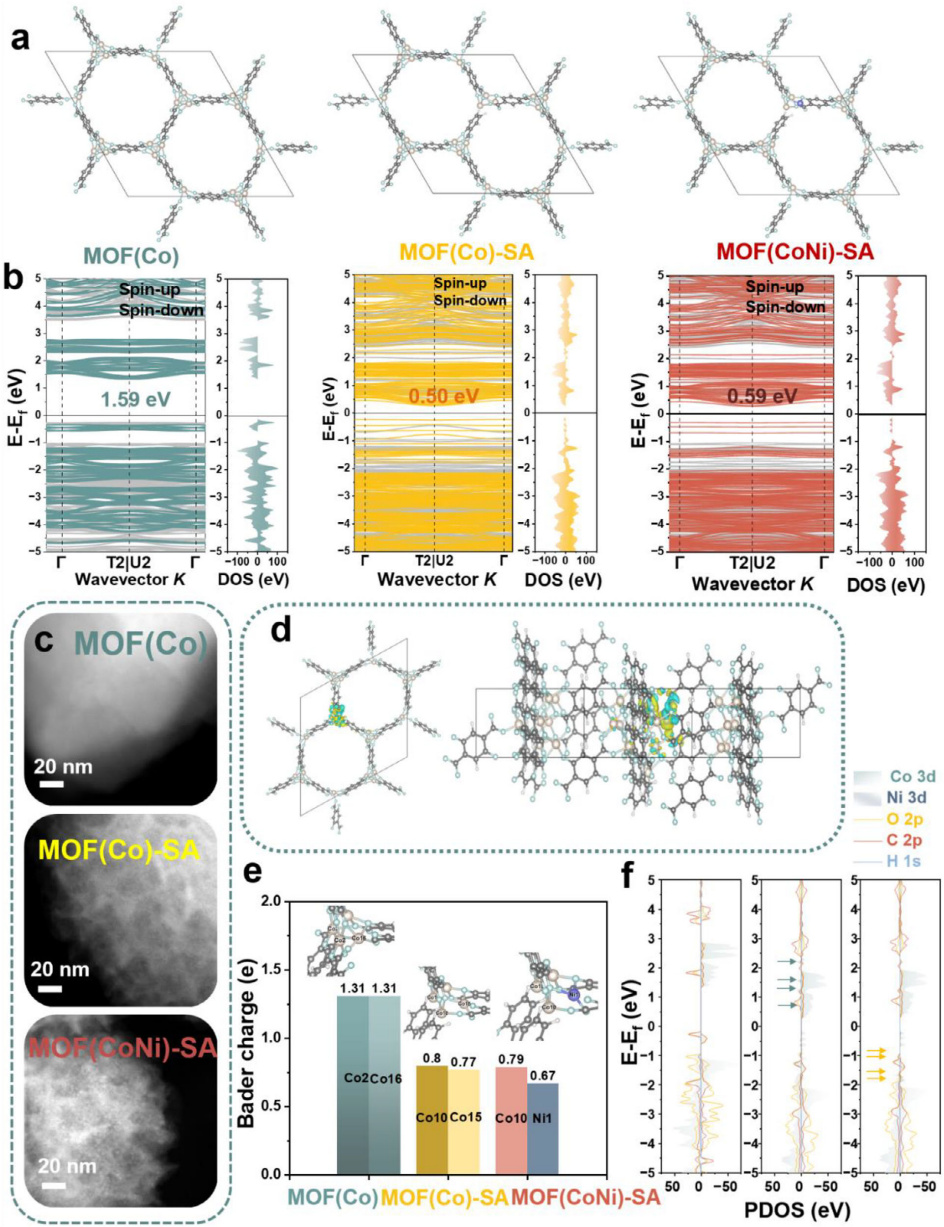
Theoretical calculation of pristine MOF(Co), MOF(Co)‐SA, MOF (CoNi)‐SA. (a) The optimized geometrical structures. (b) DFT‐calculated band structures and the DOS plots. (c) The HAADF‐STEM images. (d) Charge density difference plot of MOF (CoNi)‐SA. (e) Bader charge plot. (f) PDOS plots.

Density functional theory (DFT) calculations elucidate the mechanism by which defect engineering and electronic modulation accelerate the transformation of MOF (CoNi)‐SA into metal (oxy)hydroxides. Figure 3a shows the models of the pristine MOF(Co), the MOF(Co)‐SA after competitive ligand exchange, which replaces the original ligand with SA, and the MOF(CoNi)‐SA with the introduction of a second metal center, respectively, which are constructed based on preserving the crystal structure. Band structure analysis reveals that the morphological transformation from microcrystalline blocks to crystalline nanofragments with amorphous regions as a result of introducing the competitive ligand (SA), which drastically reduces the band gap of MOF(Co) from 1.59 to 0.50 eV (Figure 3b). This indicates that SA incorporation opens up the MOF structure, increases structural disorder, and lowers the reaction energy barrier. Further introduction of Ni to form MOF(CoNi)‐SA slightly increases the band gap to 0.59 eV, while the size of the nanoscale crystalline fragments increases from 2 to 3.8 nm (Figure 3c). These results establish a correlation between band gap, reaction energy barrier, and crystallite size, demonstrating that a more open, defective structure facilitates electron transfer and reduces energy barriers.

Although Ni incorporation slightly increases the band gap of MOF(CoNi)‐SA, it does not negatively impact the modulation of the reaction activity. Differential charge density analysis (Figure 3d) shows that Ni atoms, due to their higher electronegativity, exhibit charge depletion (electron‐rich states), while surrounding ligands accumulate charge (electron‐deficient states). This enhances local structural stability and increases the size of the nanoscale crystalline fragments. From Figure 3e, the Bader charge plot indicates a gradual decrease in the net positive charge near Co sites upon introducing SA and Ni, signifying electron transfer from Co to the ligands and Ni. This promotes the redox activity of Co^2+^. The presence of Ni further facilitates electron depletion from Co, rendering it more susceptible to oxidation and accelerating the formation of the active CoOOH and CoNiOOH phases, thereby enhancing catalytic and energy storage performance.

The PDOS plot (Figure 3f) reveals that the emergence of the spin‐up Co 3d peak in MOF(Co)‐SA compared to MOF(Co) signifies structural modulation of the MOF framework. This structural opening creates unoccupied states at the Co sites, indicating electron transfer to the ligands. Upon introducing Ni into MOF(Co)‐SA to form MOF(CoNi)‐SA, the spin‐up Co 3d peak above the Fermi level (unoccupied states) disappears, while the peak below the Fermi level (occupied states) attenuates. Concurrently, spin‐down Ni 3d states emerge in MOF(CoNi)‐SA, marking spin‐selective electron transfer from Co to Ni. This redistribution not only promotes the formation of essential high‐valence Co species in CoNiOOH but also enhances charge delocalization, favoring improved redox reversibility and energy storage performance. The observed spin‐selective electron redistribution from Co to Ni enhances the redox activity of the bimetallic system. Furthermore, the reduction in Co 3d occupied states and the increase in spin‐down Ni 3d states indicate efficient charge delocalization. This is crucial for achieving rapid and reversible redox reactions, which are a key requirement for high‐performance energy storage applications.

To investigate the effects of defect concentration and metal centers on the electrochemical response of MOF(Co), cyclic voltammetry (CV) measurements are performed using a three‐electrode system. The potential range encompassed both the electrode redox region (energy storage zone) and the oxygen evolution reaction (OER) region, without *i*R correction (Figure S16). As predicted by DFT calculations, MOF(Co_4_Ni_1_)‐SA1 exhibits significantly different redox potentials compared to MOF(Co) and MOF(Co)‐SA, along with superior OER performance. Therefore, the redox region is prioritized for analyzing the reactive process to elucidate the origin of the enhanced OER activity and apply this region to energy storage applications.

A series of electrochemical evaluations is conducted on MOF(Co)‐*t* (Figures S17–S20, Table S7). As indicated previously, increasing deposition time leads to a gradual rise in the quantity of these MOF(Co) structures along with their particle size. The CV curves of MOF(Co)‐*t* at different deposition times (Figure S17) reveal variations in redox potentials, implying size‐dependent reaction barriers. MOF(Co)‐900 exhibits superior electrochemical performance, highlighting the critical influence of deposition time on the final properties. These results underscore the importance of precise control over electrodeposition parameters for achieving optimal electrochemical performance. Electrochemical evaluation of MOF(Co)‐SA*x* (Figures S20 and S21) identifies the optimal SA incorporation level. The CV curves present two pairs of redox peaks, which can be corresponded to I/II (Co^2+^/Co^3+^) and III/IV (Co^3+^/Co^4+^) reactions. The underlying mechanism of energy storage can be illustrated through the following electrochemical redox reactions [6]:

(1) $$CoO+OH^{-}↔CoOOH+e^{-}$$

(2) $$CoOOOH+OH^{-}↔CoO_{2}+H_{2}O+e^{-}$$

Notably, the reduction peak in cyclic voltammetry (CV, Figure S21a) splits into two distinct peaks as SA content increases, attributed to SA‐induced structural defects that refine material dimensions and expose additional catalytically active Co sites [10]. The higher oxidation current in the CV of MOF(Co)‐SA1 suggests more facile oxidation of Co^2+^ to Co^3+^ and Co^3+^ to Co^4+^. Conversely, MOF(Co)‐SA0.5 exhibits the lowest oxidation current density despite its enhanced crystallinity, which validates the correctness of the DFT‐calculated reaction barrier of crystal size‐dependency. Although the amorphous MOF(Co)‐SA2 exposes more active sites, the reduced current density of redox peaks compared to MOF(Co)‐SA1 is due to excessive Co loss from disrupted coordination environments. Therefore, MOF(Co)‐SA1 exhibits the longest discharge duration (Figure S21b), corresponding to the highest specific capacity, along with superior rate capability (56.6% retention, Figure S21c). EIS reveals the intrinsic resistance (R_s_) and charge transfer resistance (R_ct_) [28]. A distinct equivalent circuit model is applied for MOF(Co)‐SA2 due to excessive SA‐induced defects, with data fitting performed using ZSimpWin software (Figure S21d). R_s_ decreases progressively with SA content, while R_ct_ and double‐layer capacitance (C_dl_) increase, consistent with SA‐mediated morphological modifications.

To investigate the kinetic processes occurring at the electrode and quantify the contributions of diffusion‐controlled and capacitive‐controlled processes, the linear dependence of peak current (i) on scan rate (v) in cyclic voltammetry (CV) curves is analyzed using the power‐law relationship*i* = *a* *v* ^*b*^ and *i*/*v* ^1/2^ = *k*_1_ *v* ^1/2^ + *k*_2_ [29]. As shown in Figures S22–S25, MOF(Co) exhibits b‐values of 0.77 (charge) and 0.76 (discharge), indicating a mixed charge storage mechanism involving both capacitive and diffusion‐controlled processes. With increasing SA content, crystallinity initially rises and then declines, which is confirmed by XRD spectra (Figure 1h). The b_charge_/b_discharge_‐values follow a similar trend: compared to MOF(Co)‐SA0.5 (0.81/0.82), MOF(Co)‐SA1 (0.75/0.77), and MOF(Co)‐SA2 (0.73/0.73) demonstrate progressively lower b‐values, which implies an increasingly dominant diffusion mechanism due to structural opening, facilitating CoOOH intermediate formation and lowering the reaction barrier.

**FIGURE 4:**
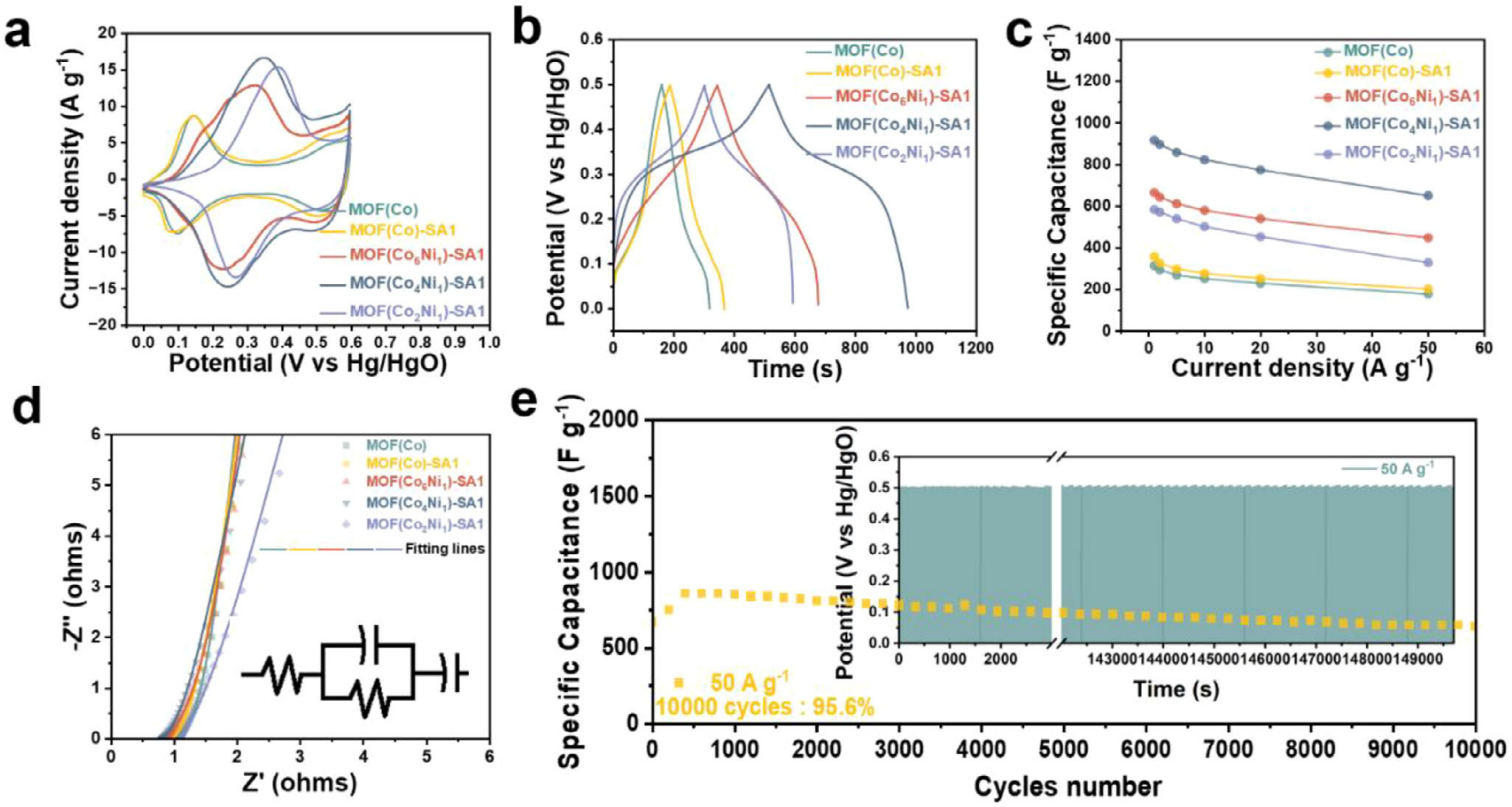
Electrochemical performance of MOF(Co), MOF(Co)‐SA1, and MOF (Co*_x_* Ni*_y_*)‐SA1. (a, b) CV and GCD curves. (c) Specific capacitance versus current density curves. (d) Nyquist plot and fitting lines. The inset shows the equivalent circuit model. (e) Cycling performance at a current density of 50 A g^−1^ with an inset showing GCD curves of 10 000 cycles.

Based on the SA modulation, electrochemical characteristics of bimetallic center MOFs with tunable Co/Ni ratios are systematically examined to elucidate Ni^2+^ introducing mechanisms (Figure S26). As demonstrated via DFT results (Figure 3), structural modulation via the SA strategy effectively tailors the morphology of MOF(Co) (from microcrystals to nanocrystalline fragments‐amorphous structures). This exposes additional active sites and lowers the reaction barrier for CoOOH formation. Furthermore, owing to the high electronegativity of Ni, which withdraws electrons from both Co centers and ligands. This electron redistribution accelerates CoNiOOH formation, and the high valence of the Co center facilitates oxidation, decreasing the potential of the Co^3+^/Co^4+^ redox transition [30, 31]. The MOF(Co_4_Ni_1_) electrode with an optimized Co/Ni ratio of 4:1 (Co_0.79_Ni_0.21_) demonstrates the largest integrated redox peak area (Figure 4a). The CV profiles unambiguously confirm that defect engineering and metal center adjusting substantially enhance the electrochemical performance of MOF(Co*_y_* Ni*_z_*)‐SA1.

The gradual disappearance of the redox peak current density for Co^2+^ and Co^3+^ in Figure 4a confirms efficient directional electron transfer from Co to Ni centers, promoting rapid generation of the active CoNiOOH phase. Consequently, the oxidation reaction of Co^3+^ to Co^4+^ is activated, enabling enhanced capacity. However, excessive Ni^2+^ (Ni choride salt) introduces metal center loss by interfering with protonation‐dependent MOF crystallization. Thus, specific capacity follows a first increased and then decreased trend with increasing Ni content. Therefore, MOF(Co_4_Ni_1_)‐SA1 achieves the longest discharge duration, indicating optimal charge storage capability. The specific capacitances (Figure 4b) of MOF(Co), MOF(Co)‐SA1, and MOF(Co*_y_* Ni*_z_*)‐SA1 series are 315.2, 359.0, 666.0, 917.0, and 584.0 F g^−1^, respectively.

The GCD curves of MOF(Co*_y_* Ni*_z_*)‐SA1 exhibit quasi‐symmetric shapes (Figure S26), which are consistent with the result of CV curves, confirming superior electrochemical reversibility and distinct charge/discharge plateaus aligned with CV redox features. The calculated discharge capacitances (Figure 4c) according to GCD curves (Figure S18, S20, and S26) show that the MOF(Co_4_Ni_1_)‐SA1 delivers specific capacitances of 917.0, 897.2, 859.0, 823.0, 774.0, and 651.0 F g^−1^ at current densities of 1–50 A g^−1^, respectively, retaining 71% capacity retention at 50 A g^−1^, highlighting exceptional rate capability. EIS measurements under open‐circuit conditions reveal that MOF (Co_4_Ni_1_)‐SA1 exhibits the minimum charge transfer resistance (R_ct_) and maximum electric double layer capacitance (C_dl_) (listed in Table S9). This demonstrates the synergistic effect between SA‐mediated structural defect regulation and Ni^2+^ concentration‐dependent electronic structure modulation. The combined mechanisms enhance reactive site availability, promote the formation of a truly reactive center of CoNiOOH, reduce reaction energy barriers of Co^2+^/Co^3+^ and Co^3+^/Co^4+^, and establish efficient electron transfer pathways during charge/discharge processes. It is worth noting that the cycling stability evaluation of MOF (Co_4_Ni_1_)‐SA1 is performed through 10 000 charge–discharge cycles at a high current density of 50 A g^−1^. The material retains 95.6% of its initial capacitance after prolonged cycling, highlighting its outstanding structural durability and electrochemical performance.

Regarding the kinetic impact of Ni^2+^ content in MOF(Co*_y_* Ni*_z_*)‐SA1, Ni center incorporation increases the b‐values compared to MOF(Co)‐SA1. Specifically, MOF(Co_6_Ni_1_)‐SA1 exhibits b‐values of 0.89 (charge) and 0.90 (discharge), while MOF(Co_4_Ni_1_)‐SA1 shows 0.84 and 0.88, and MOF(Co_2_Ni_1_)‐SA1 displays 0.79 and 0.84. Beyond a minor increase in nanocrystalline fragment size, Ni centers enhance redox kinetics between Co^3+^ and Co^4+^ through electron‐withdrawing effects. This electron depletion from Co centers (validated by DFT calculations) generates high‐valence Co with unoccupied 3d orbitals, shifting the charge storage mechanism from diffusion‐controlled battery‐type toward surface‐dominated processes (Figures S27–S29). Consequently, rate capability improves while structural robustness is maintained.

**FIGURE 5:**
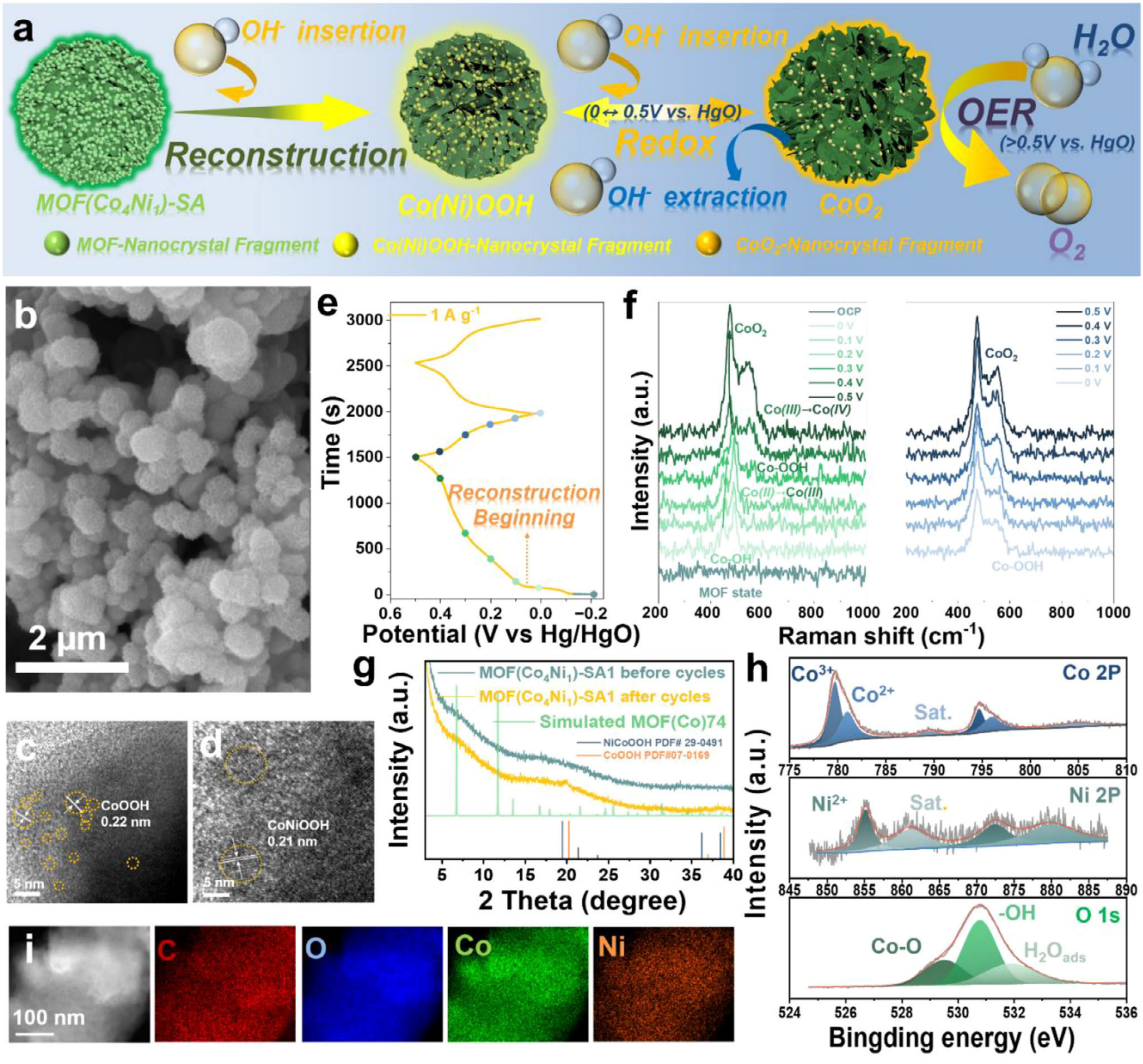
(a) Schematic diagram of Co(Ni)OOH reconstruction, redoxing process, and OER. (b) The SEM images of MOF (Co_4_Ni_1_)‐SA1 after 10 000 cycling. (c, d) HR‐TEM images. (e, f) The GCD curve and its in situ Raman spectra of MOF (Co_4_Ni_1_)‐SA1. (g) The XRD spectra of MOF (Co_4_Ni_1_)‐SA1 after 10 000 cycling. (h) XPS spectra. (i) Mapping images.

During electrochemical cycling, the nanocrystalline fragment‐amorphous structure of MOF(Co_4_Ni_1_)‐SA1 undergoes surface reconstruction, forming lamellar CoNiOOH through interaction with OH^−^ ions, which serves as the active phase. The amorphous structure implies a more open MOF framework with greater exposure of metal centers, facilitating CoNiOOH formation, which may lead to a coexistence of amorphous and crystalline CoNiOOH phases. The primary redox reaction is subsequently carried out (Figure 5a). SEM analysis (Figure 5b) reveals that cycled electrodes retain their spherical particle morphology despite developing a lamellar surface texture. HRTEM images (Figure 5c,d) directly evidence the formation of ultrafine CoOOH and Co(Ni)OOH fragments. The reconstruction of these nanoscale features was completed on the MOF(Co_4_Ni_1_)‐SA1 and reveals the redox process before OER.

In situ Raman spectroscopy is further employed to elucidate the mechanism of the in situ transformation of the MOF(Co_4_Ni_1_)‐SA1 electrode. Figure 5e displays the GCD curves for the first and second cycles. In situ Raman spectra (Figure 5f) reveal no characteristic peaks in the Raman curve of MOF (Co_4_Ni_1_)‐SA1 at the open circuit potential (OCP). An irreversible plateau observed during the first charging process (Figure 5e) corresponds to the reconstruction beginning of Co(Ni)OOH on the surface of MOF (Co_4_Ni_1_)‐SA1. In situ Raman spectra conducted during the first GCD cycle show that, within the potential range from OCP to 0 V (vs. HgO), OH^−^ ions incorporate into the MOF(Co_4_Ni_1_)‐SA1, leading to its transformation into a hydroxide. Two characteristic peaks appear at 449 and 493 cm^−1^, assigned to the A_1g_ and A_2u_ modes of Co(Ni)(OH)_2_, respectively (Figure 5f). Upon applying more positive potentials (0–0.3 V), two additional peaks are observed at 488 and 559 cm^−1^, corresponding to the A_1g_ and E_g_ vibrational modes of Co–O in Co(Ni)OOH, indicating a structural transformation to Co(Ni)OOH. With further increase in potential (0.3–0.5 V), two new peaks emerge at 475 and 545 cm^−1^, attributed to the A_1g_ and E_g_ modes of Co(Ni)O_2_. This suggests the formation of high‐valence Co^4+^–O species in the redox region before OER [32]. During the discharge process, as the potential decreases (0.5–0 V), Co(Ni)O_2_ is gradually reduced back to Co(Ni)OOH. Notably, the reconstruction plateau observed in the first cycle is absent in the second‐cycle GCD profile of MOF MOF (Co_4_Ni_1_)‐SA1, which means the complete reconstruction of MOF (Co_4_Ni_1_)‐SA1, corresponding to the in situ Raman spectra. (Figure 5e).

XRD patterns (Figure 5g) confirm preservation of the original MOF framework after cycling, while exhibiting a characteristic peak at 19.9° corresponding to CoOOH (PDF#07‐0169). The slight leftward shift of this peak versus the reference indicates effective Ni incorporation. Concurrently, a broad diffraction feature emerges at 38.3°, attributable to nanocrystalline CoNiOOH (PDF#29‐0491) with minimal domain size or amorphous state. Finally, high‐resolution XPS spectra of Co 2p, Ni 2p, and O 1s corroborate the coexistence of both CoOOH and CoNiOOH phases (Figure 5h). Elemental mapping images show Ni uniformly enriched in the MOF matrix (Figure 5i). These findings collectively verify that Ni^2+^ serves as an electronic modulator without direct involvement in electrochemical processes [33].

Although MOF(Co_4_Ni_1_)‐SA1 demonstrates excellent rate stability, its capacity experiences slight decay. Under high‐current cycling, continuous OH^−^ insertion disrupts the nanocrystalline fragment‐amorphous structure, causing irreversible formation of CoOOH lamellae that compromise structural integrity (Figure S30a). HRTEM reveals lattice spacing corresponding to the (110) plane of CoOOH, supported by FFT analysis (Figure S30b). Figure S30c further shows Co enrichment within hexagonal CoOOH nanosheets, while Ni remains predominantly in the MOF framework. EDS quantification confirms this phase segregation, revealing a Co: Ni ratio of 3.4:1 in degraded regions (position 1), which elemental redistribution represents the primary origin of capacity fade.

To evaluate the practical performance of the MOF(Co_4_Ni_1_)‐SA1 electrode, an asymmetric supercapacitor (ASC) device is constructed using MOF(Co_4_Ni_1_)‐SA1 as the cathode and commercial reduced graphene oxide (rGO) with 10% oxygen‐containing functional groups (three‐electrode performance data in Figure S31) as the anode in 2 m KOH electrolyte (Figure S32a). Cyclic voltammetry (CV) curves of rGO (−1 to 0 V) and MOF(Co_4_Ni_1_)‐SA1 (0–0.6 V) at 10 mV s^−1^ (Figure S32b) suggest a theoretical operational voltage of 1.6 V for the ASC. It is worth noting that the ASC exhibits negligible polarization and water decomposition within 0–1.7 V (Figure S32c), attributed to reasonable electrode mass and specific capacity matching of cathode and anode electrodes. Symmetrical GCD profiles within this voltage range confirm stable electrochemical behavior (Figure S32d). Quasi‐rectangular CV curves (Figure S32e) with retained shape up to 100 mV s^−1^ indicate excellent electrochemical reversibility and rapid ion/electron transport. GCD testing at varying current densities (Figure S32f) yields a maximum specific capacitance of 104.4 F g^−1^ with 57.9% rate capability at 20 A g^−1^ (Figure S32g). The ASC demonstrates exceptional cycling stability, maintaining 91.1% capacitance after 49 000 cycles at 20 A g^−1^ (Figure S32h), inheriting the durability of the MOF(Co_4_Ni_1_)‐SA1 cathode from three‐electrode tests. EIS analysis (Figure S31i) reveals increased series resistance (R_s_: 1.52 to 1.55 Ω, Error%: 1.22% and 0.821%) and charge transfer resistance (R_ct_: 0.83 to 1.11Ω, Error%: 2.85% and 1.79%) post‐cycling, potentially due to structural degradation and electrolyte evaporation during prolonged operation.

**FIGURE 6:**
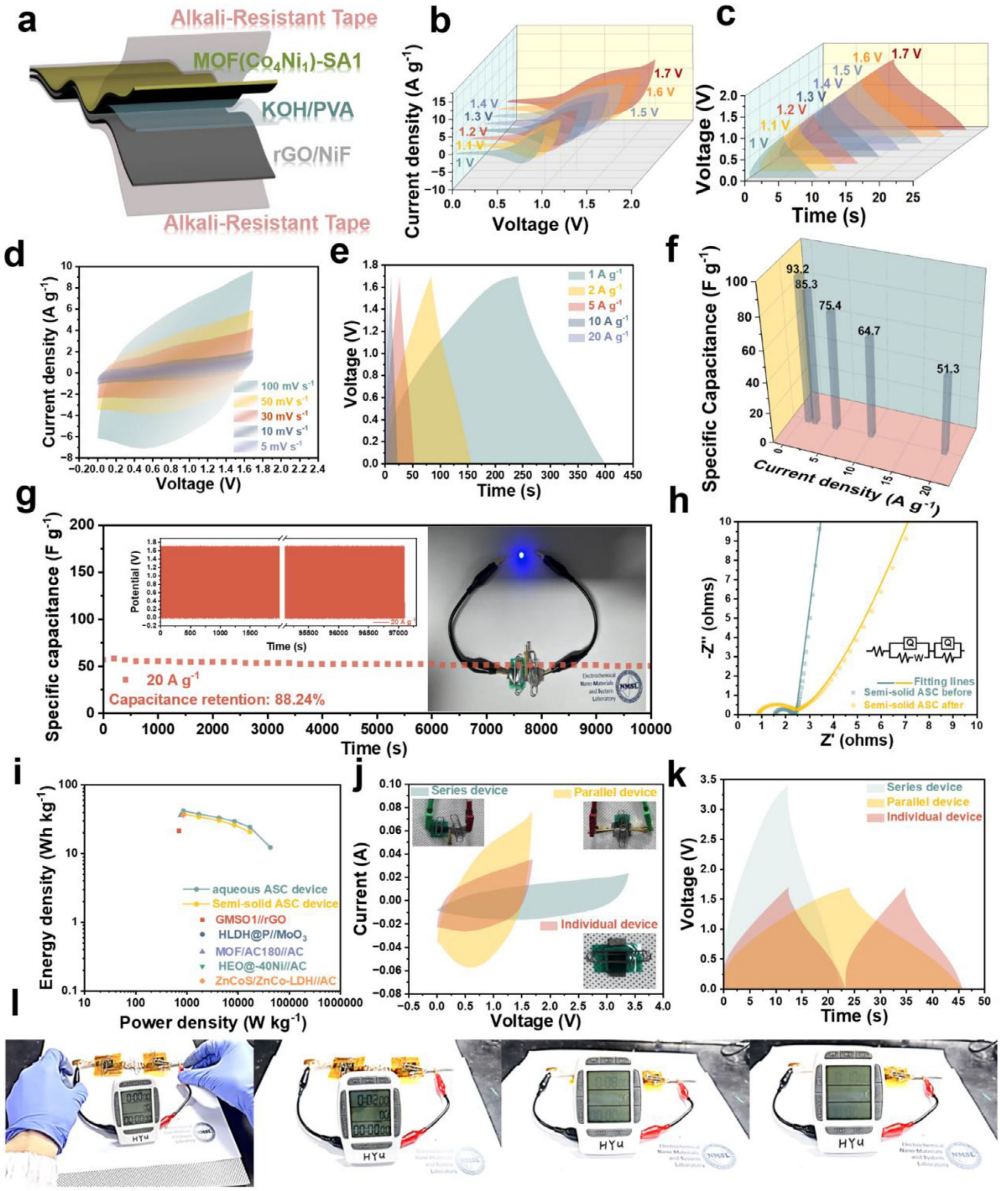
Electrochemical performance of MOF (Co_4_Ni_1_)‐SA1 quasi‐solid‐state ASC device. (a) ASC structure; (b) Voltage window optimization of CV at 100 mV s^−1^. (c) The GCD curves under different voltages at 10 A g^−1^. (d) The CV curves at various scan rates (0–1.7 V). (e) GCD curves at different current densities. (f) Specific capacitance. (g) Cycling stability at 20A g^−1^ with the inset showing GCD curves of 10 000 cycles and the photo of ASC power a blue LED light (rated voltage: 3 V). (h) EIS before/after 10 000 cycles with equivalent circuit (inset). (i) Ragone plot of aqueous ASC and quasi‐solid‐state ASC device. (j and l) CV and GCD curves of individual, series, and parallel quasi‐solid‐state ASC device. (k) Two series‐connected ASC devices power a timer with a rated voltage of 3 V for 10 min.

A quasi‐solid‐state ASC device is fabricated using MOF (Co_4_Ni_1_)‐SA1 as the cathode, commercial rGO as the anode, and PVA/KOH gel electrolyte to satisfy diverse application requirements (Figure 6a). The device demonstrates an operational voltage range identical to its aqueous device in both CV (Figure 6b) and GCD profiles (Figure 6c) across varied scan rates and current densities. However, the inferior wettability of gel electrolyte compared to aqueous systems necessitates an activation process before stable operation. Quasi‐rectangular CV curves (Figure 6d) and symmetrical GCD curves (Figure 6e) confirm preserved electrochemical reversibility despite reduced ionic mobility in the gel matrix. The quasi‐solid‐state ASC achieves a maximum specific capacitance of 93.2 F g^−1^ (Figure 6f). The rate performance of 55%, which is lower than that of the aqueous device at 20 A g^−1^, reflects inherent limitations in ion transport through polymeric electrolytes. Cycling tests reveal 88.24% capacitance retention after 10 000 cycles (Figure 6g), with two series‐connected devices successfully powering a 3 V blue LED. EIS analysis shows Rs decreased (R_s_: 1.52 to 0.82 Ω, Error%: 2.19% and 2.78%) post‐activation due to improved electrolyte connection, while subsequent R_ct_ increase (from 0.91 to 1.68 Ω, Error%: 5.20% and 2.87%) during cycling arises from electrode structural collapse and electrolyte dehydration (Figure 6h). The quasi‐solid‐state ASC delivers a maximum energy density of 37.41 Wh kg^−1^ at 850 W kg^−1^, slightly lower than that of the aqueous system (41.91 Wh kg^−1^) but superior to recent related research [34, 35, 36, 37, 38] (Figure 6i and Table 1). Scalability is demonstrated through voltage/capacitance enhancement via series or parallel device integration (Figure 6j,k) [39], with two series‐connected devices powering a 3 V timer for 10 min (Figure 6l).

**TABLE 1:** Electrochemical Performance of ASC Devices Based on MOFs and MOF‐Derived Electrode Materials.

Samples	Electrolyte	Energy density (Wh kg^−1^)	Power density (W kg^−1^)	Stability (Capacitance retention@cycles@current density)	References
MOF/AC‐180 (CoNi)	KOH	35.9	750	91%@10 000@10 A g^−1^	[36]
MXene@NiCo‐MOF	PVA/KOH	40.23	1495.07	84.4%@10 000@4 A g^−1^	[52]
Co‐Ni/rGO	KOH	39.58	208.33	87.4%@10 000@10 A g^−1^	[53]
Ni‐MOF@MX_2_	KOH	48.2	750	94%@10 000@20 A g^−1^	[54]
NiCo_2−_ *_x_* Mn*_x_* O_4_@Ni‐MOF‐1	PVA/KOH	33.8	750	106%@5000@ 15 mA g^−1^	[55]
Fe‐MET	PVA/KOH	23.33	375	72%@5000@ 4A g^−1^	[56]
Ni_3_S_4_/Co_3_S_4_	KOH	28.6	800	82.8%@7000@ 1 A g^−1^	[57]
La(Mn_0.2_Fe_0.2_Co_0.2_Ni_0.2_Cu_0.2_)O_3_	PVA/KOH	31.2	725	84.2 %@10 000@ 5 A g^−1^	[58]
**MOF(Co_4_Ni_1_)‐SA1**	**KOH**	**41.9**	**850**	**91.1%@49 000@20A g^−1^**	**This**
	**PVA/KOH**	**37.4**	**850**	**88.2%@10 000@20A g^−1^**	**Work**

The redox reactions in the redox region serve to verify that transition metal‐based oxyhydroxides undergo further deprotonation, yielding high‐valence species that are considered the active entities for the OER. As discussed in the charge storage region, Co^4+^ species form before the OER potential, indicating that Co^4+^ species are key intermediates [40]. However, the conversion of CoOOH to CoO_2_ is thermodynamically unfavorable, requiring a higher applied potential to drive the oxidation of CoOOH and subsequent OER catalysis [41]. In the electron storage region (from CV, Figures S16 and S34a), MOF(Co) reduces the reaction energy barrier for the conversion of Co‐O to CoOOH by modulating ligand defects via SA strategies to expose more metal sites. Based on the DFT calculation results, in MOF(Co_4_Ni_1_)‐SA1, the Ni metal center withdraws electrons from both the Co metal centers and the ligands, which further promotes the formation of Co(Ni)OOH. The electron‐deficient high‐valent Co facilitates oxidation and lowers the Co^3+^/Co^4+^ redox potential (proof by Raman spectra at 1.525 V (vs. RHE), Figure S34b) [31]. Consequently, MOF(Co_4_Ni_1_)‐SA1 exhibits significantly enhanced OER performance in Figure S16.

**FIGURE 7:**
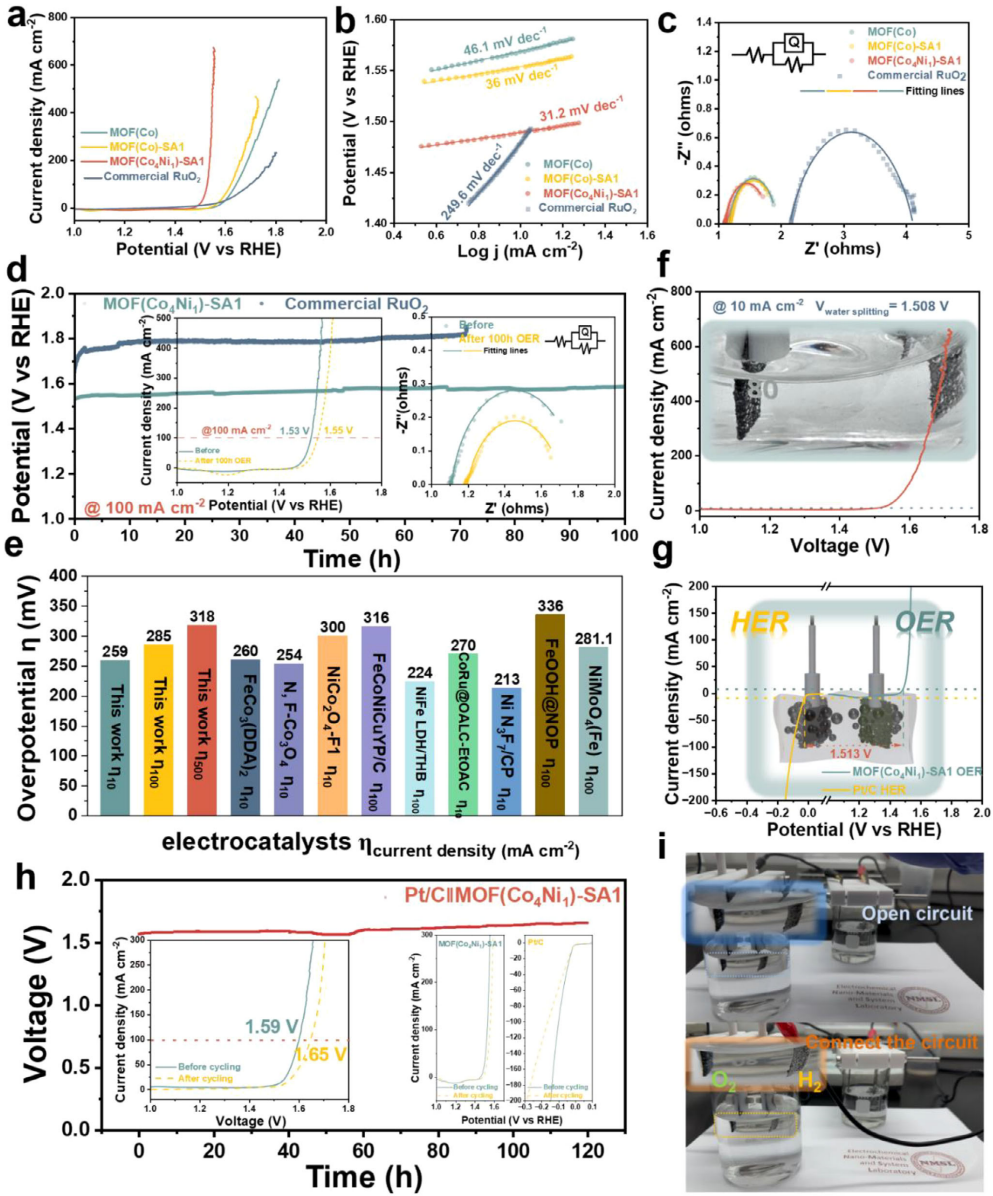
(a) OER polarization curves of MOF(Co), MOF(Co)‐SA1, MOF (Co_4_Ni_1_)‐SA1, and commercial RuO_2_ with 80% *iR*‐correction. (b) Corresponding OER Tafel plots. (c) EIS plots. (d) Long‐term stability of MOF (Co_4_Ni_1_)‐SA1 and commercial RuO_2_ at 100 mA cm^−2^ for 100 h. (e) Comparison of the overpotential with other advanced OER electrocatalysts. (f) The polarization curve of overall water splitting. (g) The water‐splitting voltage is estimated in a three‐electrode system. (h) Long‐term stability of water splitting at 100 mA cm^−2^ for 120 h. The insets show the LSV curve after stability testing. (i) The photograph of the water splitting cell is powered by an asymmetric supercapacitor.

As shown in the LSV polarization curves of MOF(Co), MOF(Co)‐SA1, MOF(Co_4_Ni_1_)‐SA1, and commercial RuO_2_ with 80% *i*R‐correction in Figure 7a. MOF(Co_4_Ni_1_)‐SA1 exhibits high OER activity in 1 m KOH, achieving an overpotential of 259 mV at 10 mA cm^−2^, which approaches that of commercial RuO_2_ (ƞ_10_ = 249 mV). Furthermore, MOF(Co_4_Ni_1_)‐SA1 demonstrates exceptional performance at high current densities, with overpotentials of ƞ_100_ = 297 mV and ƞ_500_ = 318 mV. To validate the rationality of *i*R correction, the OER polarization curves without *i*R‐correction are presented in Figure S35, and the corresponding uncorrected performance parameters are summarized in Table S12. Probing the reaction kinetics, Tafel plots are constructed (Figure 7b). The Tafel slope for MOF(Co) is 46.1 mV dec^−1^. This value decreases significantly to 36.0 mV dec^−1^ for MOF(Co)‐SA1 and further to 31.2 mV dec^−1^ for MOF(Co_4_Ni_1_)‐SA1, indicating that MOF(Co_4_Ni_1_)‐SA1 possesses the most efficient kinetics within this series. The Nyquist plot of MOF(Co_4_Ni_1_)‐SA1 displays the smallest semicircle compared to MOF(Co) and MOF(Co)‐SA1 (Figure 7c, specific value of R_s_ and R_ct_ listed in Table S13). This corresponds to a decrease in the R_ct_ from 0.75 Ω for MOF(Co) to 0.74 Ω for MOF(Co)‐SA1, and finally to 0.68 Ω for MOF(Co_4_Ni_1_)‐SA1, which indicates lower ohmic obstruction in the electron transfer and facilitated reaction kinetics.

The double‐layer capacitance (C_dl_) is approximately proportional to the electrochemically active surface area (ECSA), and its value can also be used to evaluate catalyst activity (Figure S36). As shown in Figure S36d, compared to MOF(Co) (2.13 mF cm^−2^), the C_dl_ of MOF(Co)‐SA1 increases to 3.53 mF cm^−2^. MOF(Co_4_Ni_1_)‐SA1 exhibits the largest C_dl_ (6.29 mF cm^−2^), indicating that modulating ligand defects via the SA strategy to open the MOF crystal structure and incorporating Ni to tune the Co metal centers exposes more active sites. The ECSA‐normalized current density plot (Figure S37) reveals that MOF(Co_4_Ni_1_)‐SA1 exhibits significantly higher specific activity compared to MOF(Co) and MOF(Co)‐SA1, highlighting its superior intrinsic electrocatalytic performance. Subsequently, the catalytic stability of MOF(Co_4_Ni_1_)‐SA1 is discussed. Chronopotentiometry (CP) measurements at 100 mA cm^−2^ demonstrate the high catalytic stability of MOF(Co_4_Ni_1_)‐SA1, with the overpotential increasing by only 20 mV after 100 h, outperforming that of commercial RuO_2_ (Figure 7d). Figure S38 shows the HRTEM images of MOF (Co_4_Ni_1_)‐SA1 after a 100‐h stability test. No hexagonal sheets corresponding to CoOOH are observed in the post‐test sample. The lattice spacings visible in the images are attributed to CoOOH, CoNiOOH, and Co_2_(Ni)O_4_, while no lattice fringes belonging to CoO_2_ are detected. This absence is due to the highly unstable nature of CoO_2_, which validates the proposed OER process [42]. The inset shows a slight decrease in R_ct_ after 100 h (0.52 Ω, Error% % = 3.00%), suggesting that the reconstructed catalyst facilitates charge transfer more effectively. Conversely, an increase in Rs (1.20 Ω, Error% 0.25%) suggests increased solution resistance due to OH^−^ consumption in the electrolyte, which likely contributes to the minor OER performance decay. Figure 7e compares the OER performance of MOF(Co_4_Ni_1_)‐SA1 with other advanced catalysts, highlighting its superior performance [43, 44, 45, 46, 47, 48, 49, 50, 51].

To emphasize the practical significance of the MOF(Co_4_Ni_1_)‐SA1 reconstruction activity for alkaline water oxidation, the overall water‐splitting cell is evaluated, which employs commercial Pt/C as the cathode for the hydrogen evolution reaction (HER) (HER performance shown in Figure S39, ƞ_10_ = 24.6 mV) and MOF(Co_4_Ni_1_)‐SA1 as the anode. The polarization curve of this cell shows water splitting voltages of 1.51, 1.59, and 1.69 V at 10, 100, and 500 mA cm^−2^, respectively (Figure 7f). This is consistent with the water splitting voltage estimates at 10 mA cm^−2^ from the combined HER and OER polarization curves obtained in the three‐electrode system (Figure 7g). The CP test is conducted at 100 mA cm^−2^ for 120 h, during which the cell voltage increases by 60 mV, demonstrating the practical utility of MOF(Co_4_Ni_1_)‐SA1 (Figure 7h). The inset reveals that this Voltage increase is attributed to the degradation of the commercial Pt/C cathode after the stability test. In contrast, MOF(Co_4_Ni_1_)‐SA1 maintains stability comparable to that observed in the OER performance tests (three electrode system). Figure 7i demonstrates the successful powering of the water splitting cell using the asymmetric supercapacitor, highlighting the practical utility potential.

## 3 Conclusion

In summary, rationally modulating both the crystal structure and electronic structure of MOF(Co) during electrodeposition promotes its exceptional energy storage and OER performance. By utilizing the competing ligand SA to effectively modulate the MOF(Co) crystal structure, MOF(Co)‐SA1 with a nanocrystalline fragment‐amorphous structure is prepared. The transition from an ordered to a disordered structure, observed via HAADF‐STEM, signifies the effective modulation of MOF(Co) by the SA ligand. DFT calculations are performed on both MOF(Co) and MOF(Co)‐SA while maintaining periodicity. The open crystal structure facilitates a reduced reaction energy barrier for the conversion of the MOF(Co) material to CoOOH. The electronic structure of the Co metal center is modulated by introducing a second metal center of Ni. The XAS plots reveal that Ni incorporation effectively induces electron transfer from Co to Ni, resulting in a higher oxidation state for Co. DFT calculations on the constructed MOF(CoNi)‐SA model similarly reveal the formation of high‐valent Co metal centers while maintaining the low reaction energy barrier characteristic of MOF(Co)‐SA.

Consequently, nanocrystalline fragment‐amorphous MOF(Co_4_Ni_1_)‐SA1 is constructed by rapid electrodeposition, featuring metal centers (Co and Ni) with high oxidation states (between +2 and +3). In the energy storage (redox) region, MOF(CoNi)‐SA exhibits outstanding electrochemical performance, demonstrating excellent rate capability (917 F g^−1^@ 1 A g^−1^, with 71% retention @ 50 A g^−1^) and cycling stability (95.6% capacitance retention after 10 000 cycles @ 50 A g^−1^). The fabricated asymmetric quasi‐solid‐state (aqueous) supercapacitor achieves a superior energy density of 37.4 (41.9) Wh kg^−1^ @ 850 W kg^−1^, confirming the potential of materials for energy storage. Furthermore, during the electrochemical surface reconstruction process, the high‐valent Co metal centers in MOF(Co_4_Ni_1_)‐SA1 not only accelerate the formation of CoNiOOH but also induce a decrease in the oxidation potential of Co^4+^ formation. This optimizes its OER performance (η_10_ = 259 mV, η_500_ = 318 mV). Therefore, utilizing electrodeposition to rationally tune the MOF crystal and electronic structure accelerates the reconstruction of the true active phase, providing a large‐scale, efficient, and rapid approach for preparing novel materials for energy storage and conversion applications.

## 4 Experimental Section

### 4.1 Electrodeposition of MOF(Co)‐*t* (*t* is the electrodeposition time) Material

All chemicals were of analytical grade and used without further purification. Firstly, the Ni Foam (NiF) measuring 1 cm × 1.5 cm was subjected to ultrasonic cleaning with acetone and distilled water for 1 min each, sequentially. The NiF was then immersed in a solution of HCl (37 wt.% %) for 30 s to remove the oxide layer and surface impurities. A standard three‐electrode glass cell was utilized at room temperature for the electrodeposition process of MOF(Co)‐74 on the NiF. The NiF served as the working electrode, the silver chloride electrode (Ag/AgCl) was used as the reference electrode, and a Pt/Ti sheet was employed as the counter electrode. The electrolyte solution was prepared by dissolving 48 mm Co(NO_3_)_2_·6H_2_O and 24 mm H_4_dobdc in 100 mL of a ternary solvent system comprising equal volumes of DMF, ethanol, and deionized water. A pre‐cleaned NiF substrate was immersed as the working electrode, and potentiostatic deposition was performed at −1.57 V vs. Ag/AgCl with controlled durations ranging from 300 to 1200 s. The synthesized samples underwent sequential purification through deionized water rinsing followed by thermal treatment at 50°C for 4 h.

The mass loading of MOF samples electrodeposited was quantified through gravimetric analysis of nickel foam substrates before and after deposition. Progressive mass increases of MOF(Co)‐*t* (*t* = 300, 600, 900, and 1200s) were recorded as 0.3 (300s), 0.7 (600s), 1.7 (900s), and 3.1 mg (1200s).

### 4.2 Electrodeposition of MOF(Co)‐SA*x* (*x* is the ratio of SA and H_4_dobdc) Material

Following the established electrodeposition protocol for MOF(Co) synthesis, identical deposition potential and duration are maintained. The ligand of SA was introduced into the electrolyte at varying SA/H_4_dobdc molar ratios while keeping the total ligand (SA + H_4_dobdc) concentration constant at 24 mm. The electrodeposited samples (named as MOF(Co)‐SA*x*, *x* = 0.5, 1, 2) underwent thorough rinsing with deionized water followed by drying at 50°C. The mass of MOF(Co)‐SA*x* (*x* = 0.5, 1, 2) was recorded as 1.7, 1.6, and 1.6 mg, respectively.

### 4.3 Preparation of MOF(Co*_y_* Ni*_z_*)‐SA1 (*y* and *z* are the ratios of cobalt and nickel) Material

Under MOF(Co)‐SA1 electrodeposition conditions, NiCl_2_·6H_2_O was introduced into the plating bath with precise control of total metal ion concentration (Co^2+^ + Ni^2+^ = 48 mm). Three distinct Co: Ni molar ratios (6:1, 4:1, 2:1) were achieved by adjusting *y* (Co^2+^) and *z* (Ni^2+^) concentrations accordingly. The electrodeposited specimens underwent sequential purification through deionized water rinsing and thermal stabilization at 50°C, yielding the final products designated as MOF(Co*_y_* Ni*_z_*)‐SA1. The mass of MOF(Co*_y_* Ni*_z_*)‐SA1 was all recorded as 1.6 mg.

## Funding

This research was supported by the Korea Institute for Advancement of Technology (KIAT), funded by the Ministry of Trade, Industry & Energy (MOTIE, Korea). (P0026243). This work was supported by the National Research Foundation of Korea (NRF) grant funded by the Korea government (MSIT) (No. RS‐2023‐00260527). This work was supported by the Natural Science Foundations of Fujian (2024J01922).

## Conflicts of Interest

The authors declare no conflicts of interest.

## Supporting information




**Supporting File**: advs73430‐sup‐0001‐SuppMat.docx.

## Data Availability

The data that support the findings of this study are available from the corresponding author upon reasonable request.
