# Supplementary material for: Rapid Electrodeposition of Defect‐Tuned MOF Nanoarchitectures: Synergistic Unlocking Electrochemical Performance via Dual Modulation of Morphology and Electronic Structure
Source: Adv Sci (Weinh). 2025 Dec 26;13(13):e20784. doi: 10.1002/advs.202520784 (PMC12955893; doi:10.1002/advs.202520784)
Supplement: Supplementary file 1 — Supporting File: advs73430‐sup‐0001‐SuppMat.docx. [file ADVS-13-e20784-s001.docx]

Supporting Information

Rapid Electrodeposition of Defect-Tuned MOF Nanoarchitectures: Synergistic Unlocking Electrochemical Performance via Dual Modulation of Morphology and Electronic Structure

*Qing Wang, Zehui Yu, Sanghwa Yoon*, Zitao Yang*, and Bongyoung Yoo**

*The “*” is marked as corresponding authors*

Q. Wang, Prof. Dr. S. Yoon, Prof. Dr. B. Yoo

Department of Materials Science and Chemical Engineering,

Hanyang University,

Ansan 15588, Republic of Korea

E-mail: indada@naver.com; [byyoo@hanyang.ac.kr](mailto:byyoo@hanyang.ac.kr)

Prof. Dr. Z. Yang

Department of Ecology and Resource Engineering,

Fujian Provincial Key Laboratory of Eco-Industrial Green Technology, Wuyi University

Fujian 354300, Republic of China

E-mail: tzy1962@126.com

Z. Yu

School of Chemistry and Chemical Engineering,

Nanjing University,

Nanjing, Jiangsu 210023, Republic of China

# Synthetic details

**1. Preparation of electrocatalysts**

**1.1 Synthesis of reduced graphene oxide (rGO) anode electrodes on the nickel foam.**

The anode electrode of the supercapacitor device was prepared using commercial rGO as the active material. A homogeneous slurry was prepared by mixing 80 wt% active materials, 10 wt% super P, and 10 wt% polytetrafluoroethylene (PTFE) under ultrasonication. The working electrodes were fabricated by homogeneously rolling as a sheet, then coating the slurry on nickel foam and drying at 60 °C for 12 h.

**1.2. Preparation of Commercial RuO_2_ electrode**

10 mg of commercial RuO_2_ dispersed in 500 μL of isopropanol and 15 μL of 0.5 wt% Nafion solution. The prepared slurry was uniformly coated onto the cleaned nickel foam, a certain pressure was applied, and it was placed in an oven to dry at 50°C. The weight density of RuO_2_ on NF is 1 mg·cm^−2^.

**1.3. Preparation of Commercial Pt/C electrode**

10 mg of commercial Pt/C dispersed in 500 μL of isopropanol and 15 μL of 0.5 wt% Nafion solution. The prepared slurry was uniformly coated onto the cleaned nickel foam, a certain pressure was applied, and it was placed in an oven to dry at 50°C. The weight density of Pt/C on NF is 1 mg·cm^−2^.

# Experimental section

**2. Materials characterization**

The morphology and microstructure of the materials were observed using scanning electron microscopy (SEM), high-resolution transmission electron microscopy (HRTEM), and high-angle annular dark-field scanning transmission electron microscopy (HAADF-STEM). The microstructure was analyzed using X-ray diffraction (XRD), X-ray photoelectron spectroscopy (XPS), X-ray absorption fine structure (XAFS), and Fourier transform infrared spectroscopy (FTIR). The redox was evaluated during the charging-discharging process by in situ Raman measurement.

The synthesized materials underwent comprehensive characterization through multiple analytical techniques. Microstructural evaluation employed field emission scanning electron microscopy (FE-SEM, TESCAN MIRA3) coupled with elemental mapping via energy-dispersive X-ray spectroscopy (EDS). High-resolution transmission electron microscopy (HRTEM) investigations utilized a JEOL JEM-2100F system, and aberration-corrected high-angle annular dark-field scanning transmission electron microscopy (AC-HAADF-STEM). STEM imaging was conducted on the JEM-2100F equipped with a Cs corrector. Crystalline structure determination was performed using X-ray diffraction (XRD, Rigaku SmartLab) with Cu Kα radiation (λ=0.15406 nm) under operational parameters of 40 kV and 40 mA, employing a 1° min⁻^1^ scanning rate. Surface chemical analysis was conducted through X-ray photoelectron spectroscopy (XPS) on a VG SIENCTA R3000 instrument equipped with Al Kα excitation. Molecular bonding characteristics were investigated by Fourier transform infrared spectroscopy (FTIR, Thermo Nicolet5700) across the mid-infrared spectral range.

The details for XPS are shown below:

For the XPS fitting, the Shirley background method was applied to account for inelastic scattering of photoelectrons. To correct for charging effects, the C 1s peak of adventitious carbon was set at a binding energy of 284.78 eV. This serves as an internal reference to align all other spectral features accurately. The peak profiles were fitted using a Lorentzian-Gaussian function with a fixed ratio of 80%.

The details for XAFS are shown below:

Data reduction, data analysis, and EXAFS fitting were performed and analyzed with the Athena and Artemis programs of the Demeter data analysis packages ^[1]^ that utilizes the FEFF6 to fit the EXAFS data^[2]^. The energy calibration of the sample was conducted through a standard Co foil, which, as a reference, was simultaneously measured. A linear function was subtracted from the pre-edge region, then the edge jump was normalized using Athena software. The χ(k) data were isolated by subtracting a smooth, third-order polynomial approximating the absorption background of an isolated atom. The k3-weighted χ(k) data were Fourier transformed after applying a Hanning window function (Δk = 1.0). For EXAFS modeling, the global amplitude EXAFS (CN, R, σ 2 and ΔE0) were obtained by nonlinear fitting, with least-squares refinement, of the EXAFS equation to the Fourier-transformed data in R-space, using Artemis software, EXAFS of the Co foil was fitted and the obtained amplitude reduction factor S0 2 value (0.738) was set in the EXAFS analysis to determine the coordination numbers (CNs) in sample.

The In-situ Raman Measurement:

In-situ Raman measurements were conducted using a Horiba LabRAM HR Evol confocal Raman microscope equipped with a 633 nm laser. A spectroelectrochemical cell was employed for the in-situ characterization, featuring a MOF (Co_4_Ni_1_)-SA1 working electrode, a Pt sheet counter electrode, and an Hg/HgO reference electrode. The measurements were taken at set potential intervals. Charging was performed at a current density of 1 A g^−1^ from 0 V to 0.5 V, with spectra collected every 0.1 V. Subsequently, discharging was carried out at −1 A g^−1^ from 0.5 V back to 0 V, with spectra acquired at intervals of −0.1 V.

**3. Density Functional Theory (DFT) calculations**

The bulk structure of the conventional MOF(Co) cell was first established. The MOF(Co)-SA model was obtained by substituting one H_4_dobdc ligand with an SA ligand in MOF(Co). The MOF(CoNi)-SA structure was then generated by replacing one Co atom with Ni in MOF74-Co-SA. All DFT calculations were carried out using the Vienna Ab initio Simulation Package (VASP) with the projector augmented wave (PAW) method. ^[3-4]^ The exchange–correlation functional was treated with the Perdew–Burke–Ernzerhof (PBE) functional together with the DFT-D3 correction.^[5-6]^ A plane-wave cut-off energy of 450 eV was applied. Lattice optimizations were performed with a Monkhorst–Pack k-point grid of 1×1×4 for Brillouin Zone sampling. ^[7]^ The self-consistent field calculations converged to an energy threshold of 10^−5^ eV. The equilibrium geometries and lattice constants were optimized until the residual stress on each atom was below 0.02 eV Å^−1^. Spin polarization was included to describe the magnetic properties of the models. The isosurface value of the charge density difference was set at 0.02 Å^−3^. Band structures and density of states were calculated using the GGA-PBE functional and further processed with the VASPKIT interface. ^[8]^

**4. Electrochemical measurements**

All reported electrochemical performance data are based specifically on the mass of the active material, not the total electrode mass (including Ni foam).

**4.1. Energy storage**

Electrochemical characterization was performed at ambient temperature using a Versa STAT3 workstation (AMETEK) with a three-electrode system in 2 M KOH electrolyte, where Hg/HgO served as the reference electrode and Pt/Ti as the counter electrode. EIS measurements were conducted under open-circuit conditions over a frequency range of 10^5^ to 0.01 Hz with 10 mV amplitude at open circuit potential. Cyclic voltammetry (CV) tests employed scan rates from 5 to 100 mV s^−1^ within the 0-0.6 V vs Hg/HgO potential window. Galvanostatic charge-discharge (GCD) measurements utilized current densities ranging from 1 to 50 A g^−1^ across a 0-0.5 V operational window.

Asymmetric supercapacitors (ASCs) were fabricated by pairing the MOF(Co_4_Ni_1_)-SA1 cathode with commercial rGO anode in both aqueous (KOH) and quasi-solid-state (PVA/KOH) configurations, followed by comprehensive electrochemical evaluation.

In a three-electrode system, the [cyclic voltammetry](https://www.sciencedirect.com/topics/chemistry/cyclic-voltammetry) (CV) was carried out within the potential range between 0 and 0.6V. Electrochemical impedance spectra were recorded over the frequency range from 100 kHz to 0.01 Hz. The charge-discharge measurements were performed over a voltage range from 0 to 0.5 V at room temperature.

To investigate the kinetic processes occurring at the electrode, the linear dependence of peak current (i) on scan rate (v) in cyclic voltammetry (CV) curves is analyzed using the power-law relationship $i=a v^{b}$, where a and b are constants, where *i* (A cm^−2^) denotes the peak current density, and *v* (mV s^−1^) represents the scan rate. The *b*-value, derived from the slope of the serves to distinguish dominant charge storage mechanisms: a slope approaching 1 indicates capacitive-dominated behavior, while a slope near 0.5 suggests diffusion-controlled battery-like processes^[9]^.

To further quantify the contributions of diffusion-controlled and capacitive-controlled processes, CV curves collected at scan rates ranging from 5 to 100 mV s⁻^1^ are analyzed using the following equation^[10]^:

$i=k_{1}v+k_{2}v^{1/2}$ (1)

 The original equation can be mathematically reformulated to simplify analysis.

$i/v^{1/2}=k_{1}v^{1/2}+k_{2}$ (2)

The specific capacitance *C_s_* (F g^−1^) was calculated from the galvanostatic discharge process according to the following equation [1]:

$C_{s}=\frac{I\times\Delta t}{\Delta V\times m}$(3)

$C_{\mathrm{sa}}=\frac{I\times\Delta t}{\Delta V\times S}$(4)

Where *I* represented the discharge current, $\Delta t$ corresponded to the discharge time, $\Delta V$ was the potential window, *m* was the mass of active materials, and *S* was the geometrical area of the electrode materials.

The electrochemical performances of MOF(Co_4_Ni_1_)-SA1 were further evaluated on aqueous (KOH) and quasi-solid-state (PVA/KOH) ASC devices. The ASC was assembled by MOF(Co_4_Ni_1_)-SA1 and commercial rGO as the cathode and anode electrode, respectively.

The mass ratio of the cathode and anode electrode was obtained using the charge balance theory (Q^+^=Q^−^) [2]：

$\frac{m_{+}}{m_{-}}=\frac{C_{s-}\times\Delta V_{-}}{C_{s+}\times\Delta V_{+}}$ (5)

Where m was the mass of the electrode (g), $C_{s-}$ and $C_{s+}$ were the specific capacitance (F g^−1^) at 10 mV s^−1^, and ΔV = potential window (V). The mass ratio of MOF(Co4Ni1)-SA1 and rGO was 1:2.3 in the fabricated ASC device. In this work, the masses of the cathode and anode were taken as about 1.6 mg and 3.7 mg, respectively.

The energy density and power density of the ASC device are calculated via equations (6) and (7) [2]:

$E_{mass}=\frac{1}{2\times3.6}\times C_{s}\times\Delta V^{2}$ (6)

$P=3600 E/t$ (7)

where *E* is the energy density (Wh kg^–1^), *C_s_* denotes the specific capacitance (F g^−1^), *ΔV* represents the voltage window (V), *P* is the power density (W kg ^–1^), and *t* denotes discharge time (s).

**4.2. OER and water splitting performance**

**4.2.1 Calibration of the reference electrode**

The Hg/HgO (1.0 M KOH) reference electrode was calibrated against the reversible hydrogen electrode (RHE). The calibration experiment was performed in an H_2_-saturated electrolyte (1M KOH) using Pt wires as both the working and counter electrode. Cyclic voltammetry was conducted at a scan rate of 1 mV s^−1^. The RHE potential was subsequently determined by averaging the potentials at which the anodic and cathodic scans crossed the zero-current line for the hydrogen electrode reaction^[11]^, as shown in **Figure S33**. According to the results shown in Figure S33, E(RHE) = E (Hg/HgO) + 0.926 V.

Furthermore, to validate the reliability of our Hg/HgO (1 M KOH, 0.098 V vs. SHE) reference electrode, we performed another test by connecting it to a brand-new Ag/AgCl (saturated KCl, 0.197 V vs. SHE) reference electrode in 1 M KOH electrolyte and measuring the potential difference between them with a multimeter. The measured voltage difference was within 10 mV of the theoretically expected value, confirming that our Hg/HgO electrode was functioning within an acceptable standard range. Combined the two results that the standard potential of the Hg/HgO (1 M KOH) electrode was taken as 0.098 V vs. SHE in our case, and for comparison to other papers, the recorded potentials were subsequently converted to the RHE scale using the equation below:

E (vs. RHE) = E (vs. Hg/HgO) + 0.0591 × pH + E (Hg/HgO, 0.098 V vs. SHE) (8)

The pH value of a 1M KOH solution is 14.

Therefore, the E (vs. RHE) = E (vs. Hg/HgO)+ 0.9254 (Theoretical)

**4.2.2 HER and OER electrocatalytic measurement**

The HER and OER performances were tested by using a typical three-electrode system in 1 M KOH using Versa STAT3 AMETEK Co., where the synthesized samples, graphite foil, and Hg/HgO (E (vs. RHE) = E (vs. Hg/HgO) + 0.0591 × pH + E(Hg/HgO)) were used as work, counter, and reference electrode, respectively. The linear sweep voltammetry (LSV) measurements of HER and OER at a 5 mV s^−1^ scan rate with 80% *i*R-correction were severely. The *i*R-Corrected was used by the equation: *i*R-Corrected value = E (vs. RHE) − *i* × R_s_ (*i* – current, R_s_ – equivalent series resistance). The kinetic mechanism of the HER and OER reactions was estimated by the Tafel electrochemical equation. The EIS was recorded to evaluate the R_ct_ from 1 × 10^5^ to 10^−1^ Hz at −1 V (vs. Hg/HgO) for HER and 0.7 V (vs. Hg/HgO) for OER, respectively. The C_dl_ data were obtained by cyclic voltammetry at a potential range of 0.7 to 0.8 V (vs. RHE) with different scan rates (10 -100 mV s^−1^).

The value of ECSA was calculated from the equation:

$ECSA= \frac{C_{dl}}{C_{s}\times S}$ (9)

(The Cs value in 1 M KOH is 0.04 mF cm^−2^, and S represents the actual geometric area of the working electrode).

The overall water splitting performance was evaluated using a two-electrode system with Commercial Pt/C as the cathode and MOF(Co_4_Ni_1_)-SA1 as the anode. LSV was measured in 1 M KOH using a VersaSTAT3 (AMETEK Co.) within a voltage range of 1 to 2.5 V at a constant scan rate of 5 mV s^−1^, with *i*R correction applied.

# Supplementary data

**Figure and Tables**


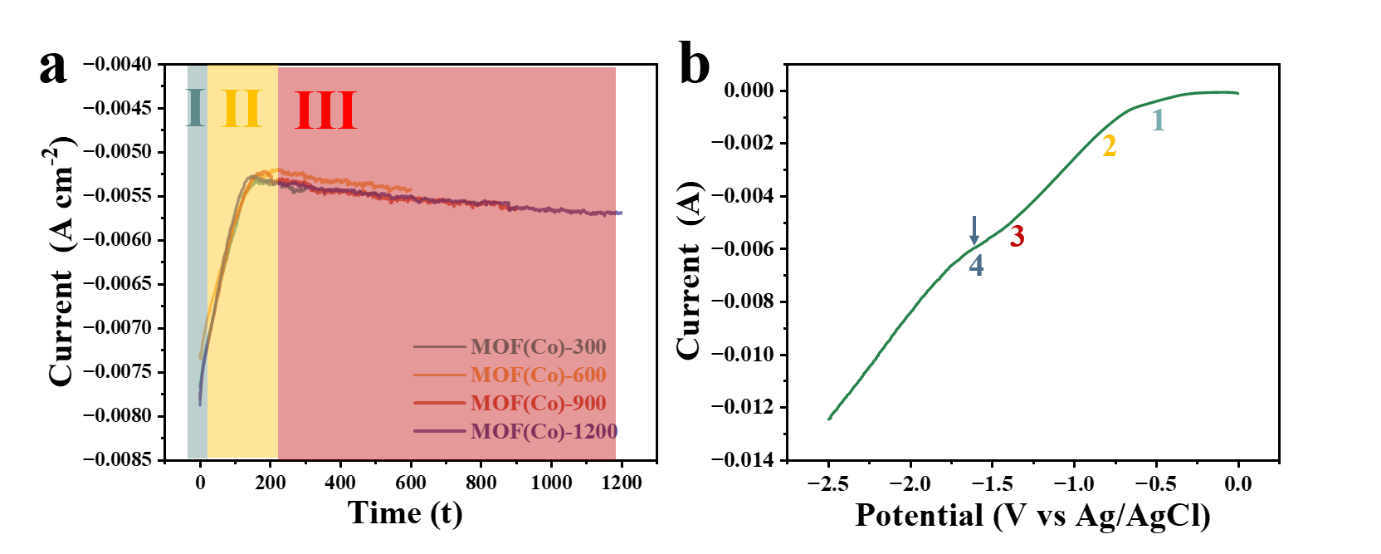


**Figure S1.** (a) The chronoamperometric curve for electrodeposition of MOF(Co)-*t* samples. (b) linear sweep voltammetry (LSV) of Ni foam (NiF) in the electrodeposition electrolyte solution.

The reactions in the LSV curve below:

$Co^{2+}+{2e}^{-}\leftrightarrow Co^{0}$[1]

$NO_{3}^{-}+H_{2}O+2e^{-}\to NO_{2}^{-}+2OH^{-}$[2]

$2OH^{-}+H_{4}dobdc+2e^{-}\leftrightarrow dobdc^{4-}+2H_{2}O$ [3]

$2Co^{2+}+dobdc^{4-}\leftrightarrow Co_{2}\left[ dobdc \right]$[4]


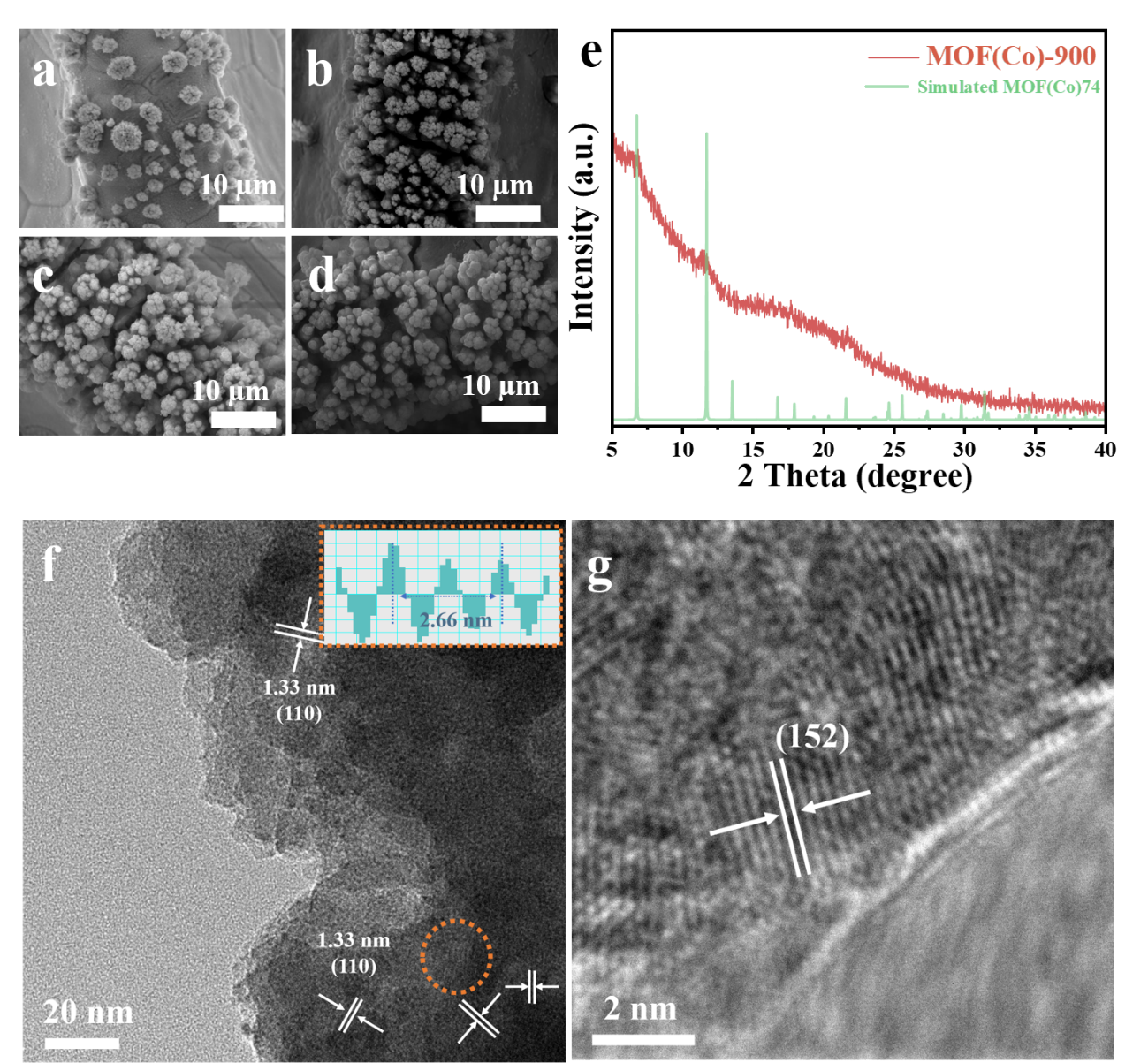


**Figure S2.** The SEM images of MOF(Co)-*t* samples. (a) MOF(Co)- 300. (b) MOF(Co)-600. (c) MOF(Co)-900. (d) MOF(Co)-1200. (e) The XRD spectra of MOF(Co)-900 samples. (f, g) The HRTEM images of MOF(Co)-900.

Figure S2a-S2d shows the electrodeposition morphologies of MOF(Co) samples as the electrodeposition time increased using H_4_dobdc as the organic linker. Initially, the deposited MOF particles form large aggregates that assemble into micrometer-scale cauliflower-like morphologies. With prolonged deposition time, the quantity of these cauliflower-shaped MOF structures gradually increases, accompanied by an expansion in particle size. Concurrently, the crystallinity of the material proves the MOF materials synthesis successful (Figure S2e, XRD spectra). The HRTEM images (Figure S2f, g) show the crystal structure of MOF(Co). While the bulk regions were generally too thick to resolve clear lattice fringes, ordered structures typical of MOFs were observable at thinner edges (Orange circle region), although the growth occurred along different directions (without the standardized shape for MOFs) in Figure S2f. The measured interplanar spacing along the line-marked region matches the (110) plane spacing of MOF-74. Figure S2g presents an enlarged view of another selected region, where distinct diffraction fringes corresponding to the (152) crystal plane are clearly visible. These results evidence that the MOF(Co)-900 with crystallization structure is electrodeposited successfully.

##
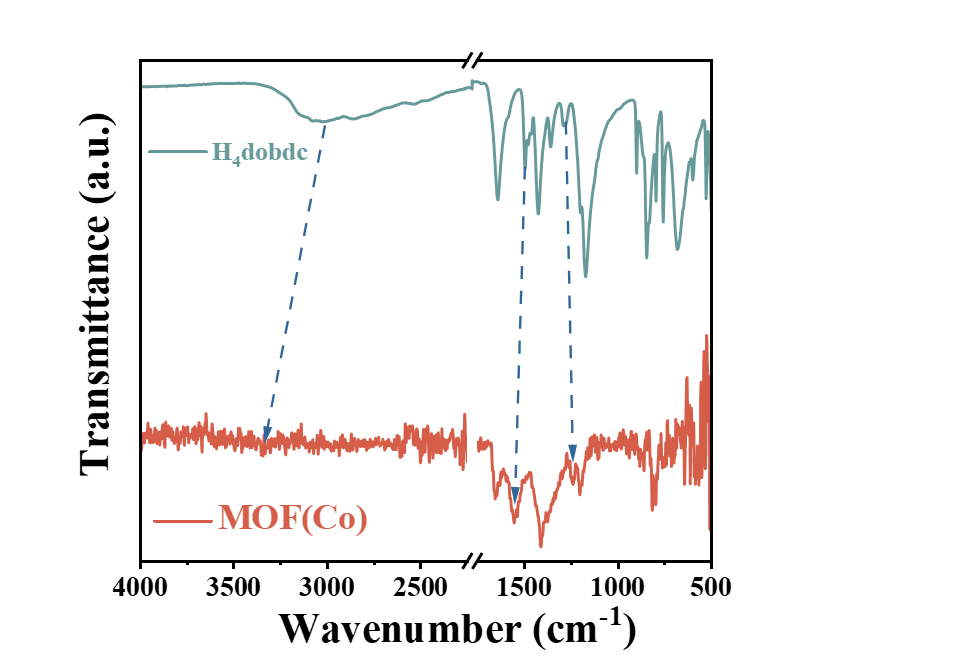


**Figure S3.** The FTIR spectra of H_4_dobdc and MOF(Co).


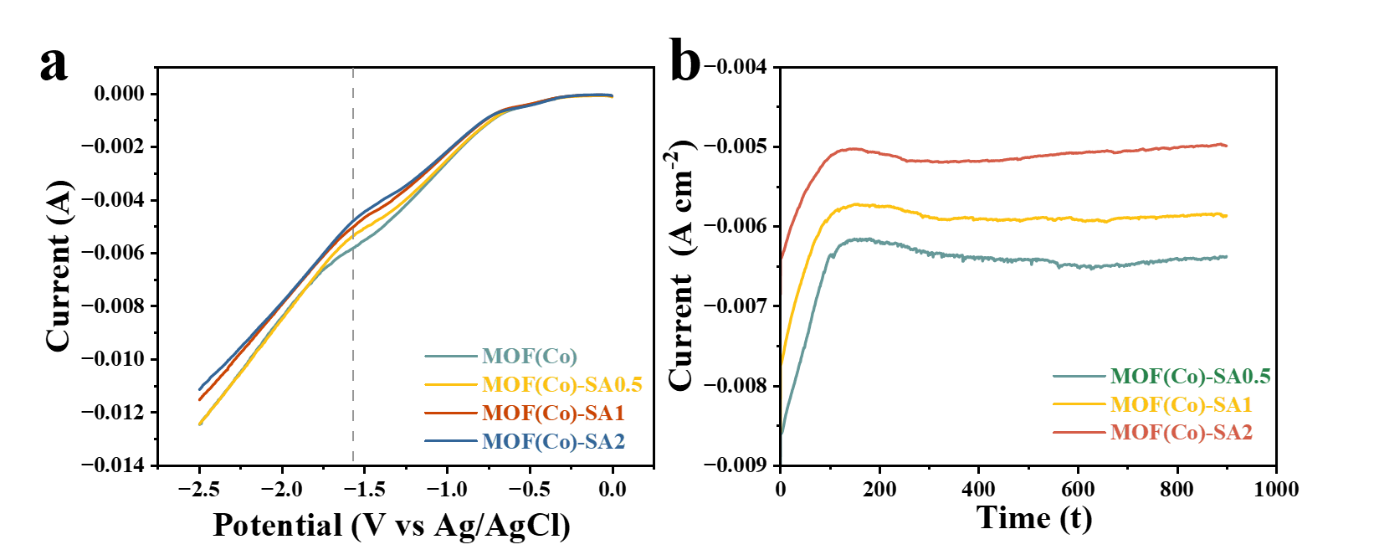


**Figure S4.** (a) linear sweep voltammetry (LSV) of Ni foam (NiF) in the electrodeposition electrolyte solution with SA. (b) The chronoamperometric curve for electrodeposition of MOF(Co)-SA*x* samples.

The current density of the reduction peak decreases for deprotonation with increasing SA concentration in LSV curves, which is because the competitive modulator SA possesses fewer hydroxyl and carboxyl groups per molecule than that of the original linker H_4_dobdc, leading to a cathodic shift of the deprotonation peak and reduced current density. This observation suggests that SA functions as a competitive ligand to generate structural defects and facilitates the coordination process. The correlation between current densities in chronoamperometric curves and LSV curves confirms the successful incorporation of SA as a competing ligand during MOF crystallization. (Figure S4b).

##
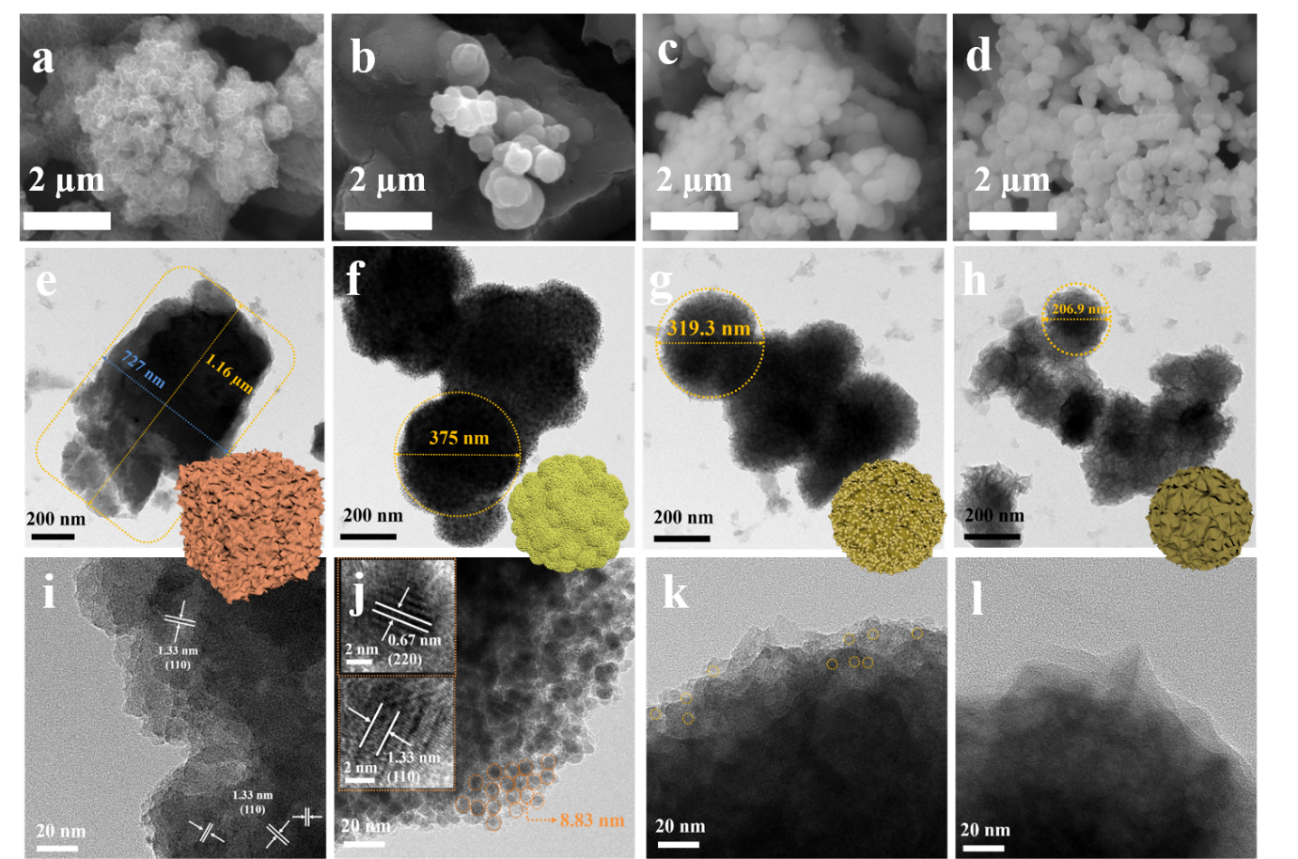


**Figure S5.** (a-d) SEM images of MOF(Co) and MOF(Co)-SA*x* (*x*=0.5, 1, and 2), respectively. (e-l) The HR-TEM images of MOF(Co), and MOF(Co)-SA*x* (*x*=0.5, 1, and 2), respectively.

The morphological evolution of MOF(Co)-SAx is investigated by SEM and TEM analysis. The morphological evolution of MOF(Co)-SAx is investigated by SEM and TEM analysis. Notably, SA modification induced a significant morphological transformation from micron-sized bulk particles (MOF(Co)) to spherical nanoparticles with an average diameter of 375 nm (MOF(Co)-SA1), as shown in Figure S5a, e, b, and f. Further increasing the SA content led to a progressive size reduction to 207 nm (Figure S5b, f, c, and g), demonstrating the effective role of SA in controlling particle dimensions^[12]^. Microstructural characterization is performed using HRTEM. The lattice spacing corresponding to 1.33 nm in the HRTEM of MOF(Co) corresponds to that of the (110) plane of MOF-74^[13]^. HRTEM image reveals well-defined 8.83 nm crystalline domains in MOF(Co)-SA0.5 (Figure S5j), which has a clear lattice spacing corresponding to the (220) and (110) plane^[14]^. Consistent with the LSV results, SA incorporation facilitates the deprotonation process and accelerates MOF formation. However, an increase in SA content correlates with a reduction in crystallization, implying the emergence of structural defects. At a 1:1 molar ratio of linker H4dobdc to competitive modulator SA, HRTEM imaging of MOF(Co)-SA1 reveals ill-defined nanocrystalline fragments (~2-3 nm) embedded within an amorphous matrix. This may be attributed to SA competing with H_4_dobdc for binding sites, leading to increased structural disorder, eventually forming amorphous architectures of MOF(Co)-SA2 (Figure S5l).

##
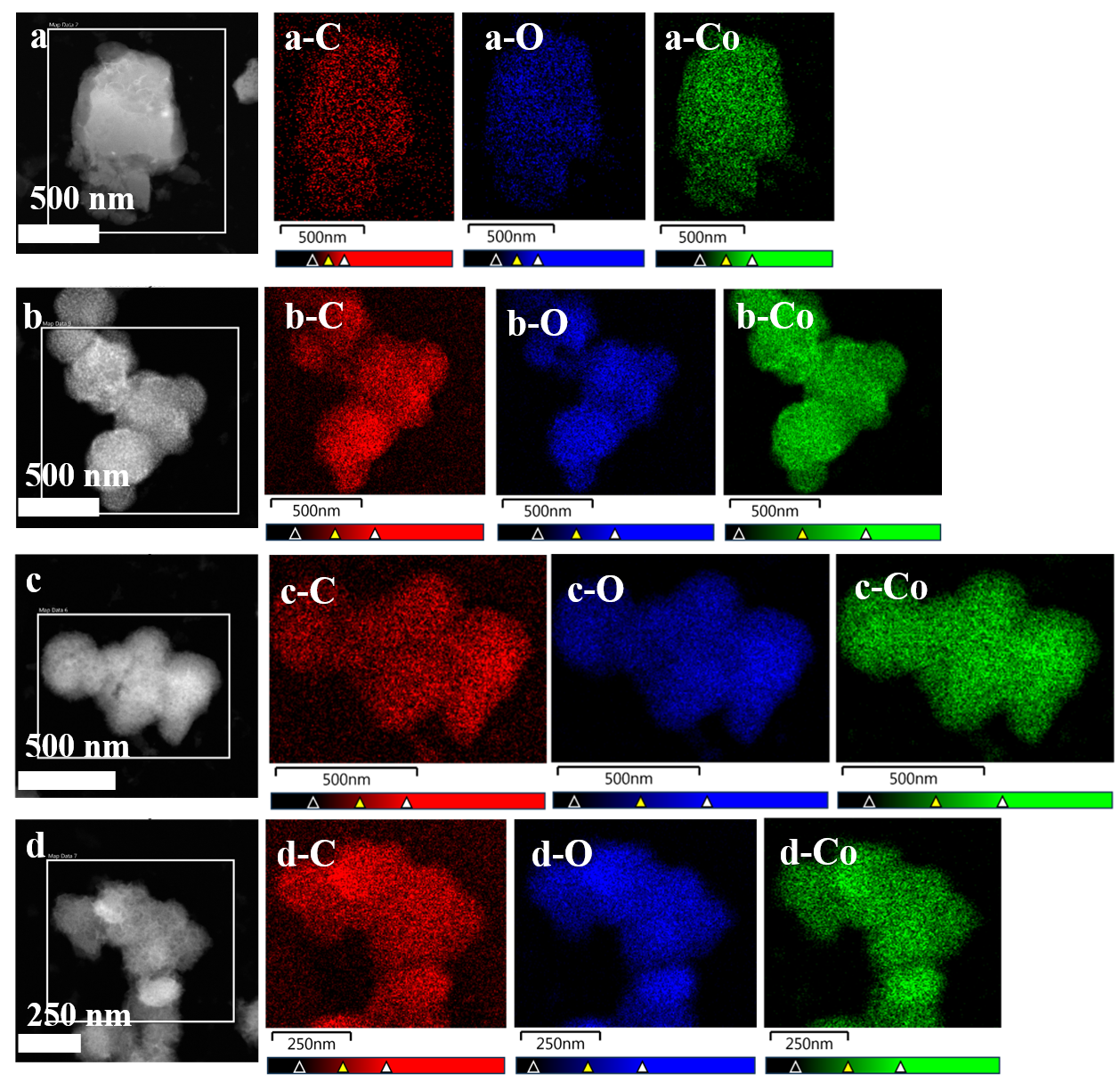


**Figure S6.** The mapping images of (a) MOF(Co) and (b-d) MOF(Co)-SA*x* (*x*=0.5, 1, 2), respectively.

**Table. S1** The elements content of MOF(Co) and MOF(Co)-SA*x* (*x*=0.5, 1, 2).

| **Samples** | **C** (At. %) | **O** (At. %) | **Co** (At. %) |
| --- | --- | --- | --- |
| **MOF(Co)** | 49.77 | 27.04 | 23.19 |
| **MOF(Co)-SA1** | 57.81 | 25.94 | 16.25 |
| **MOF(Co)-SA2** | 49.14 | 37.87 | 12.99 |
| **MOF(Co)-SA3** | 64.24 | 28.38 | 7.36 |


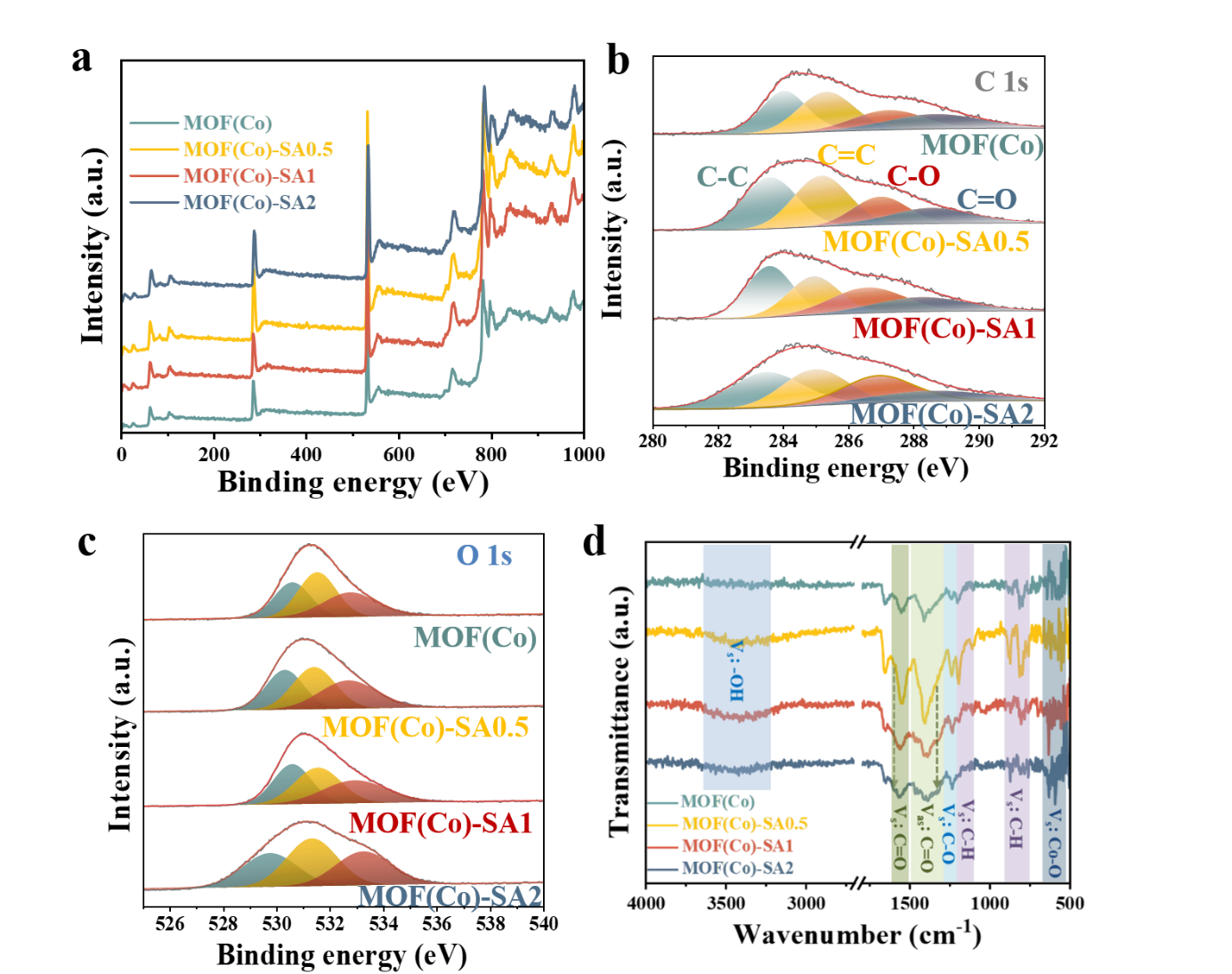


**Figure S7.** (a) The XPS spectra of MOF(Co) and MOF(Co)-SA*x*. High-resolution XPS of (b) C1s and (c) O1s. (d) FTIR spectra.

The Co 2p and C 1s peaks first shift to lower binding energy, suggesting the dominant influence of SA ligands on coordination electrons of Co (Figure S7). The O 1s spectrum consists of three components: lattice oxygen (M-O), coordinated oxygen, and surface-adsorbed oxygen (O_ads_)^[15]^.

**Table S2.** The content of Co-SA and Co-dobdc in the Co 2p spectra.

| **Samples** | Co-SA | Co-dobdc |
| --- | --- | --- |
| **MOF(Co)** | 0 | 100% |
| **MOF(Co)-SA0.5** | 31.1% | 67.9% |
| **MOF(Co)-SA1** | 44.5% | 55.5% |
| **MOF(Co)-SA2** | 67.1 | 32.9% |


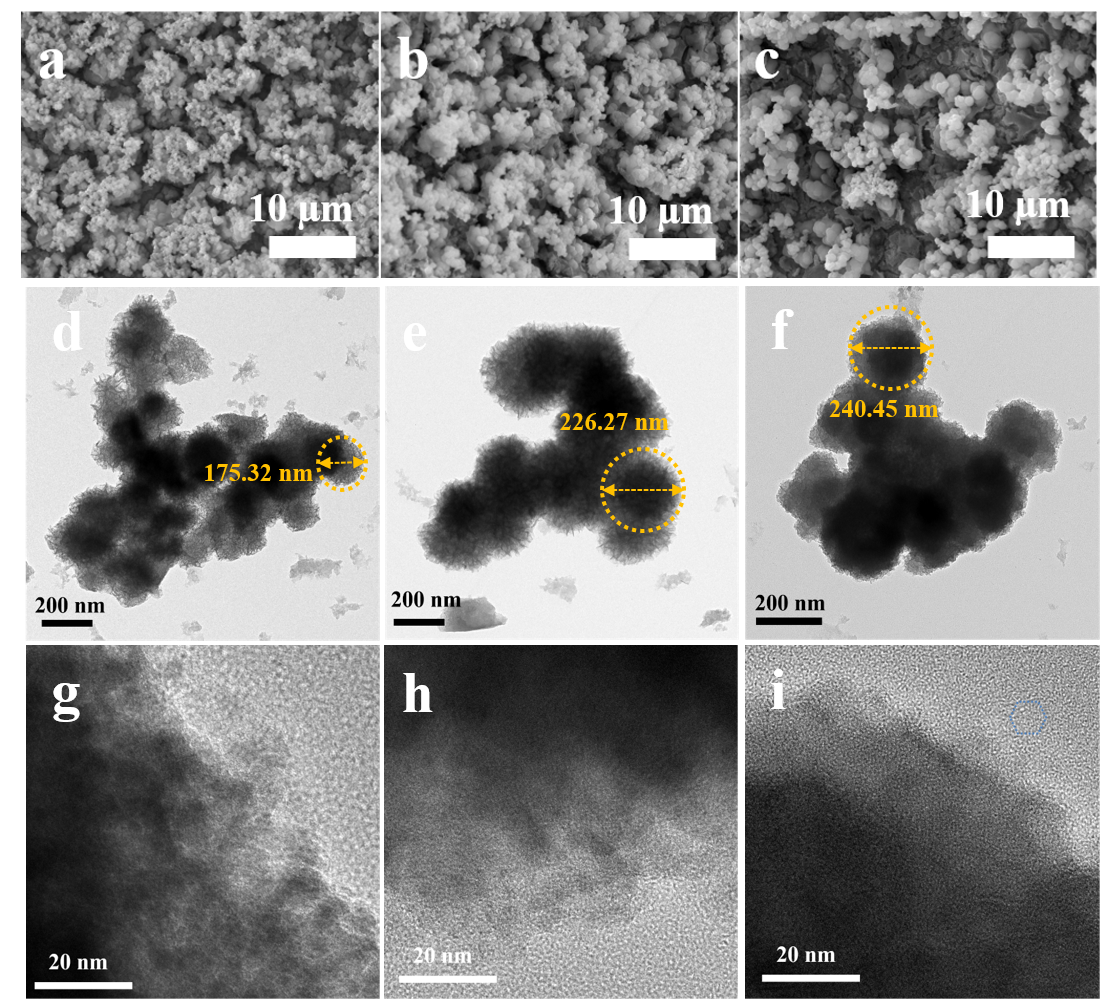


**Figure S8.** (a-c) The SEM images of MOF(Co*_y_*Ni*_z_*)-SA1 (d-f). The TEM images of MOF(Co*_y_*Ni*_z_*)-SA1. (g-i)The HR-TEM images of MOF(Co*_y_*Ni*_z_*)-SA1.

##
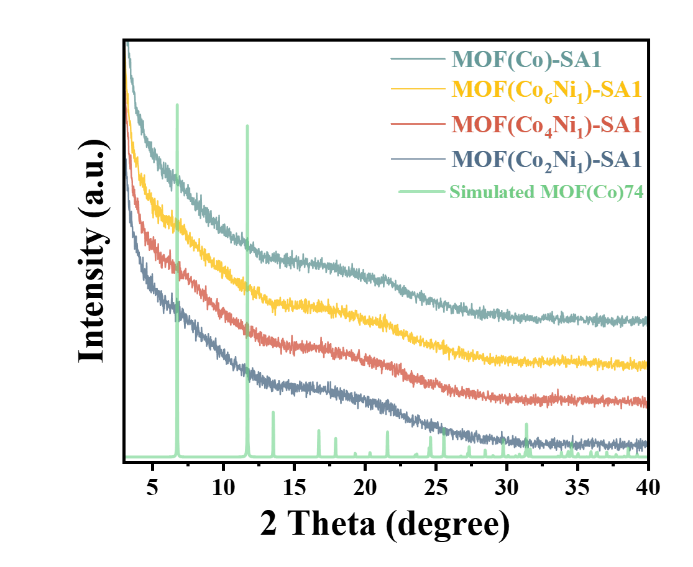


**Figure S9.** XRD spectra of MOF(Co)-SA1 and MOF(Co*_y_*Ni*_z_*)-SA1 (*y:z*=6:1, 4:1, 2:1) samples.


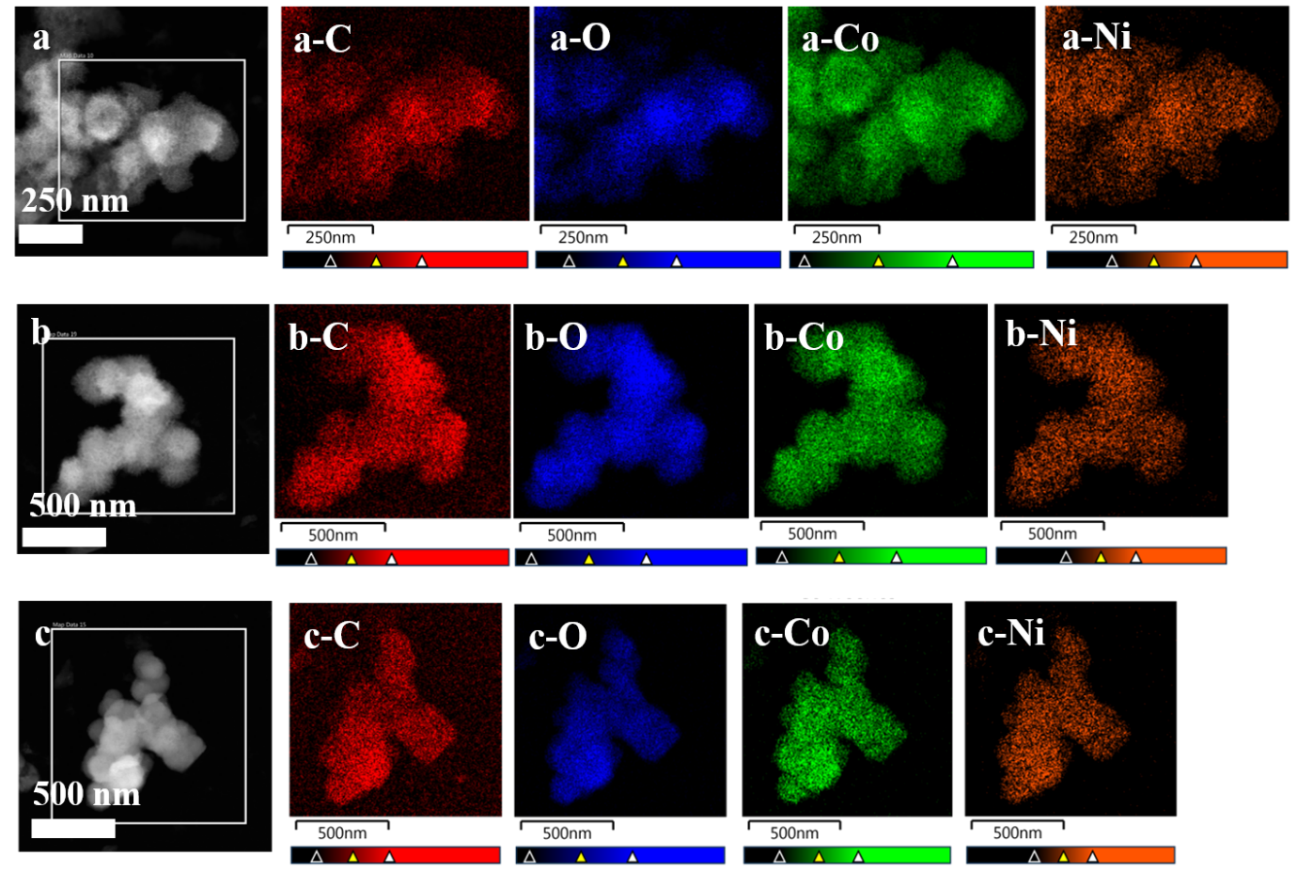


**Figure S10.** The mapping images of MOF(Co*_y_*Ni*_z_*)-SA1 samples.

**Table. S3.** The element content of MOF(Co*_y_*Ni*_z_*)-SA1 samples.

| **Samples** | **C** (At.%) | **O** (At.%) | | **Co** (At.%) | **Ni** (At.%) |
| --- | --- | --- | --- | --- | --- |
| **MOF(Co_6_Ni_1_)-SA1**  MOF(Co_0.86_Ni_0.14_)-SA1 | 44.95 | 41.46 | 11.76 | | 1.83 |
| **MOF(Co_4_Ni_1_)-SA1**  MOF(Co_0.79_Ni_0.21_)-SA1 | 50.06 | 38.36 | 9.15 | | 2.44 |
| **MOF(Co_2_Ni_1_)-SA1**  MOF(Co_0.66_Ni_0.34_)-SA1 | 59.89 | 32.56 | 4.99 | | 2.56 |


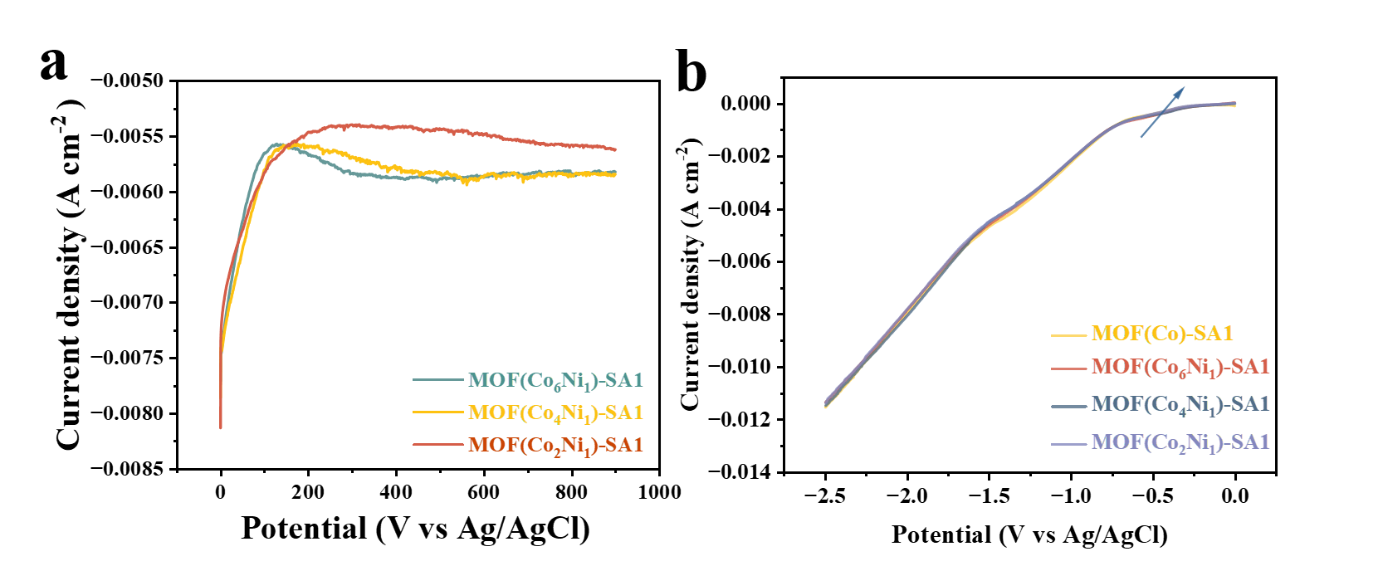


**Figure S11.** (a) The chronoamperometric curve for electrodeposition of MOF(Co_y_Ni_z_)-SA1 samples. (b) LSV curves of NiF in the electrodeposition electrolyte solution with SA and NiCl_2_·6H_2_O.


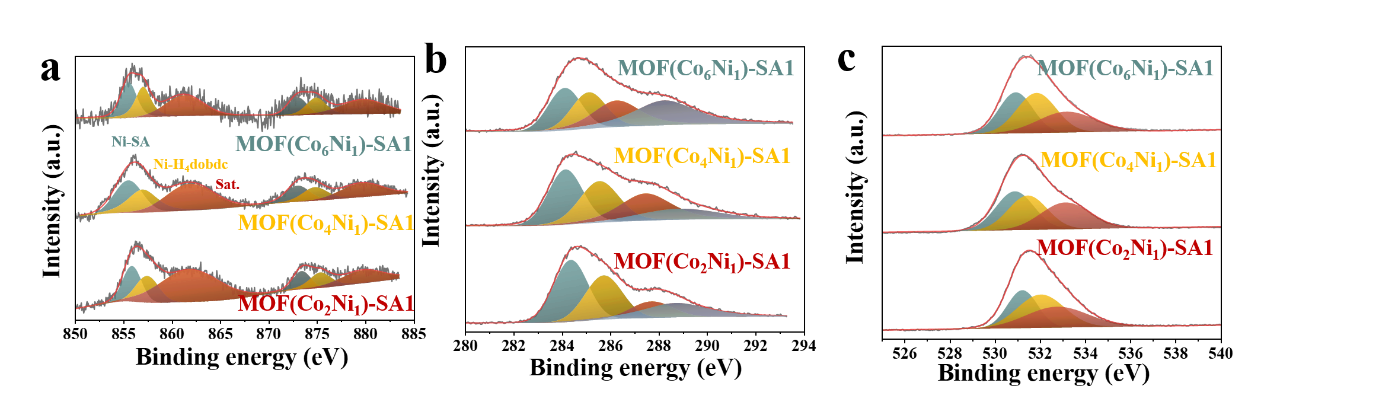


**Figure S12.** The high-resolution XPS spectra of (a) Ni 2p, (b) C 1s, and (c) O 1s.

The peaks in the C 1s and O 1s spectra (Figure S12), which exhibit similar peaks to those of MOF(Co)-SA1, progressively shift toward higher binding energy with elevated Ni content, indicating an increase in defect concentration within the material structure.

**Table S4.** The content of Co^3+^, Co-SA, and Co-dobdc in the Co 2p spectra.

| **Samples** | **Co^3+^** | **Co-SA** | **Co-dobdc** |
| --- | --- | --- | --- |
| **MOF(Co_6_Ni1)-SA1** | 25.9% | 37.3% | 36.8% |
| **MOF(Co_4_Ni1)-SA1** | 42.1% | 29.3% | 28.6% |
| **MOF(Co_2_Ni1)-SA1** | 48.9% | 25.6% | 25.5% |

**Table S5.** The content of Ni-SA and Ni-dobdc in the Ni 2p spectra.

| **Samples** | **Ni-SA** | **Ni-dobdc** |
| --- | --- | --- |
| **MOF(Co_6_Ni1)-SA1** | 51.5% | 48.5% |
| **MOF(Co_4_Ni1)-SA1** | 51.6% | 48.4% |
| **MOF(Co_2_Ni1)-SA1** | 52.2% | 47.8% |


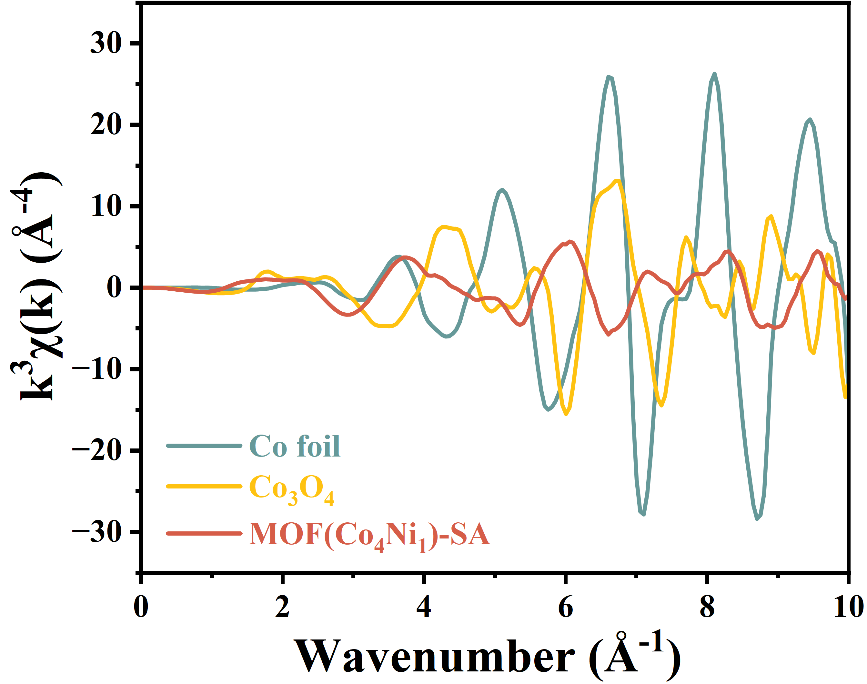


**Figure S13.** CoK-edge of *k*^3^χ(k) oscillation curves of MOF(Co_4_Ni_1_)-SA1 with a Co foil, and Co_3_O_4_ as the reference.


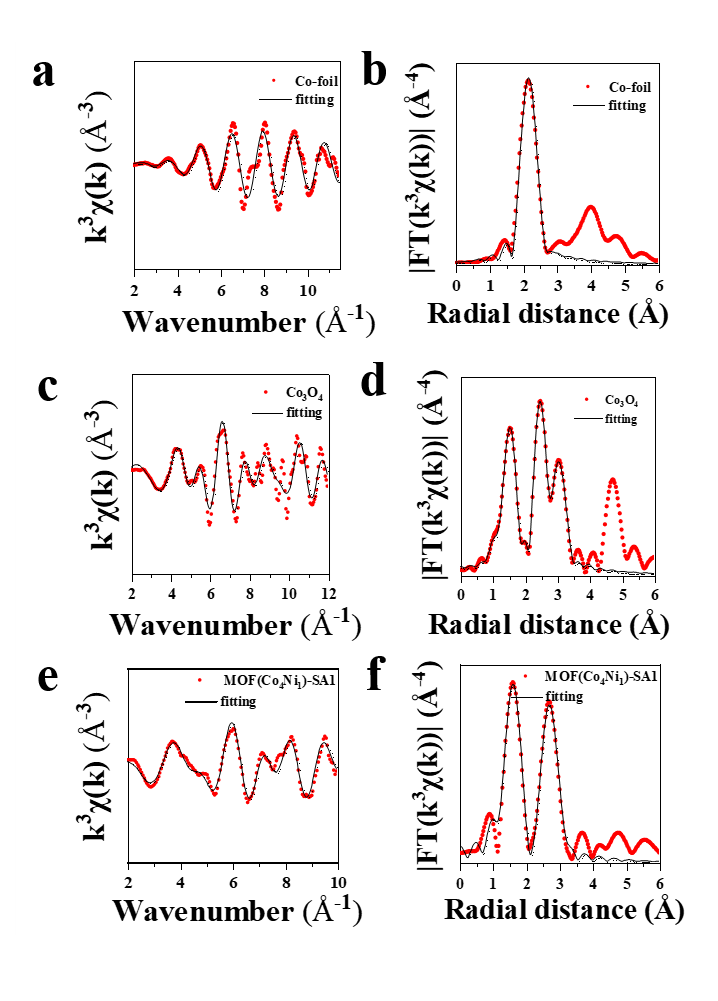


**Figure S14.** Co K-edge EXAFS spectra of Co foil (a,b), Co_3_O_4_ (c,d), and MOF(Co_4_Ni_1_)-SA1 (e, f). Fitting of the magnitude of the Fourier transform of the k^3^-weighted EXAFS. Measured and calculated spectra are matched very well. The best-fit parameters are shown in Table S6.

Table S6. EXAFS fitting parameters at the Co K-edge for various samples.

| Sample | Shell | CN^a^ | R(Å)^b^ | σ^2^(Å^2^ )^c^ | ΔE^0^(eV)^d^ | K-range/Å^-1^ | R-range/Å | R factor |
| --- | --- | --- | --- | --- | --- | --- | --- | --- |
| Co foil | Co-Co | 12* | 2.49±0.01 | 0.0054±0.0008 | 7.7±0.4 | 2.5-11.5 | 1.5-2.8 | 0.0086 |
| Co_3_O_4_ | Co-O | 4.0±0.1 | 1.92±0.01 | 0.0025±0.0004 | 3.9±0.2 | 3.0-12.0 | 1.0-3.5 | 0.0010 |
|  | Co-Co | 3.5±0.3 | 2.86±0.01 | 0.0036±0.0005 | 1.7±0.3 |  |  |  |
|  | Co-Co | 7.5±0.6 | 3.35±0.01 | 0.0069±0.0006 | 0.4±0.3 |  |  |  |
| MOF | Co-O | 4.2±0.5 | 2.06±0.01 | 0.0044±0.0015 | -1.3±0.6 | 3.0-10.0 | 1.2-3.3 | 0.0049 |
|  | Co-M | 6.5±1.2 | 3.12±0.01 | 0.0104±0.0019 | -0.9±0.6 |  |  |  |

^a^CN, coordination number; ^b^R, the distance between absorber and backscatter atoms; ^c^σ ^2^, Debye-Waller factor, Debye-Waller factor to account for both thermal and structural disorders; ^d^ΔE_0_, inner potential correction; R factor indicates the goodness of the fit. S0 ^2^ was fixed to 0.738, according to the experimental EXAFS fit of Co foil by fixing CN as the known crystallographic value. * This value was fixed during EXAFS fitting. Error bounds that characterize the structural parameters obtained by EXAFS spectroscopy were estimated as CN ± 20%; R ± 1%; σ2 ± 20%; ΔE0 ± 20%. A reasonable range of EXAFS fitting parameters: 0.700 < Ѕ0 ^2^ < 1.000; CN > 0; σ ^2^ > 0 Å^2^; |ΔE_0_| < 15 eV; R factor < 0.02. *MOF is the abbreviation of MOF(Co_4_Ni_1_)-SA1. **Co-Co and Co-M are abbreviation of Co-O-Co and Co-O-M in the Co_3_O_4_ and MOF(Co_4_Ni_1_)-SA1, respectively.


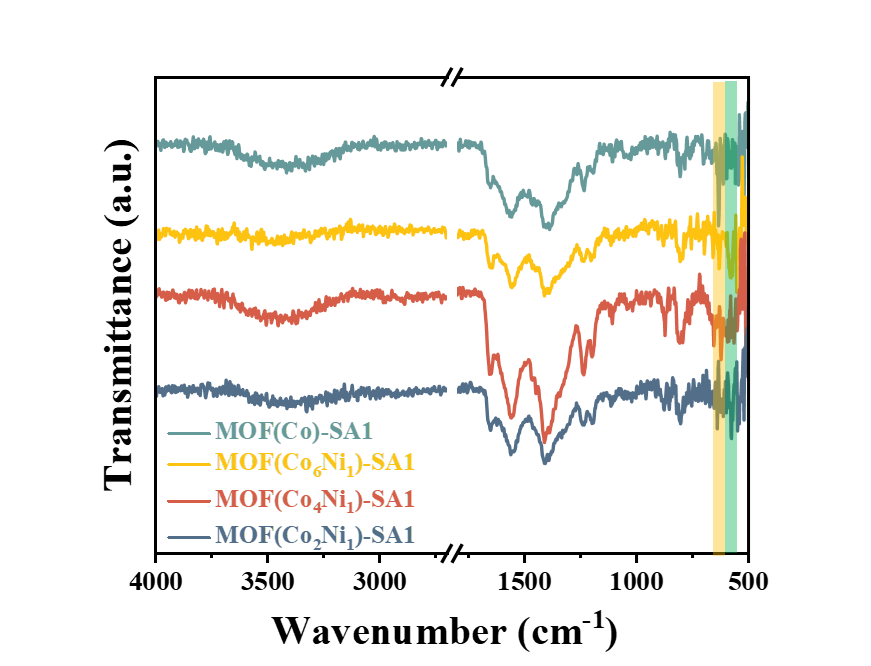


**Figure S15.** The FTIR spectra of MOF (Co*_x_*Ni*_y_*)-SA1.

In the FTIR spectra (Figure S15), with the content of Ni increasing, MOF (Co*_x_*Ni*_y_*)-SA1 maintains the structure of the defective MOF, and vibration peaks of Co–O and Ni–O appear at 622.8 and 576.6 cm^−1^, respectively, which proves the Ni doping successfully in the material structure^[16]^.


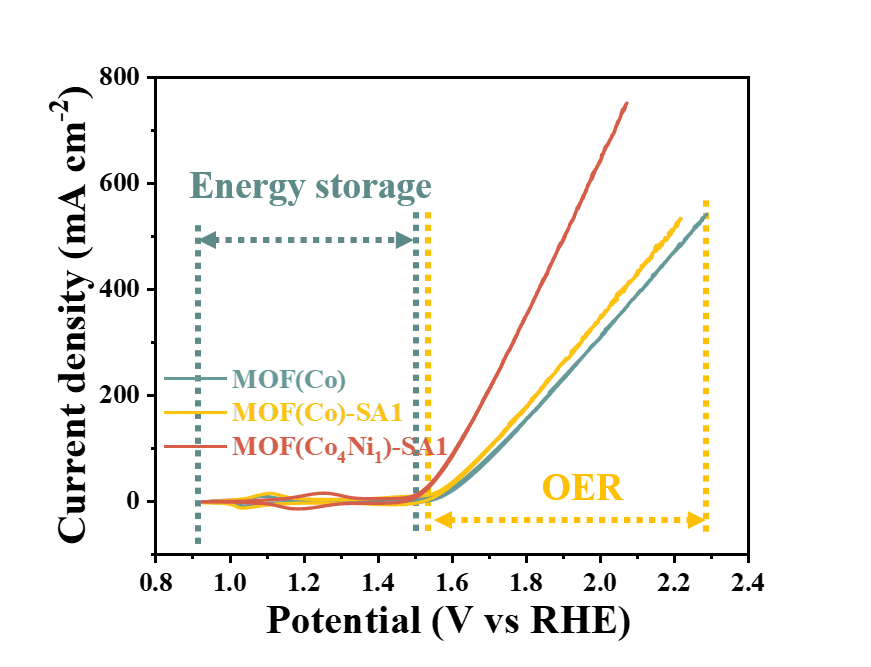


**Figure S16.** The CV curves of MOF(Co), MOF(Co)-SA1, and MOF(Co_4_Ni_1_)-SA1 from the energy storage region and OER region at 5 mV s^−1^ in 1 M KOH aqueous solution without *i*R correction.


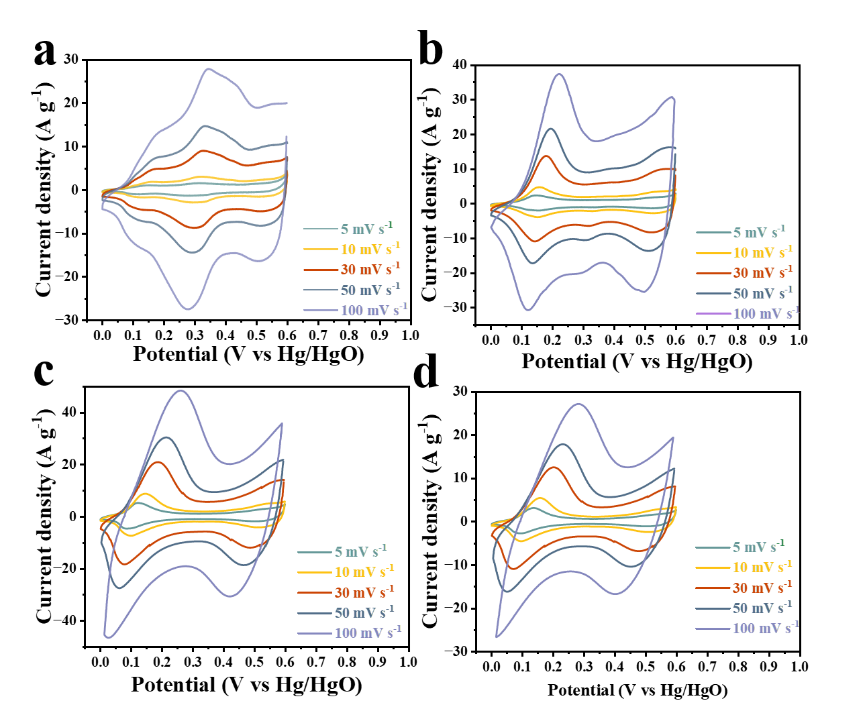


**Figure S17.** The CV curves of MOF(Co)-*t* (*t* =300, 600, 900, 1200) electrodes at various scan rates (5-100 mV s^−1^).


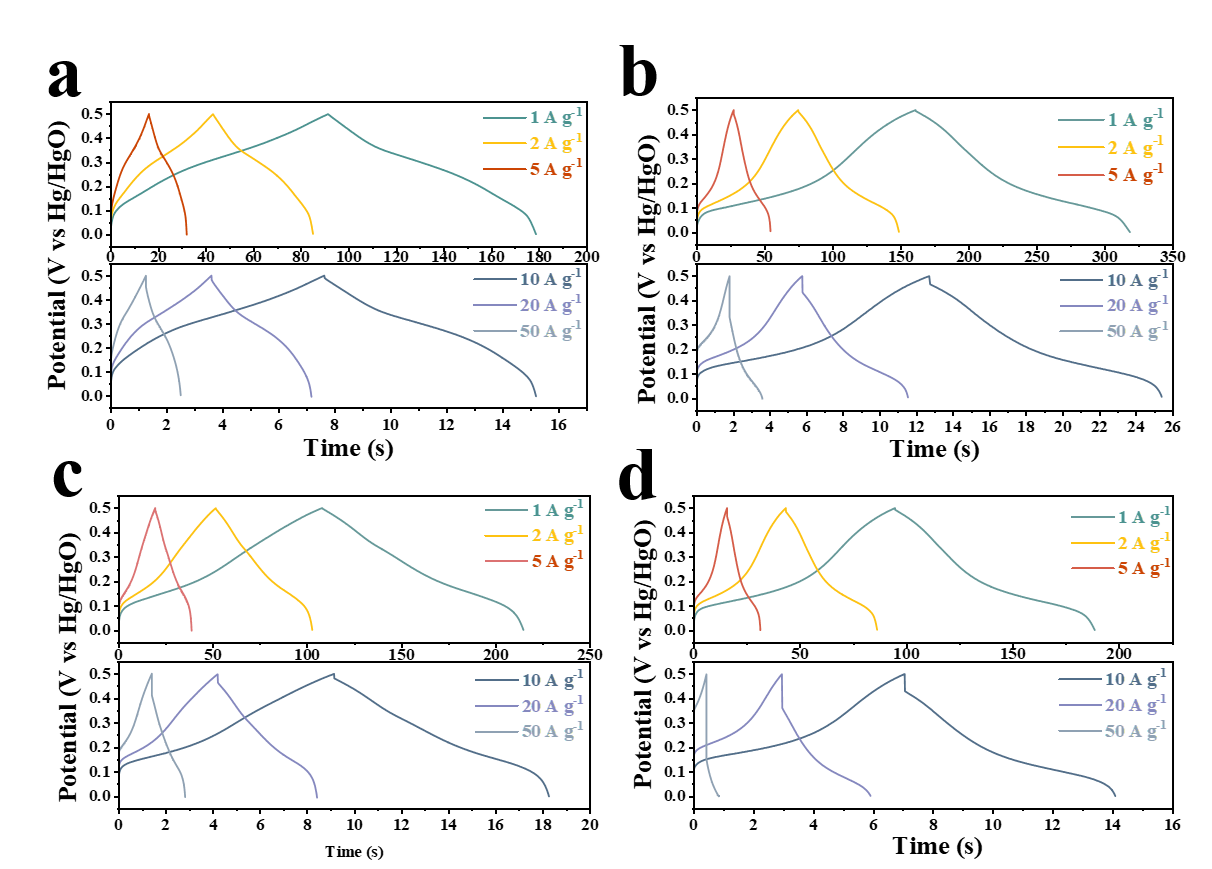


**Figure S18.** The GCD curves of MOF(Co)-*t* electrodes at various current densities (1-50 A g^−1^).


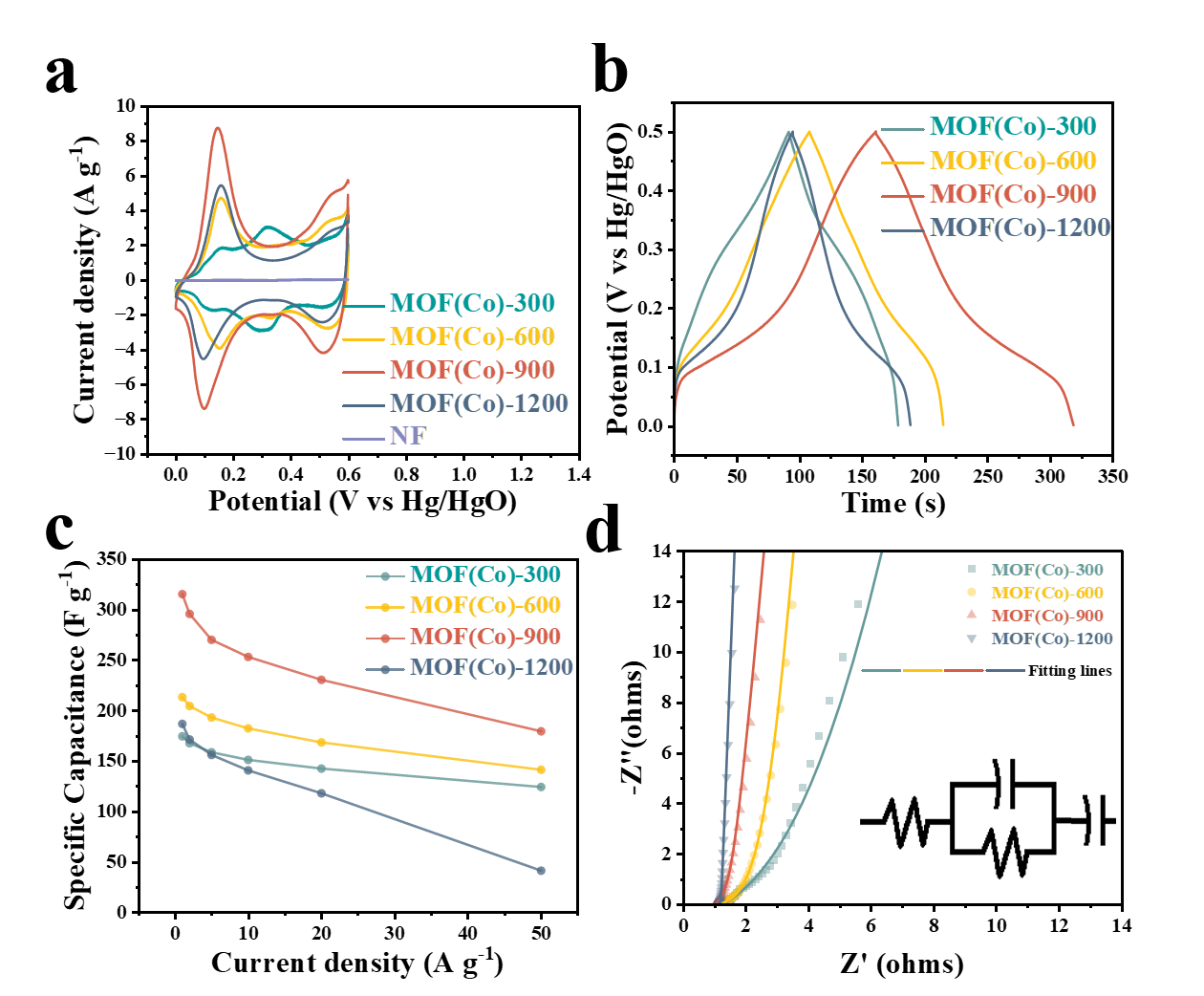


**Figure S19.** (a)The CV curve of MOF(Co)-*t* electrodes at 5 mV s^−1^. (b) The GCD curve of MOF(Co)-*t* electrodes at 1 A g^−1^. (c) The specific capacitance of MOF(Co)-*t* electrodes*.* (d) Nyquist plot and fitting lines. The inset shows the equivalent circuit model.

**Table S7.** The fitting values of R_s_, R_ct_, and C_dl_.

| **Samples** | **R_s_ (E.%)** | **R_ct_ (E.%)** | **C_dl_ (E.%)** |
| --- | --- | --- | --- |
| **MOF(Co)-300** | 1.59 (3.71 %) | 2.46 (10.38 %) | 0.01 (14.44%) |
| **MOF(Co)-600** | 1.39 (1.82 %) | 0.94 (8.25 %) | 0.06 (13.67%) |
| **MOF(Co)-900** | 1.09 (0.99 %) | 0.51 (8.03 %) | 0.67 (11.06%) |
| **MOF(Co)-1200** | 1.16 (0.34 %) | 0.12 (10.72 %) | 2.88 (15.29%) |


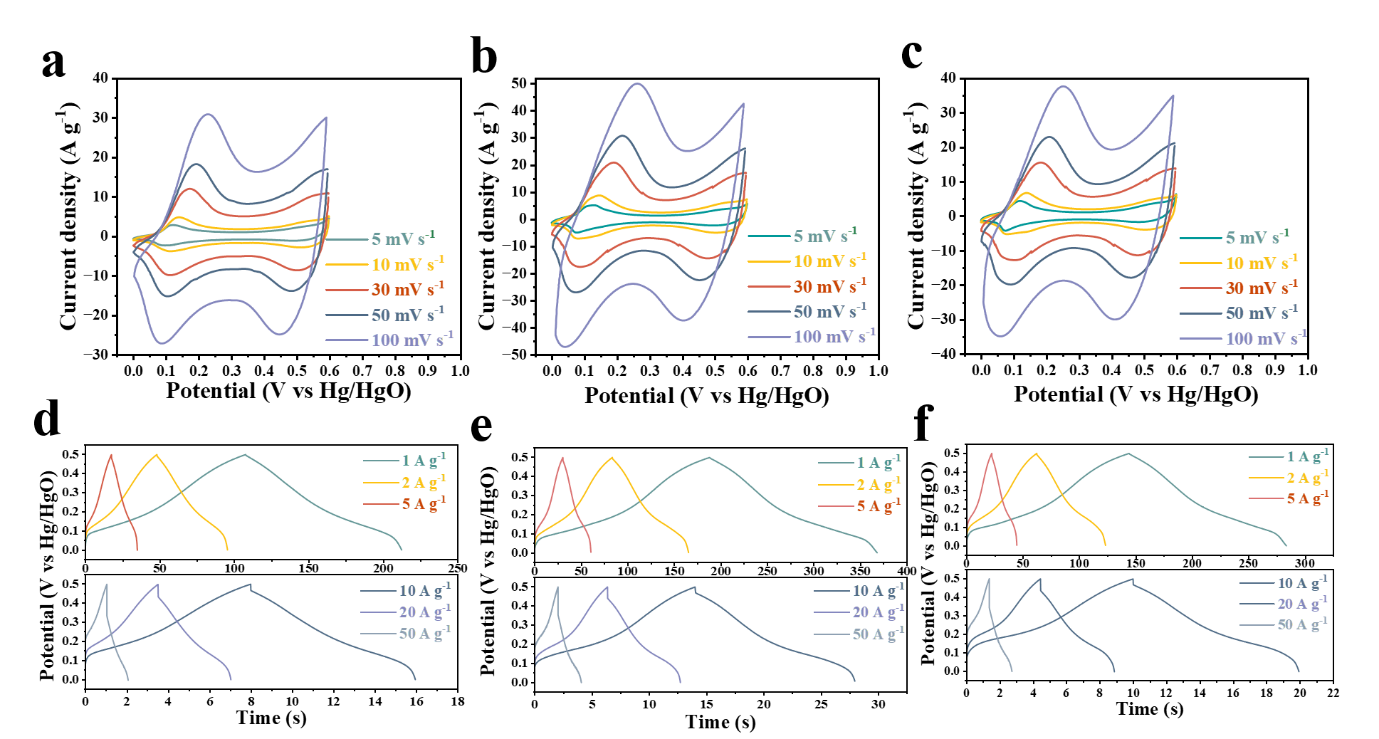


**Figure S****20.** The CV and GCD curves of MOF(Co)-SA*x.* (a, d) MOF(Co)-SA0.5. (b, e) MOF(Co)-SA1. (c, f) MOF(Co)-SA2*.*


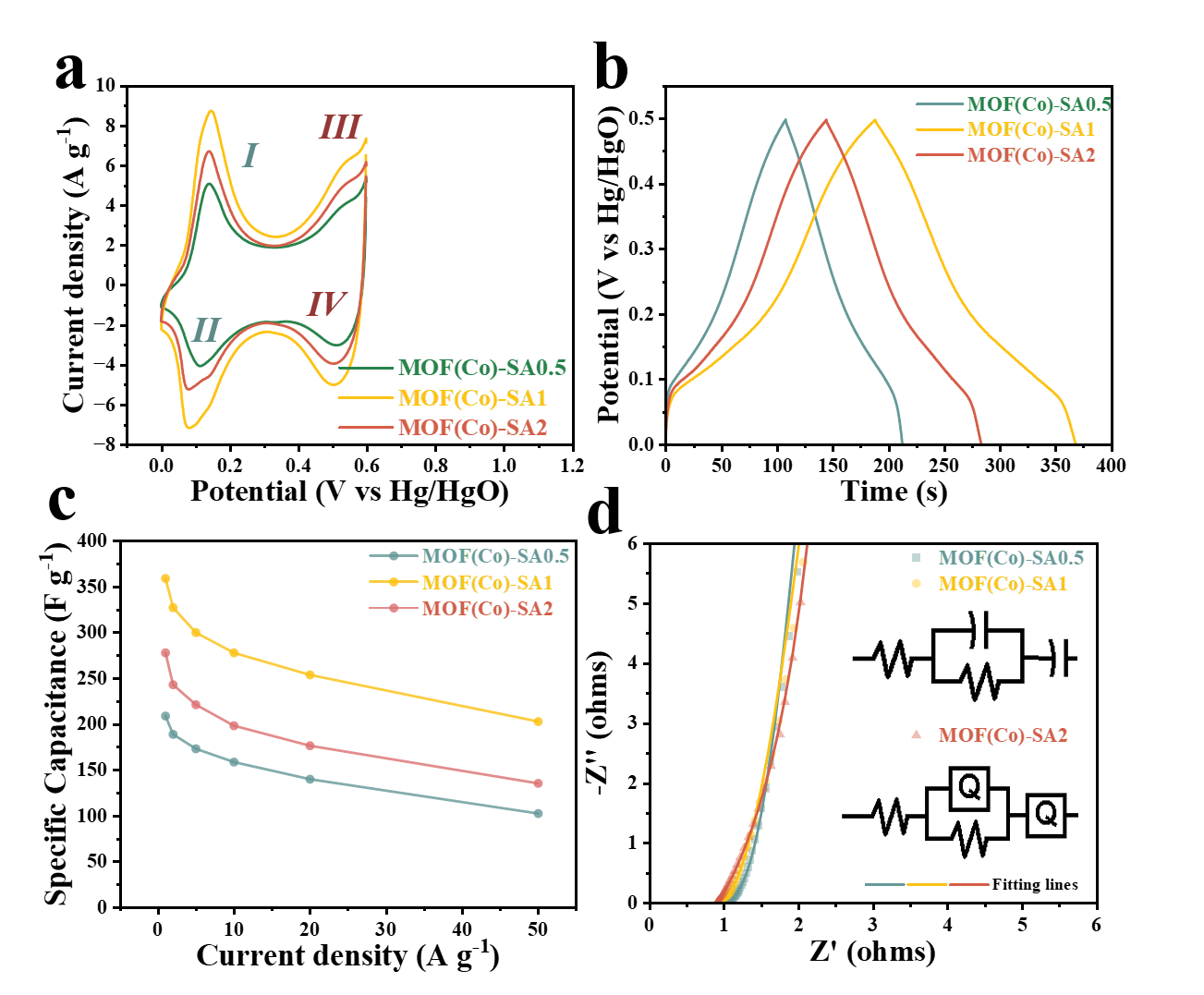


**Figure S21.** (a)The CV curve of MOF(Co)-SA*x* electrodes at 5 mV s^−1^. (b) The GCD curve of MOF(Co)-SA*x* electrodes at 1 A g^−1^. (c) The specific capacitance of MOF(Co)-SA*x* electrodes*.* (d) Nyquist plot and fitting lines. The inset shows the equivalent circuit model.

**Table S8.** The fitting values of R_s_, R_ct_, and C_dl_.

| **Samples** | **R_s_ (E.%)** | **R_ct_ (E.%)** | **C_dl_ (E.%)** |
| --- | --- | --- | --- |
| **MOF(Co)-SA0.5** | 1.07 (1.08 %) | 0.48 (9.19 %) | 0.33 (13.2%) |
| **MOF(Co)-SA1** | 1.01 (0.93 %) | 0.64 (8.53 %) | 0.69 (9.53%) |
| **MOF(Co)-SA2** | 0.94 (0.13 %) | 1.81 (8.49 %) | 0.98 (1.22%) |


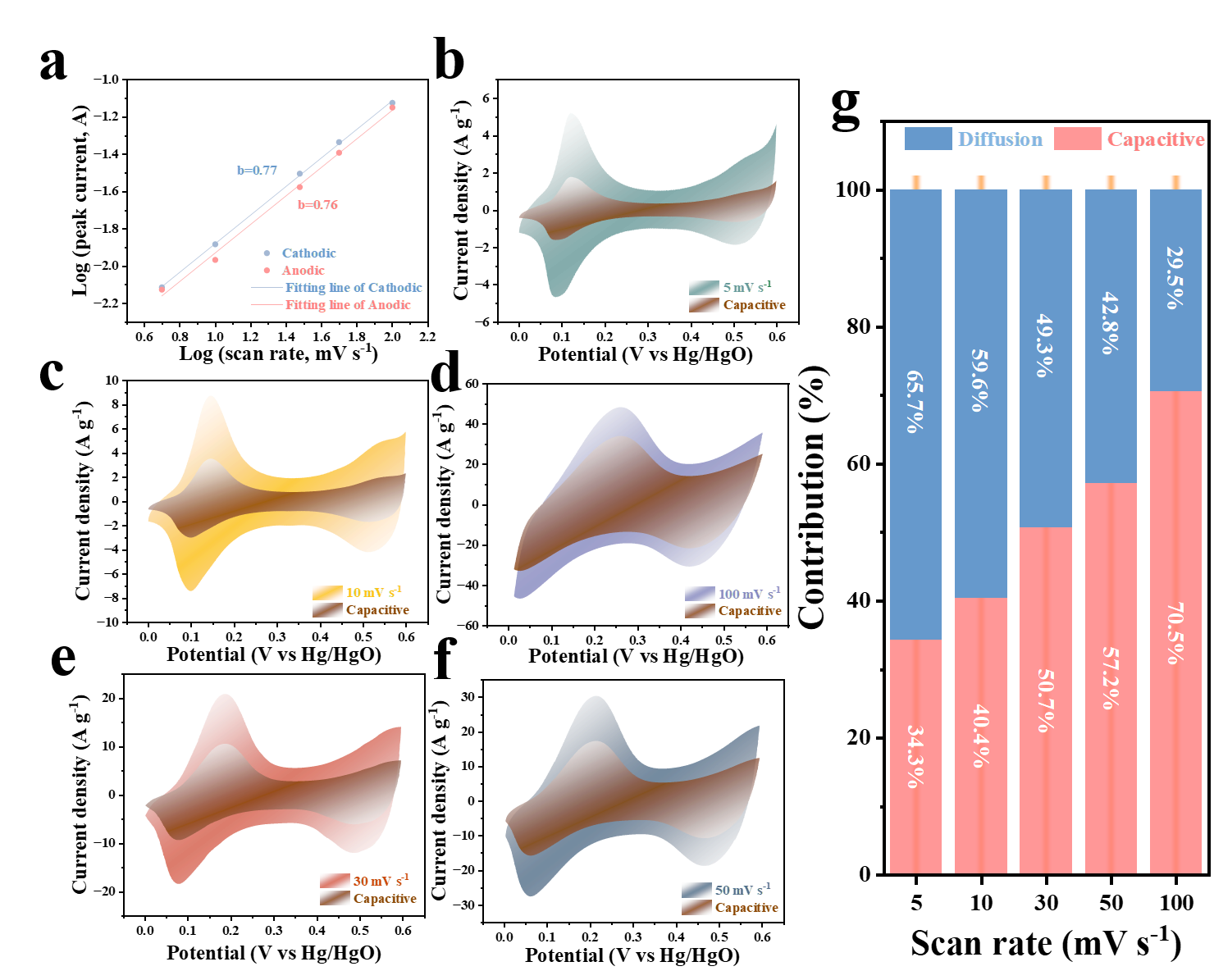


**Figure S22.** Electrochemical kinetics of MOF (Co) analysis. (a) log(i)-log(v) plots. (b-f) capacitive/diffusive contributions at various scan rates. (g) capacitive contribution ratio of diffusion/capacitive-controlled processes.


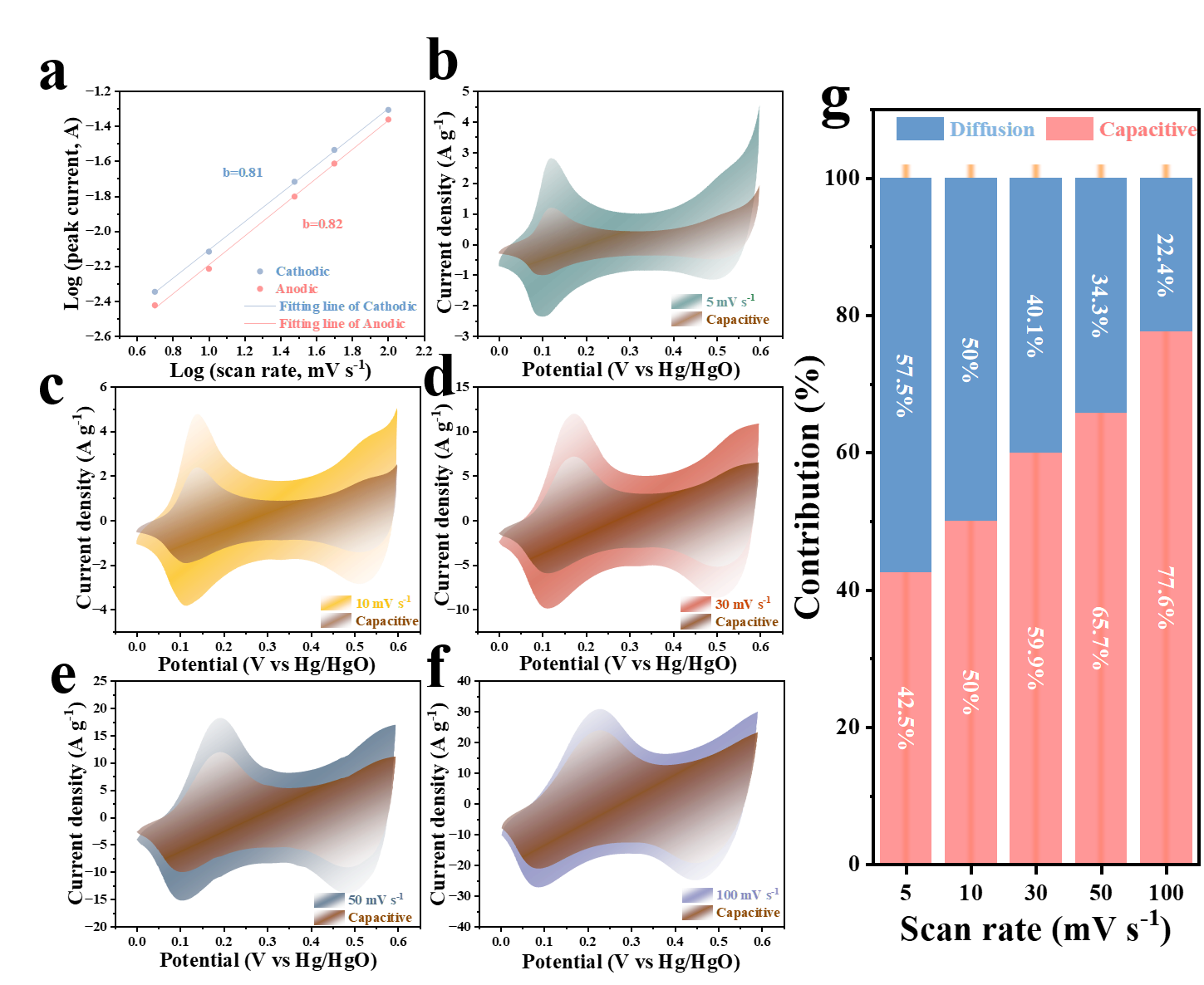


**Figure S23.** Electrochemical kinetics of MOF (Co)-SA0.5 analysis. (a) log(i)-log(v) plots. (b-f) capacitive/diffusive contributions at various scan rates. (g) capacitive contribution ratio of diffusion/capacitive-controlled processes.


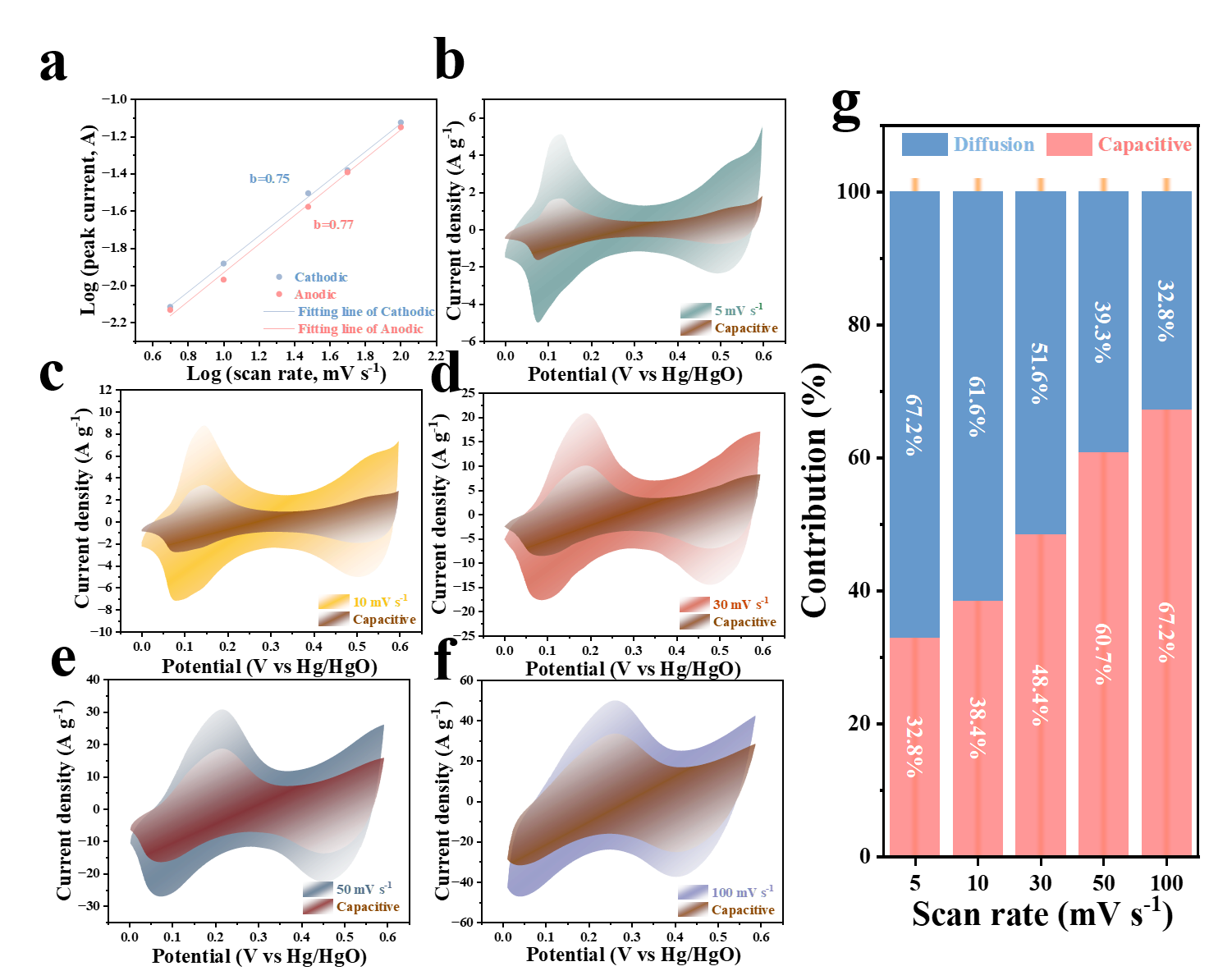


**Figure S24.** Electrochemical kinetics of MOF (Co)-SA1 analysis. (a) log(i)-log(v) plots. (b-f) capacitive/diffusive contributions at various scan rates. (g) capacitive contribution ratio of diffusion/capacitive-controlled processes.


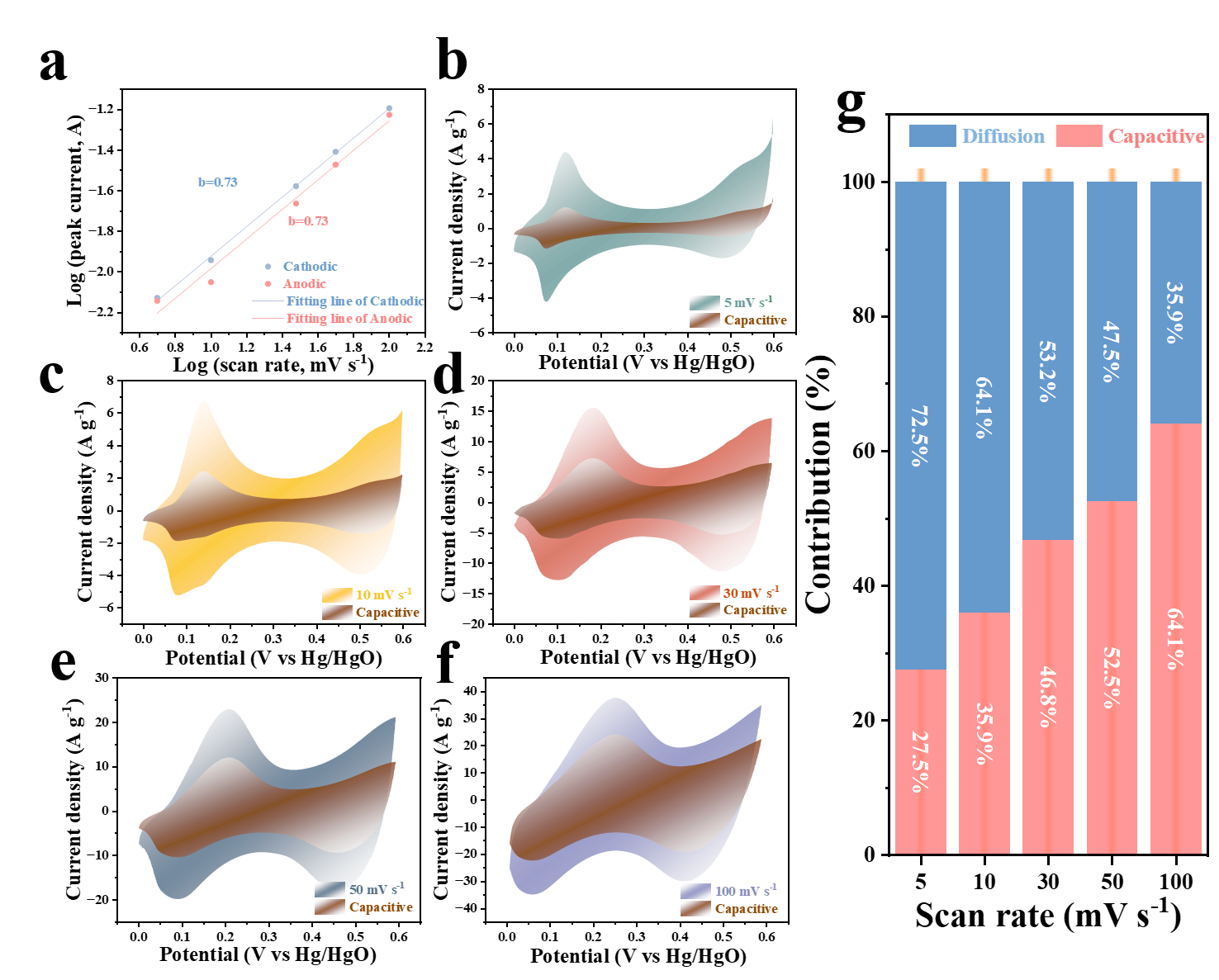


**Figure S25.** Electrochemical kinetics of MOF (Co)-SA2 analysis. (a) log(i)-log(v) plots. (b-f) capacitive/diffusive contributions at various scan rates. (g) capacitive contribution ratio of diffusion/capacitive-controlled processes.


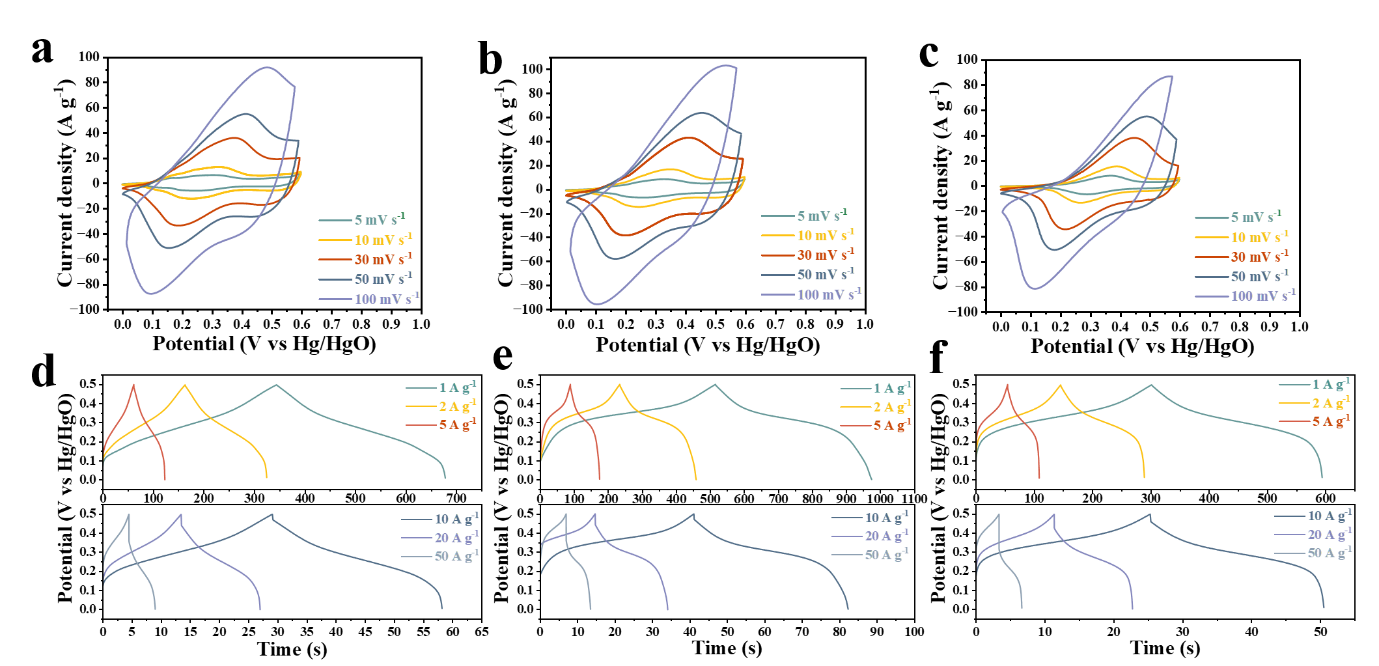


**Fig****ure S26.** The CV and GCD curves of MOF (Co*_y_*Ni*_z_*)-SA1*.* (a, d) MOF (Co_6_Ni_1_)-SA1. (b, e) MOF (Co_4_Ni_1_)-SA1. (c, f) MOF (Co_2_Ni_1_)-SA1

**Table S9.** The fitting values of R_s_, R_ct_, and C_dl_.

| **Samples** | **Rs (E.%)** | **Rct (E.%)** | **Cdl (E.%)** |
| --- | --- | --- | --- |
| **MOF(Co)** | 1.09 (0.99 %) | 0.51 (8.03 %) | 0.67 (11.06%) |
| **MOF(Co)-SA1** | 1.01 (0.93 %) | 0.64 (8.53 %) | 0.69 (9.53%) |
| **MOF(Co6Ni1)-SA1** | 0.88 (0.27 %) | 0.97 (16.95 %) | 1.25 (4.17%) |
| **MOF(Co4Ni1)-SA1** | 0.81 (0.439 %) | 0.46 (21.39 %) | 1.59 (10.03%) |
| **MOF(Co2Ni1)-SA1** | 1.13 (1.31 %) | 1.20 (9.20 %) | 0.63 (1.33%) |


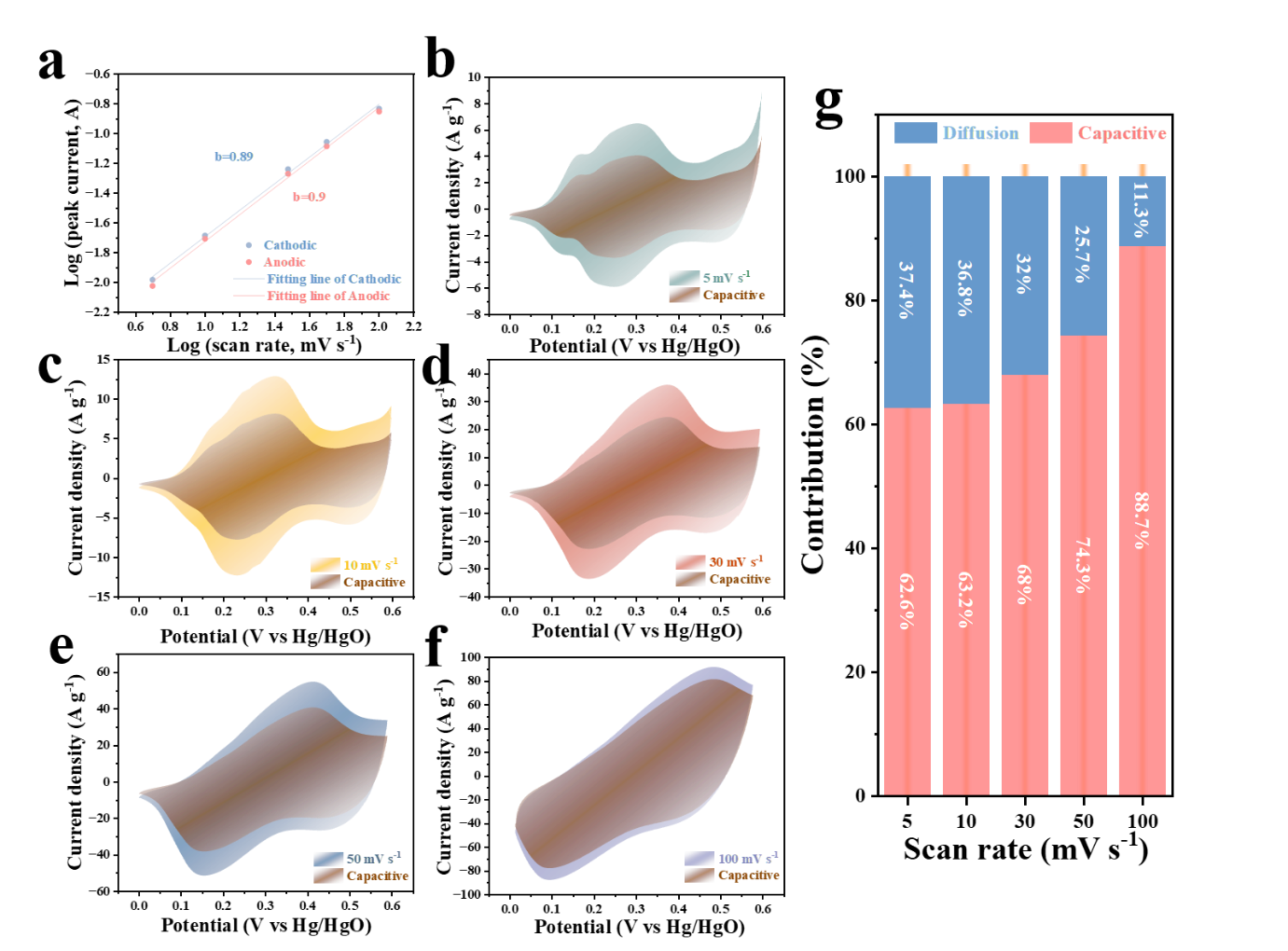


**Figure S27.** Electrochemical kinetics of MOF (Co_6_Ni_1_)-SA1 analysis. (a) log(i)-log(v) plots. (b-f) capacitive/diffusive contributions at various scan rates. (g) capacitive contribution ratio of diffusion/capacitive-controlled processes.


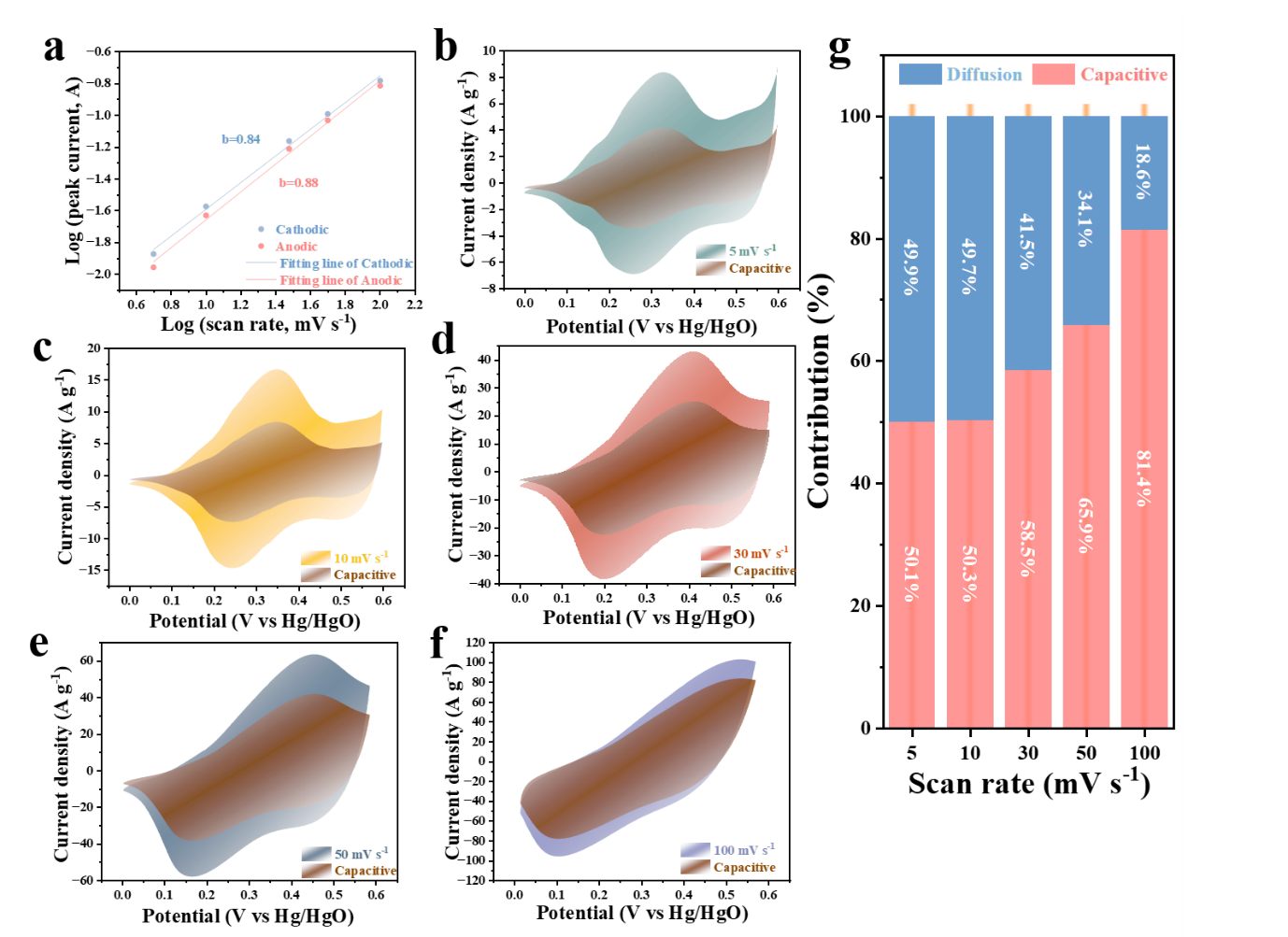


**Figure S28.** Electrochemical kinetics of MOF (Co_4_Ni_1_)-SA1 analysis. (a) log(i)-log(v) plots. (b-f) capacitive/diffusive contributions at various scan rates. (g) capacitive contribution ratio of diffusion/capacitive-controlled processes.


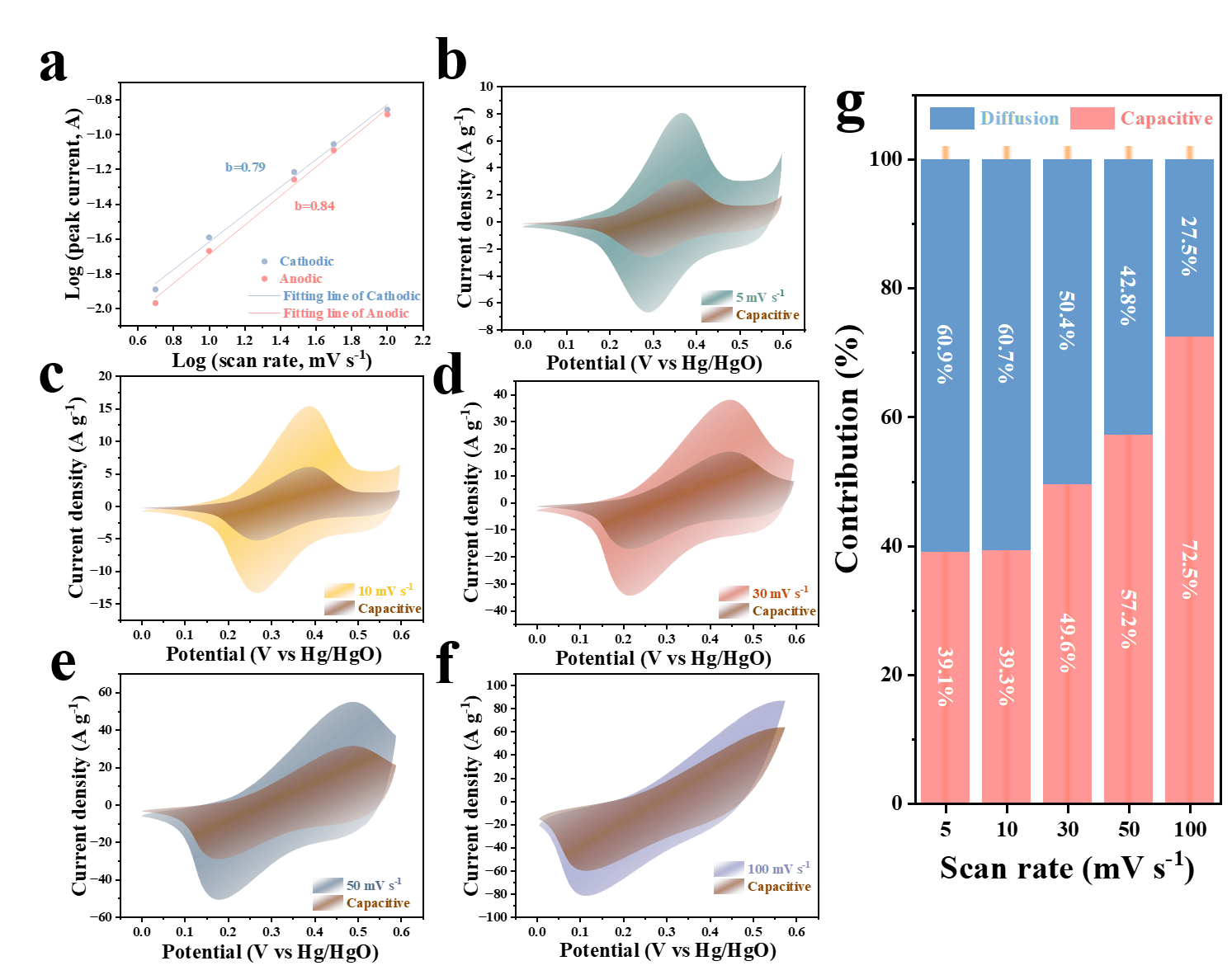


**Figure S29.** Electrochemical kinetics of MOF (Co_2_Ni_1_)-SA1 analysis. (a) log(i)-log(v) plots. (b-f) capacitive/diffusive contributions at various scan rates. (g) capacitive contribution ratio of diffusion/capacitive-controlled processes.


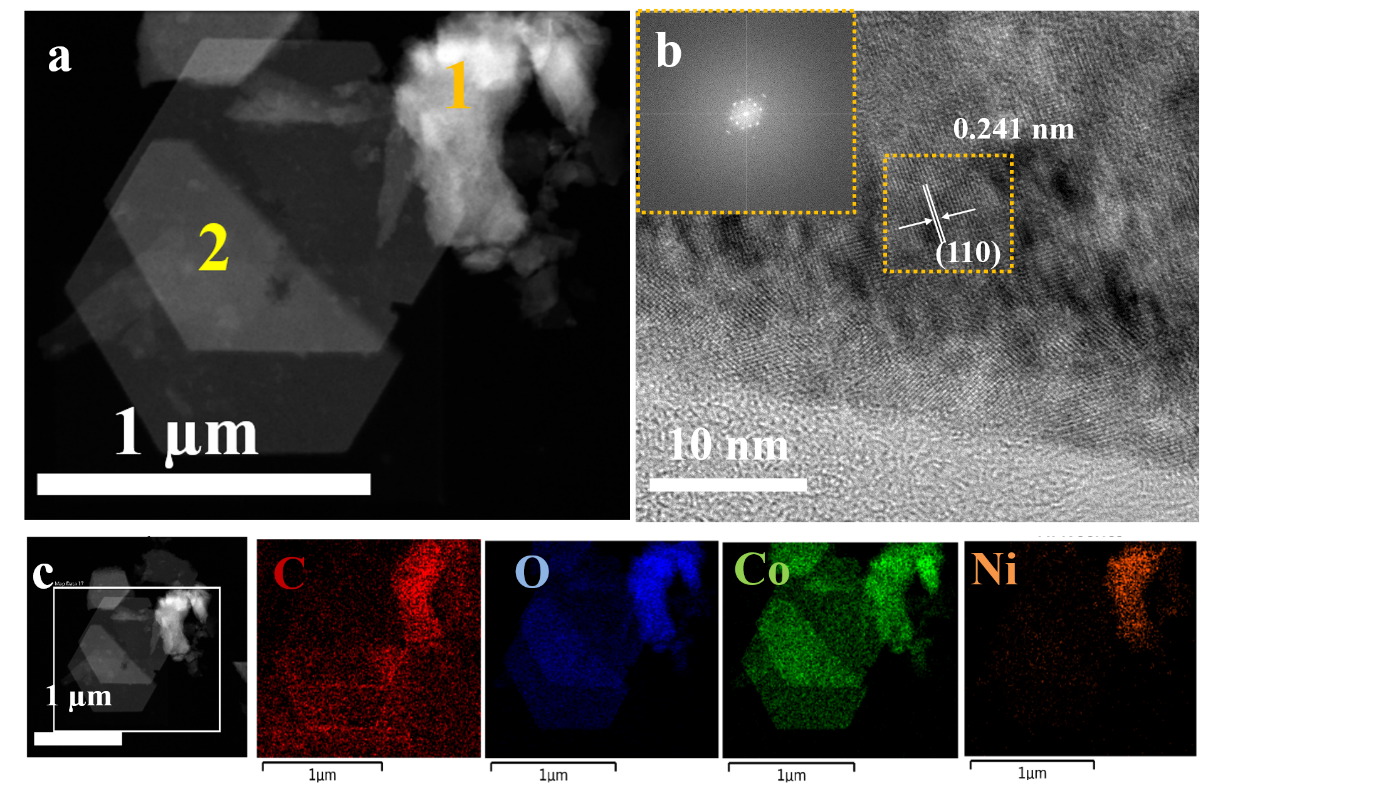


**Figure S30.** (a) The HAADF-STEM of MOF (Co_4_Ni_1_)-SA1, the positions marked as 1 and 2. (b) HRTEM images of MOF (Co_4_Ni_1_)-SA1(Position 2), the inset shows FFT of CoOOH. (c) TEM-mapping images.

**Table. S10.** The element content in positions 1 and 2.

| **Position** | **Co** | **Ni** | **O** | **C** |
| --- | --- | --- | --- | --- |
| **1. MOF(Co_4_Ni_1_)-SA1** | 10.25 | 2.99 | 55.10 | 31.66 |
| **2. CoOOH** | 22.87 | 0.28 | 76.85 |  |


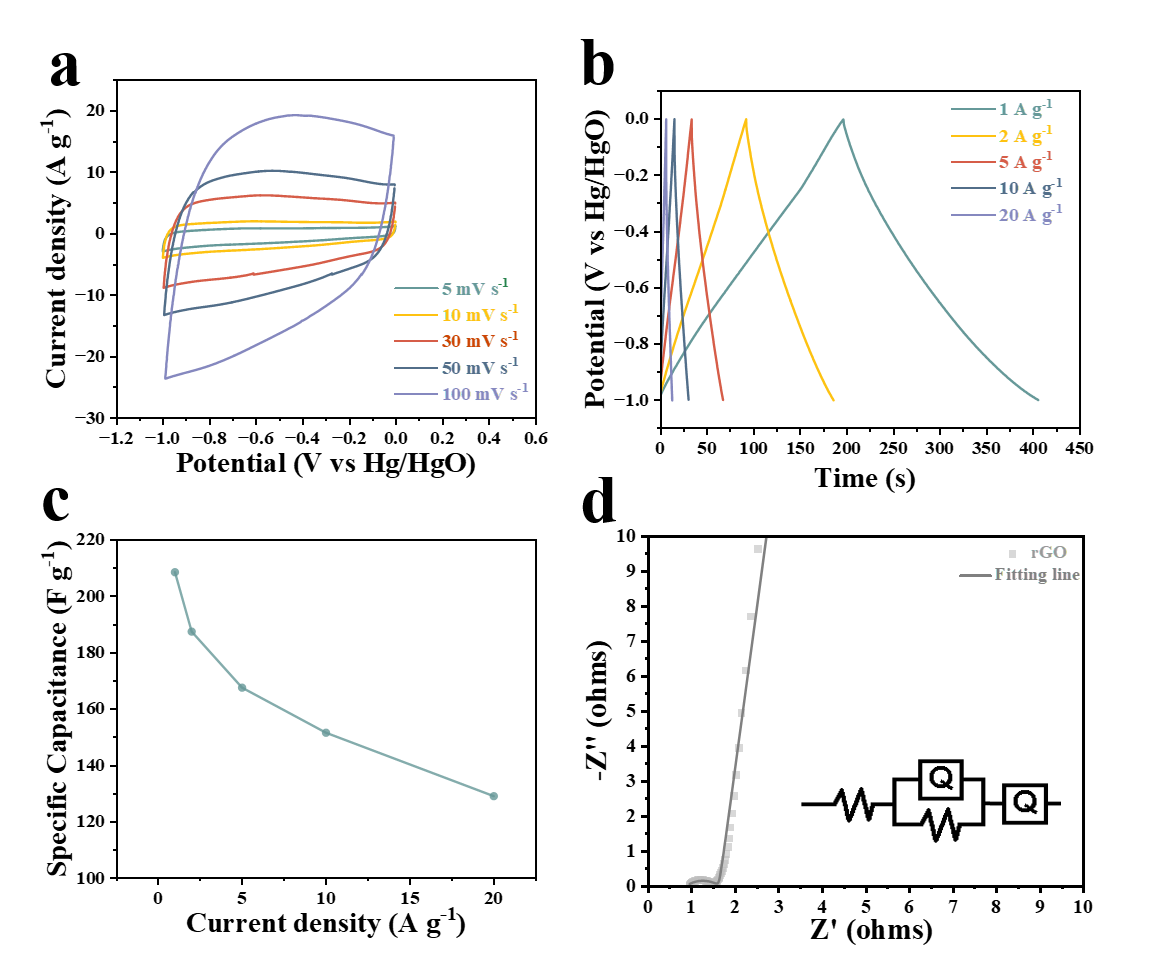


**Figure S31.** (a) The CV curves of commercial rGO at various scan rates. (b) The GCD curves at different current densities. (c) The specific capacitance. (d) Nyquist plot and fitting lines. The inset shows the equivalent circuit model.

**Table S11.** The fitting values of R_s_, R_ct_, and C_dl_.

| **Samples** | R_s_ (E.%) | R_ct_ (E.%) | C_dl_ (E.%) |
| --- | --- | --- | --- |
| **rGO** | 0.88 (3.23 %) | 0.77 (5.07 %) | 0.51 (6.86 %) |


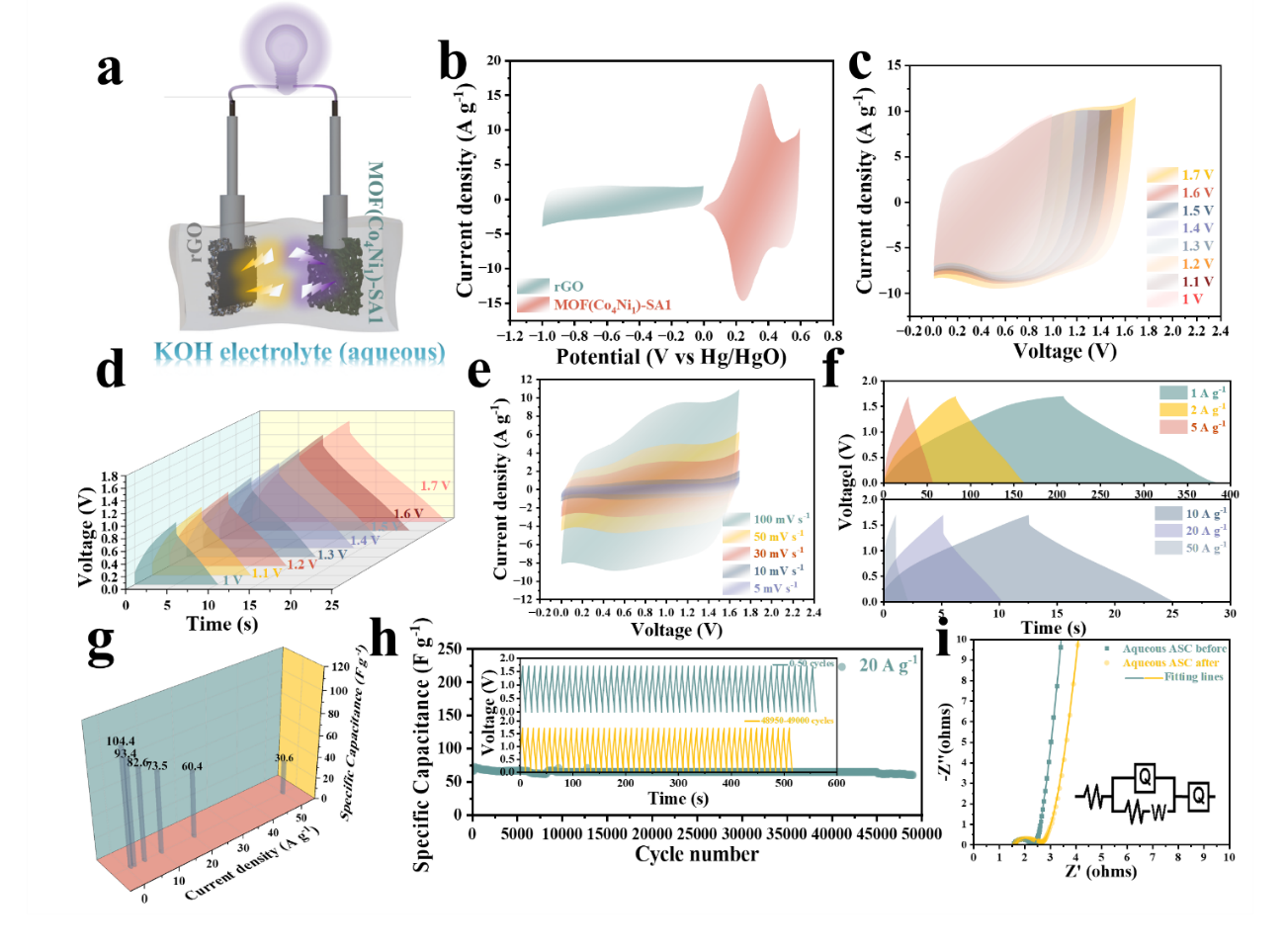


**Figure S32.** Electrochemical performance of MOF (Co_4_Ni_1_)-SA1 ASC device. (a) ASC structure; (b) CV curves of the cathode (MOF (Co_4_Ni_1_)-SA1), and anode (rGO) at 10 mV s^−1^. (c) Voltage window optimization of CV at 100 mV s^−1^. (d) The GCD curves under different voltages at 10 A g^−1^. (e) The CV curves at various scan rates (0-1.7V). (f) GCD curves at different current densities. (g) Specific capacitance. (h) Cycling stability at 20A g^−1^ with the inset showing GCD curves of 49000 cycles. (i) EIS before/after 10000 cycles with equivalent circuit (inset).


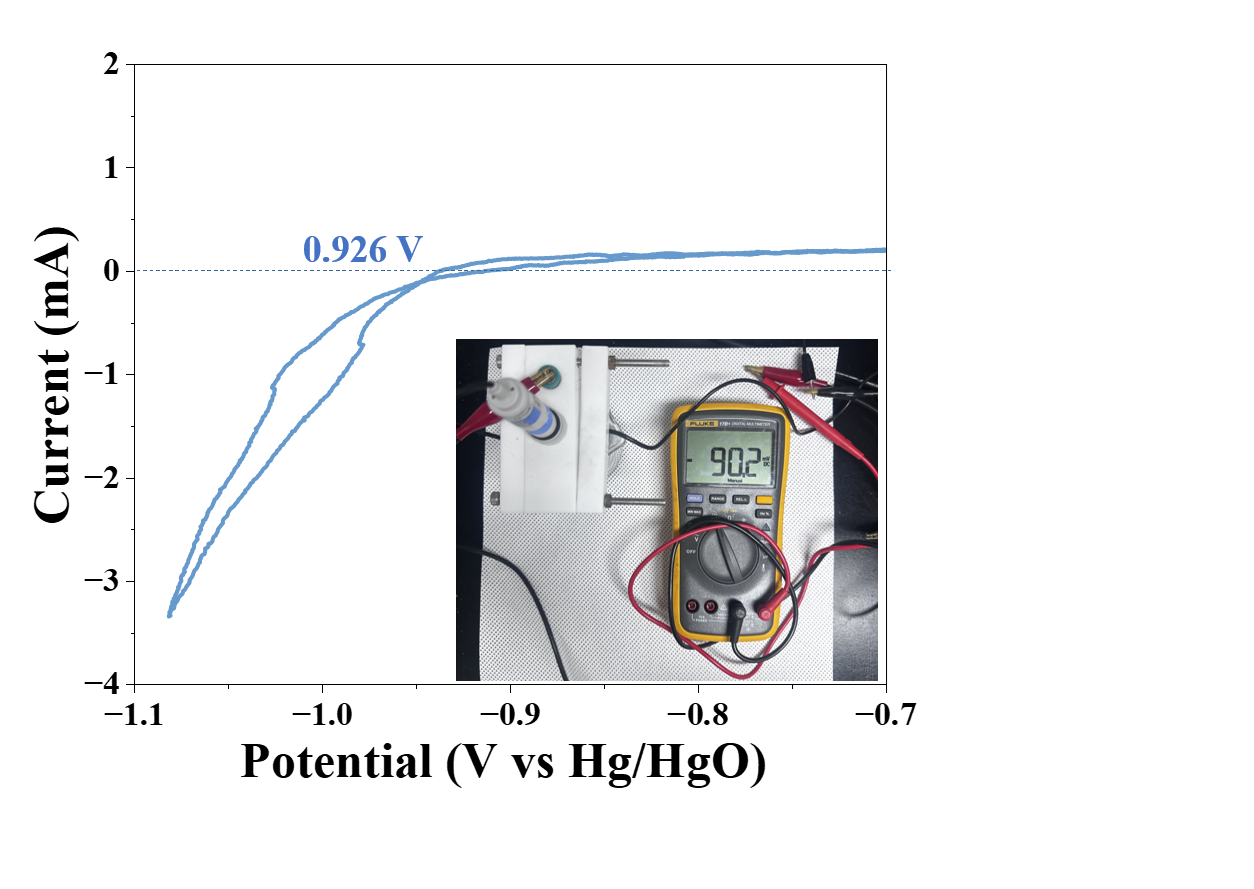


**Figure S33.** Calibration of Hg/HgO, KOH (1.0M) reference electrode in 1.0 M KOH electrolyte, the inset shows the photo of the calibration of Hg/HgO by Ag/AgCl.


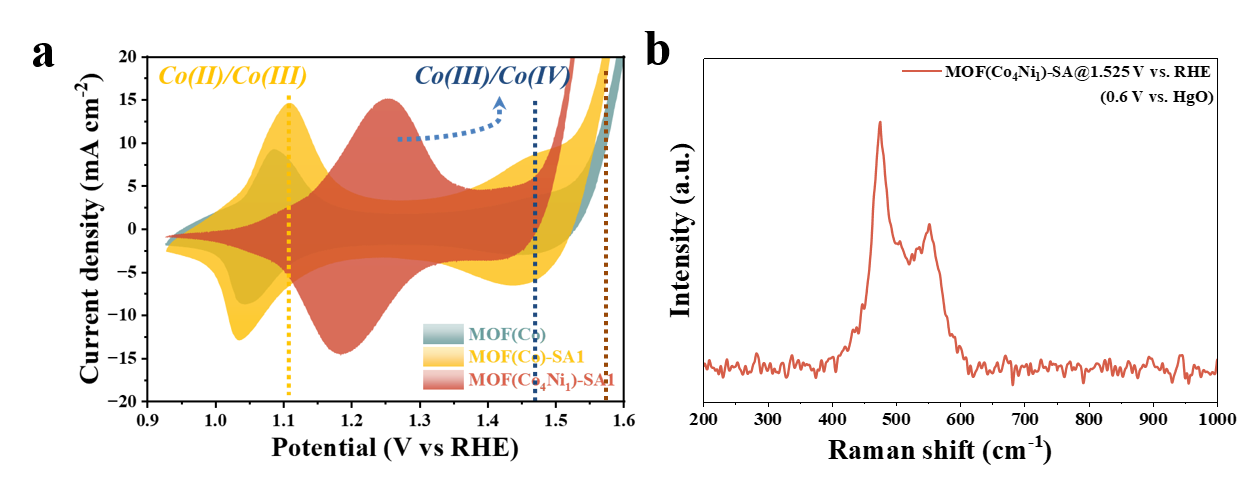


**Figure S34.** (a)The enlarged redox region CV curves of MOF(Co), MOF(Co)-SA1, and MOF(Co_4_Ni_1_)-SA1 at 5mV s^−1^ without *i*R correction. (b) The Raman spectra of and MOF(Co_4_Ni_1_)-SA1 at 1.525 V vs. RHE.


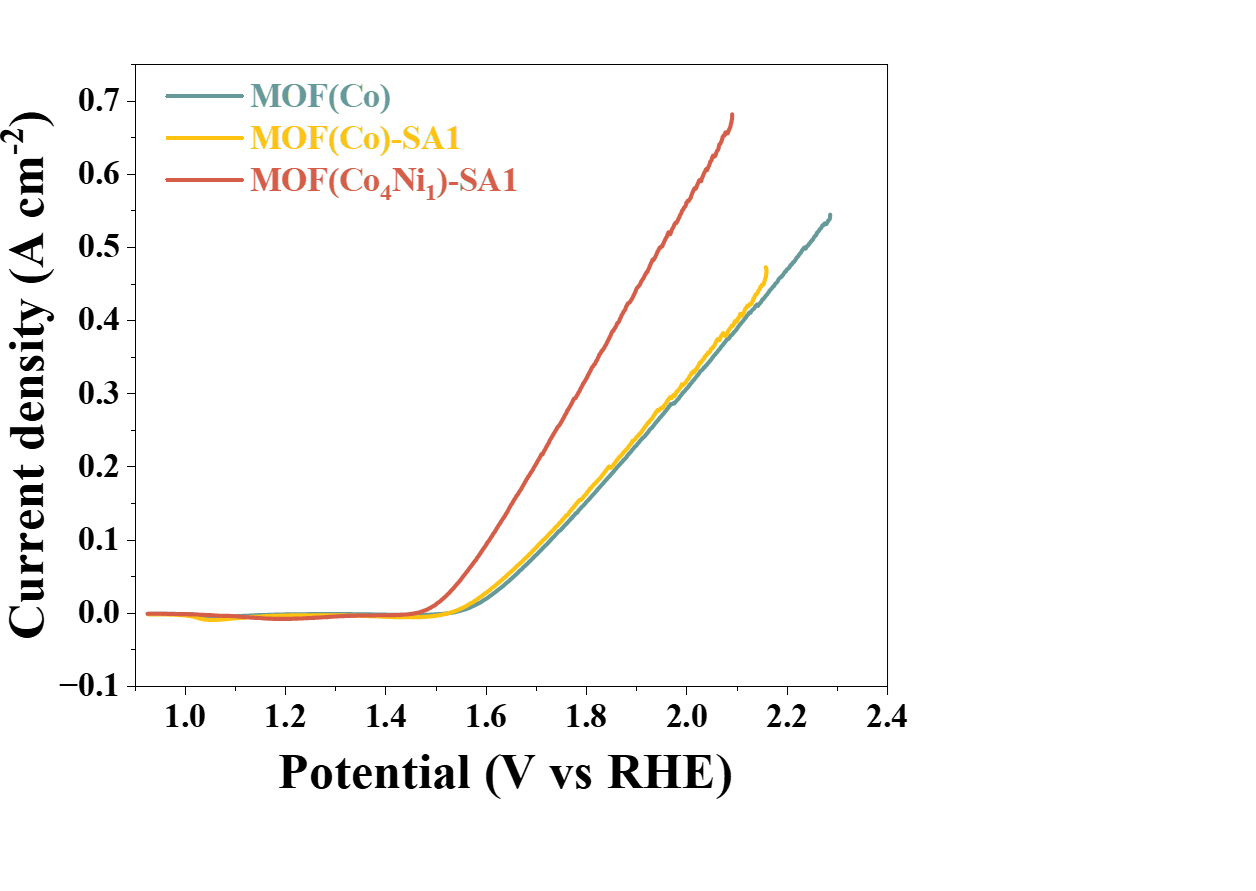


**Figure S35.** OER polarization curves of MOF(Co), MOF(Co)-SA1, and MOF (Co_4_Ni_1_)-SA1 without *iR*-correction.

**Table S12.** The OER performance of MOF(Co_4_Ni_1_)-SA1 without iR correction (unit: mV).

| Samples | Ƞ_10_ | Ƞ_100_ |
| --- | --- | --- |
| MOF(Co) | 347 | 500 |
| MOF(Co)-SA1 | 333 | 485 |
| MOF(Co_4_Ni_1_)-SA1 | **268** | **378** |

**Table S13.** The fitting values of R_s_ and R_ct_.

| **Samples** | **R_s_ (E.%)** | **R_ct_ (E.%)** |
| --- | --- | --- |
| **MOF(Co)** | 1.19 (0.17%) | 0.75 (1.29%) |
| **MOF(Co)-SA1** | 1.19 (0.23%) | 0.74 (2.56%) |
| **MOF(Co_4_Ni_1_)-SA1** | 1.11 (0.26%) | 0.68 (2.93%) |
| **Commercial RuO_2_** | 2.142 (0.527 %) | 1.965 (1.170%) |


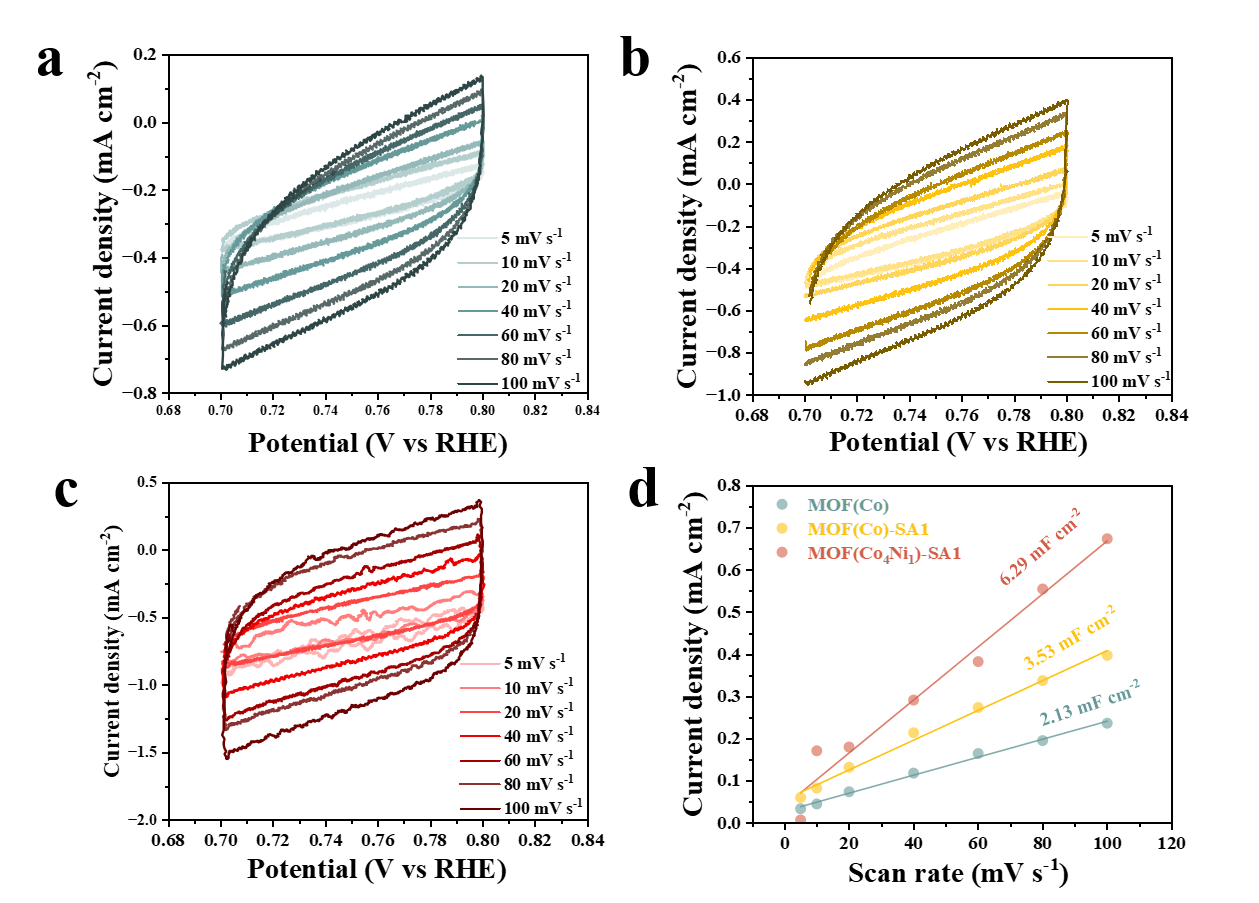


**Figure S36.** (a-c) The CV curves of MOF(Co), MOF(Co)-SA1, and MOF (Co_4_Ni_1_)-SA1 electrodes at different scan rates. (d) The fitted C_dl_ value of MOF(Co), MOF(Co)-SA1, and MOF (Co_4_Ni_1_)-SA1 electrodes.


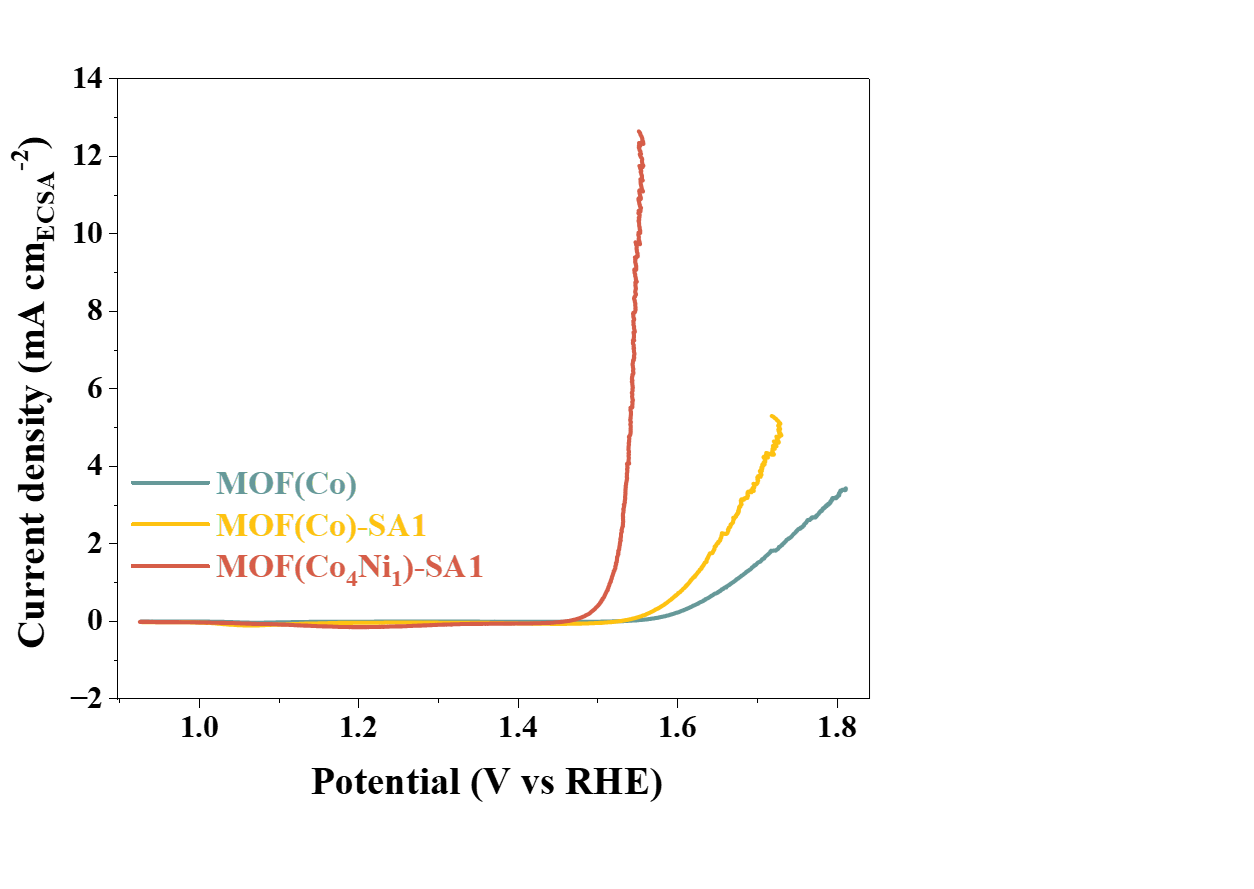


**Figure S37.** ECSA-normalized OER polarization curves **of** MOF(Co), MOF(Co)-SA1, and MOF(Co_4_Ni_1_)-SA1 at 5mV s^−1^ with 80% iR correction.


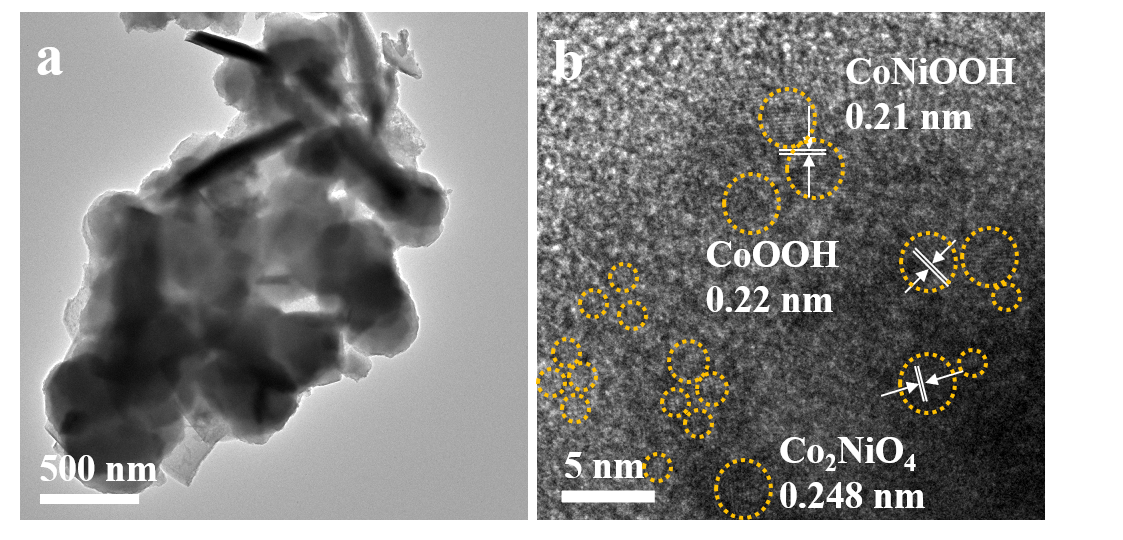


**Figure S38.** The HRTEM images of MOF (Co_4_Ni_1_)-SA1 after 100 h stability testing.


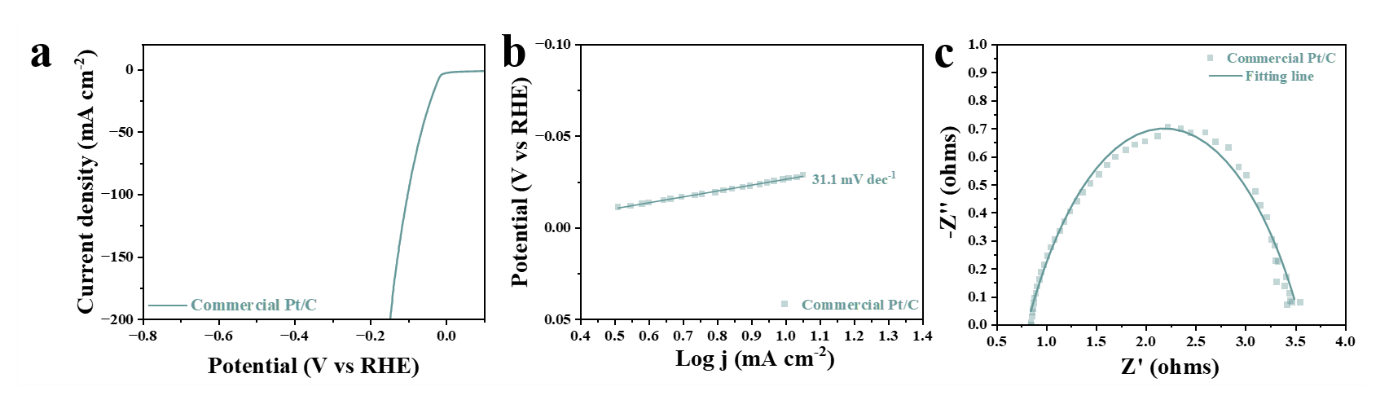


**Figure S39.** (a) The HER polarization curves, (b) Tafel plot, and (c) EIS plot of Commercial Pt/C in 1M KOH.

# Reference

[1] B. Ravel, M. Newville, “ATHENA, ARTEMIS, HEPHAESTUS: data analysis for X-ray absorption spectroscopy using IFEFFIT”, *J. Synchr. Rad.* **2005**, 12, 537-541.

[2] S. I. Zabinsky, J. J. Rehr, A. Ankudinov, R. C. Albers, M. J. Eller, “ Multiple-scattering calculations of X-ray-absorption spectra ”, *Phys. Rev. B* **1995**, 52, 2995-3009.

[3] J. Hafner, “Ab-initio simulations of materials using VASP:: Density-functional theory and beyond”, *J. Comput. Chem.* **2008**, 29, 2044-2078.

[4] P. E. Blochl, “PROJECTOR AUGMENTED-WAVE METHOD”, *Phys. Rev. B* **1994**, 50, 17953-17979.

[5] J. P. Perdew, K. Burke, M. Ernzerhof, “Generalized gradient approximation made simple”, *Phys. Rev. Lett.* **1996**, 77, 3865-3868.

[6] S. Grimme, “Semiempirical GGA-type density functional constructed with a long-range dispersion correction”, *J. Comput. Chem.* **2006**, 27, 1787-1799.

[7] D. J. Chadi, “ *Special points for Brillouin-zone integrations* ”, *Phys. Rev. B* **1977**, 16, 1746-1747.

[8] V. Wang, N. Xu, J. C. Liu, G. Tang, W. T. Geng, “VASPKIT: A user-friendly interface facilitating high-throughput computing and analysis using VASP code”, *Comput. Phys. Commun.* **2021**, 267.

[9] D. D. Liu, S. R. Li, Y. Z. He, C. Liu, Q. L. Li, Y. W. Sui, J. Q. Qi, P. Zhang, C. J. Chen, Z. Chen, S. D. Liu, “Co(OH)F@CoP/CC core-shell nanoarrays for high-performance supercapacitors”, *J. Energy Storage* **2022**, 55.

[10] W. N. Xu, Z. Q. Jiang, Q. Yang, W. C. Huo, M. S. Javed, Y. R. Li, L. Huang, X. Gu, C. G. Hu, “Approaching the lithium-manganese oxides' energy storage limit with Li2MnO3 nanorods for high-performance supercapacitor”, Nano Energy **2018**, 43, 168-176.

[11] S. Q. Niu, S. W. Li, Y. C. Du, X. J. Han, P. Xu, “How to Reliably Report the Overpotential of an Electrocatalyst”, *Acs Energy Lett.* **2020**, 5, 1083-1087.

[12] T. T. Chen, H. Y. Xu, S. P. Li, J. Q. Zhang, Z. C. Tan, L. Chen, Y. W. Chen, Z. J. Huang, H. Pang, “Tailoring the Electrochemical Responses of MOF-74 Via Dual-Defect Engineering for Superior Energy Storage”, *Adv. Mater.* **2024**, 36.

[13] W. Liu, Y. Liang, M. T. Huo, N. Ma, K. C. Qin, J. F. Chang, Z. H. Xing, “Ligand engineering of Co-MOF-74 with hexaaminotriphenylene for enhanced oxygen reduction reaction in zinc-air batteries”, *Nano Res.* **2025**, 18.

[14] H. F. An, W. J. Tian, X. Lu, H. M. Yuan, L. Y. Yang, H. Zhang, H. M. Shen, H. Bai, “Boosting the CO2 adsorption performance by defect-rich hierarchical porous Mg-MOF-74”, *Chem. Eng. J.* **2023**, 469.

[15] T. T. Chen, F. F. Wang, S. Cao, Y. Bai, S. S. Zheng, W. T. Li, S. T. Zhang, S. X. Hu, H. Pang, “In Situ Synthesis of MOF-74 Family for High Areal Energy Density of Aqueous Nickel-Zinc Batteries”, *Adv. Mater.* **2022**, 34.

[16] M. L. He, J. Qiao, B. H. Zhou, J. Wang, S. Guo, G. J. H. Melvin, M. X. Wang, H. Ogata, Y. A. Kim, M. Tanemura, S. Wang, M. Terrones, M. Endo, F. Zhang, Z. P. Wang, “Controllable Metal-Organic Framework-Derived NiCo-Layered Double Hydroxide Nanosheets on Vertical Graphene as Mott-Schottky Heterostructure for High-Performance Hybrid Supercapacitor”, *Small Struct.* **2024**, 5.
